# Synthesis of Thiol Derivatives of Biological Active Compounds for Nanotechnology Application

**DOI:** 10.3390/molecules25153470

**Published:** 2020-07-30

**Authors:** Katarzyna Sidoryk, Olga Michalak, Marek Kubiszewski, Andrzej Leś, Marcin Cybulski, Elżbieta U. Stolarczyk, Jan Doubsky

**Affiliations:** 1 Department of Biomedical Technology, Cosmetic Chemicals and Electrochemistry, Team of Chemistry, Łukasiewicz Research Network—Industrial Chemistry Institute, 8 Rydygiera Str., 01-793 Warsaw, Poland; o.michalak@ifarm.eu (O.M.); m.cybulski@ifarm.eu (M.C.); 2 Analytical Department, Łukasiewicz Research Network—Industrial Chemistry Institute, 8 Rydygiera Str., 01-793 Warsaw, Poland; m.kubiszewski@ifarm.eu (M.K.); e.stolarczyk@ifarm.eu (E.U.S.); 3 Faculty of Chemistry, University of Warsaw, 1 Pasteur Str., 02-093 Warsaw, Poland; ales@tiger.chem.uw.edu.pl; 4 Zentiva k.s., U Kabelovny 130, 102 37 Prague 10, Czech Republic; Jan.Doubsky@zentiva.com

**Keywords:** thiol derivatives, synthetic methodology, theoretical studies, nanoparticles, biological active compounds

## Abstract

An efficient method of thiol group introduction to the structure of common natural products and synthetic active compounds with recognized biological efficacy such genistein (**1**), 5,11-dimethyl-5*H*-indolo[2,3-b]quinolin (**2**), capecitabine (**3**), diosgenin (**4**), tigogenin (**5**), flumethasone (**6**), fluticasone propionate (**7**), ursolic acid methyl ester (**8**), and β-sitosterol (**9**) was developed. In most cases, the desired compounds were obtained easily via two-step processes involving esterification reaction employing S-trityl protected thioacetic acid and the corresponding hydoxy-derivative, followed by removal of the trityl-protecting group to obtain the final compounds. The results of our preliminary experiments forced us to change the strategy in the case of genistein (**1**), and the derivatization of diosgenin (**4**), tigogenin (**5**), and capecitabine (**3**) resulted in obtaining different compounds from those designed. Nevertheless, in all above cases we were able to obtain thiol-containing derivatives of selected biological active compounds. Moreover, a modelling study for the two-step thiolation of genistein and some of its derivatives was accomplished using the density functional theory (B3LP). A hypothesis on a possible reason for the unsuccessful deprotection of the thiolated genistein is also presented based on the semiempirical (PM7) calculations. The developed methodology gives access to new sulphur derivatives, which might find a potential therapeutic benefit.

## 1. Introduction

The sulfur atoms are important structural motifs presented in a number of biologically active natural β-lactam-antibiotics such as penicillin and fusaperazine as well as synthetic drugs, like amoxicillin, cefoxitin, and thienamicin for treatment in bacterial infections [1]. Moreover, sulfur-containing compounds have been utilized as excellent ligands for bounding to metal nanoparticles, especially to Au-nanopraticles, because of a very strong interaction of sulfur nucleophiles with gold nanoparticles [2,3], and gold surface monolayers [4]. The use of the thiol groups to link biologically active compounds to gold nanoparticles is well known and established [5,6]. It is known, that such conjugates may improve therapeutic efficacy of many drugs by: (i) transporting the drug directly to the target/receptor, (ii) increasing solubility in water, (iii) stability, (iv) bioavailability, and (v) by increasing the cell membrane permeability [7]. Furthermore, the gold nanoparticles coated through a terminal sulfur atom with selected proteins have already found an application in studies of organogenesis [8] and also in clinical diagnostics [9].

**Figure 1:**
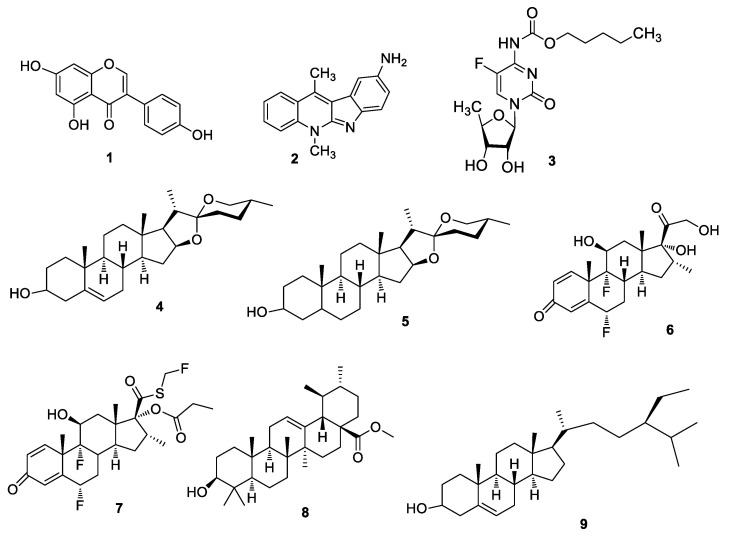
Structures of active compounds: **1**—genistein, **2**—9-amino-5,11-dimethyl-5*H*-indolo[2,3-b]quinoline, **3**—capecitabine, **4**—diosgenin, **5**—tigogenin, **6**—flumethasone, **7**—fluticasone propionate, **8**—ursolic acid methyl ester, **9**—β-sitosterol.

Therefore, the synthesis of thiol-containing compounds is a very important field of interest, but still a challenging one, because of the known sensitivity of thiol-groups towards oxidation (resulting in disulfide bond formation) and a relatively high reactivity of thiol-groups towards alkylating and/or acylating reagents [10]. In the present study, an efficient method of thiol-group introduction to the structure of common natural products and synthetic active compounds with recognized biological efficacy such as: genistein (**1**), 5,11-dimethyl-5*H*-indolo[2,3-b]quinolin (**2**), capecitabine (**3**), diosgenin (**4**), tigogenin (**5**), flumethasone (**6**), fluticasone propionate (**7**), ursolic acid methyl ester (**8**), and β-sitosterol (**9**) was developed (Figure 1). According to the literature, compounds **1**–**9** and their respective derivatives show anticancer [11,12,13], antibacterial [14], antifungal [15], and anti-inflammatory [13,15,16] activities. Some of them are widely used in therapy as the registered drugs: flumethasone [17], fluticasone propionate [18], and capecitabine [19,20].

Genistein which is one of the best investigated natural compounds, which deserves special attention. It shows potent anticancer activity but poor bioavailability, which the latest studies confirm [21,22]. Moreover, this isoflavone, which was isolated in 1899 from a flowering plant *Genista tinctoria*, exhibits other important activities, such as antiangiogenic, antioxidant, and anti-inflammatory activities. Unfortunately, despite interesting biological properties, the clinical application of genistein is still limited mainly because of its low solubility in water [23].

A similar problem occurs with 5,11-dimethyl-5*H*-indolo[2,3-b]quinoline, which also is a natural compound. Many derivatives of 5,11-dimethyl-5*H*-indolo[2,3-b]quinoline were synthetized for improving its therapeutic effect. The best results were achieved when selected amino acids or peptides were linked to an indolo[2,3-b]quinoline core. In vitro and in vivo studies proved that these new conjugates exhibit a higher anticancer activity against A549, MCF-7, and LoVo cells compared to unsubstituted indolo[2,3-b]quinolone [12], but the problem of the low bioavailability has not been resolved so far.

Collectively, the bioavailability and biological activity of genistein, 5,11-dimethyl-5*H*-indolo[2,3-b]quinoline, and all above compounds (Figure 1) can be improved by chemical modification of their structures as well as creating nanoparticles with gold nanoparticles (AuNPs). Our initial study indicated that genistein conjugated with AuNPs achieved higher levels of cytotoxicity compared with free genistein [24]. This encourages us to intensify studies on AuNPs-Genistein as a candidate enhancing the anticancer effect of genistein. Unfortunately, the physical interaction of most of the drugs with the surface of the gold nanoparticle [24,25,26] is typically rather weak and unstable. Consequently, drugs on the nanoparticle may be replaced by other compounds that interact much more strongly with the nanoparticle surface. This can also lead to the aggregation of nanoparticles. Therefore, we focused on the synthesis of biological active compounds modified by a thiol-bearing linker as new derivatives.

The compounds **1**–**9** mentioned above were selected for our studies with respect to important criteria; first of all, their known biological activities. Secondly, some of them have unsatisfactory properties limiting their use as drugs, i.e., bioavailability [23], water solubility [27], and high toxicity which eventually limit their therapeutic window [28]. These parameters could be improved by incorporation of a thiol linker into their structures, which might also serve an opportunity for the future synthesis of gold nanoparticles (AuNPs) via S-Au covalent bonds. These gold nanoparticle-based drug delivery systems may overcome the limitations related to toxicity [29] and enhance the bioactivity of drugs as well [30].

In present work we focused on developing a methodology to attach a thiomethylcarbonyl linker via ester or amide bonds in order to synthesize the thiol-containing derivatives of compounds **1**–**9** as promising substrates for a AuNPs-based drug delivery system. In most cases, the expected products were obtained easily by a two-step process involving esterification reaction of trityl-protected 2-thioacetic acid, followed by the removal of the protecting group to obtain the final compounds. The results of our preliminary experiments forced us to the change this strategy for genistein (**1**) and the derivatization of diosgenin (**4**), tigogenin (**5**), and capecitabine (**3**). In these cases, unexpected products, different from the designed ones, were obtained to our surprise. Nevertheless, in all cases, we were able to obtain new thiol-containing derivatives of the selected biologically active compounds.

## 2. Results and Discussion

### 2.1. Chemistry

**Scheme 1:**
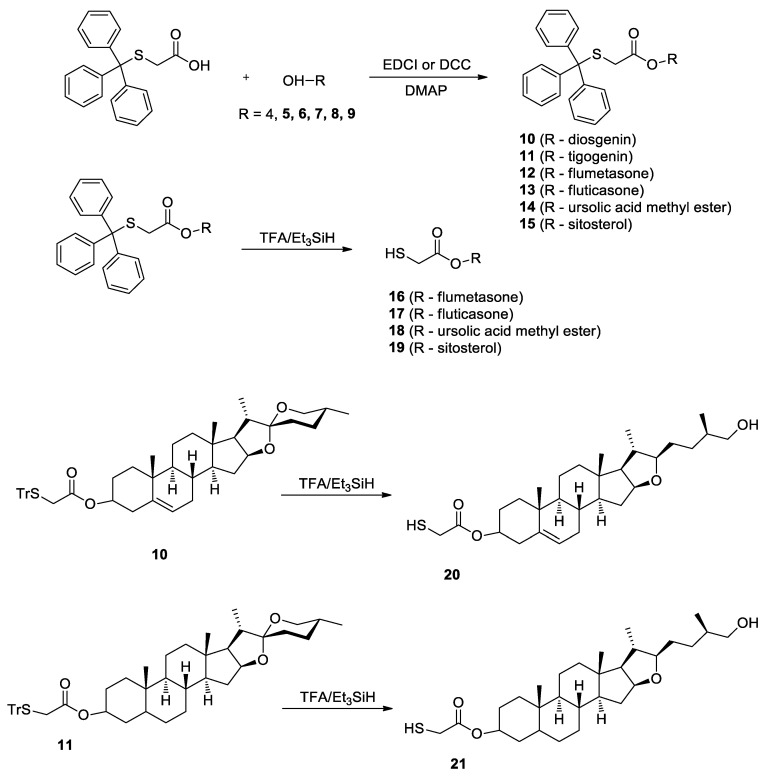
General method of the synthesis of thiol derivatives of flumethasone (**16**), fluticasone propionate (**17**), ursolic acid methyl ester (**18**), β-sitosterol (**19**), diosgenin (**20**), and tigogenin (**21**).

**Figure 2:**
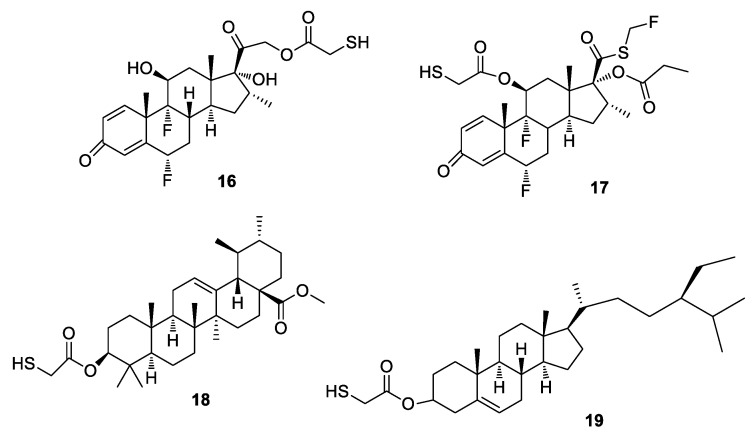
Structures of thiol derivatives of flumethasone (**16**), fluticasone propionate (**17**), ursolic acid methyl ester (**18**), and β-sitosterol (**19**).

The synthesis of thiol derivatives of compounds **4**, **5**, **6**, **7**, **8**, and **9** is presented in Scheme 1. The coupling of diosgenin (**4**), tigogenin (**5**), flumethasone (**6**), fluticasone propionate (**7**), ursolic acid methyl ester (**8**), and β-sitosterol (**9**) with the 2-(tritylthio)acetic acid, in the presence of DCC (*N*,*N*’-dicyclohexylcarbodiimide) or EDCI (*N*-ethyl-*N*’-(3-dimethylaminopropyl)carbodiimide hydrochloride) was performed according to our previously described method [4]. The esterification afforded the desired products in high yields (70 to 98 %). The only exception represents tigogenin (**5**) where the esterification provided the product in moderate yield of 54 %. The crude products were purified by column chromatography on silica gel to obtain the products **10**–**15** as white solids or foams. The deprotection of compounds **10**–**15** was performed using a TFA (trifluoroacetic acid)/Et_3_SiH (triethylsilane) system at 0 °C under nitrogen atmosphere within 30–60 min (thin layer chromatography (TLC) monitoring), followed by triethylamine quenching. The final products **16**–**21** (Scheme 1, Figure 2) were obtained with moderate to high yield in a range of 51 to 93%. As it was confirmed by 1D and 2D NMR experiments for **20** and **21** compounds, trityl-group removal under mild acidic condition at room temperature resulted in additional spiroketal ring opening. Our results were consistent with those known from the literature [13,31,32], where diosgenyl and tigogenin analogs with opened F-ring exhibited high biological activity [13,31,32]. Therefore, we decided to include these two derivatives (**20** and **21**) into further investigation as well. The structures of all new compounds (**9**–**21**) were confirmed unambiguously by extended 1D and 2D NMR experiments (see Experimental Section, and Supplementary Materials) as well as HRMS.

**Scheme 2:**
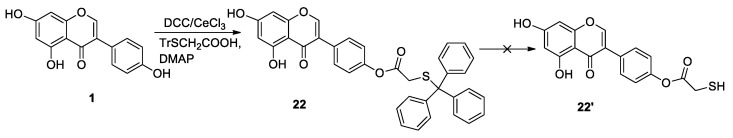
Synthesis of the tritylthiol derivative of genistein (**1**).

Unfortunately, our efficient two-step procedure of thiol derivative preparation failed in the case of genistein (**1**) completely, and no product of 2-(tritylthio)acetic acid esterification was detected. Despite the use of higher equivalents of DCC or EDCI, the elongation of reaction time, the change of solvent (DCM (dichloromethane), THF, DMF), or the temperature increase, only the unreacted substrates were observed in the reaction mixture. Perhaps, the difficulty to directly produce genistein (**1**) ester is caused by lower nucleophilicity of the phenolic OH-groups and/or by inter- and intramolecular hydrogen bond formation (for example between 5-OH and 3-C=O). This effect may be partially neutralized by the complexation with cerium chloride which lead to the rearrangement of charge densities in substrates of esterification. Moreover, the phenolic group of genistein (**1**) is sensitive toward oxidizing agents and to bases which could negatively influence the esterification process. After a rigorous literature search, only few examples of phenolic hydroxyl groups direct esterification were found by using Me_2_NSO_2_Cl [33], montmorillonite-Ti^4+^ [34], metal triflates [35], TiO(acac)_2_ [36], diarylammonium arensulfate [37], and tosyl chloride under solvent-free conditions [38]. Recently, the efficient new methodology of carboxylic acid esterification with phenols was described by the groups of Gilles and Hano [39,40]. The authors performed studies of juglone esterification with different fatty acids using DCC in the presence of DMAP (4-(dimethylamino)pyridine) as a condensation agents (Steglich esterification) with additional Lewis acid as a catalyst (cerium chloride CeCl_3_). The Gilles research proved that the cerium chloride, a very oxophilic Lewis acid, easy to handle and with low toxicity, considerably increased the yield of esterification. This work inspired us to follow the protocol for synthetizing tritythiol derivatives of **1**. The addition of CeCl_3_ to the reaction mixture gave the expected product **22** with a moderate yield of 40% (Scheme 2). The **22** the structure was confirmed by NMR and HRMS studies. It is worth noting that according to this methodology, the ester **22** was prepared in one step. Thus, the preparation of appropriate acyl chloride from 2-(tritylthio)acetic acid and toxic reagents like SOCl_2_ and pyridine was excluded from the procedure.

Encouraged by the above result, we subjected the protected derivative **22** to a deprotection reaction under the same as conditions as before (TFA/Et_3_SiH). Surprisingly, we were able to detect only the starting material in the reaction mixture. Even changes of the reaction conditions (prolonged reaction time, increased temperature up to 60 °C) had no influence on the reaction course. Therefore, we decided to study a new reagent system including both Lewis and protic acids according to the procedure described by the Herczegh group [41]. The detritylation of **22** was carried out using a cocktail containing boron trifluoride diethyl etherate (BF_3_·Et_2_O) as a Lewis acid, 1,1,1,3,3,3-hexafluoroisopropanol (HFIP) as a mild protic acid, and triethylsilane (Et_3_SiH) as a reducing agent. Unfortunately, the treatment of **22** with this three-component cocktail led only to a decomposition of the starting material (even when the amount of BF_3_·Et_2_O was decreased to 0.065 equiv.). We also examined another mild detritylation method using p-toluenesulfonic acid, but the results were unsatisfactory again.

**Scheme 3:**
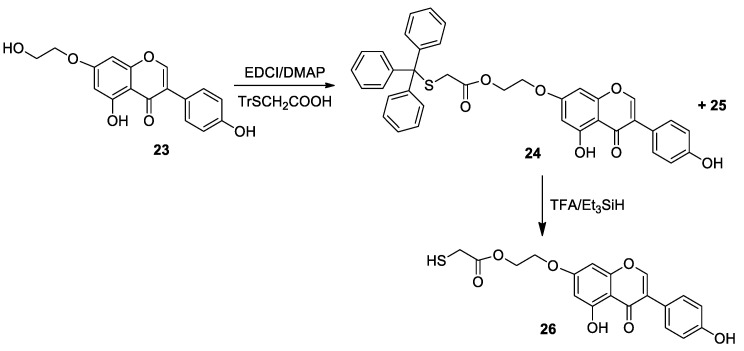
Synthesis of thiol derivative of genistein.

**Figure 3:**
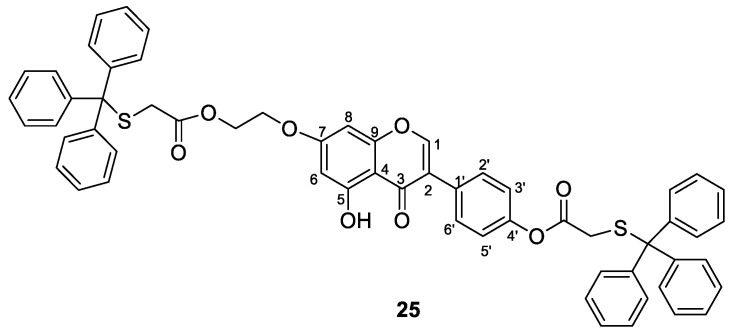
Structure of the impurity **25**.

The failure of **22** deprotection prompted us to design another thiol-derivative of genistein (**1**), i.e., compound **26** (Scheme 3). In order to achieve this goal, we synthesized the known analogue of genistein (**23**) with hydroxyethyl substituent at 7-OH according to the procedure by the Grynkiewicz group [42,43]. Subsequently, **23** was transformed into the tritylthiol derivative (**24**) using our standard procedure (Scheme 3) with a yield of 80%. The traces of di-substituted **25** was also isolated from the crude material (Figure 3) and characterized by NMR technique. Consequently, the intermediate **24**, upon treatment with the mixture of TFA/Et_3_SiH, gave the desired product **26** in 80% yield. The structure was determined by NMR and HRMS analyses. This result proved clearly that this simple experimental protocol works very effectively for the aliphatic hydroxyl group separated from the chromenone ring of genistein.

**Scheme 4:**
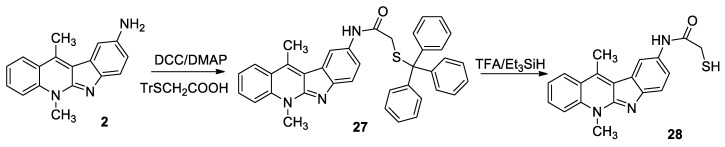
Synthesis of thiol derivative of 5,11-dimethyl-5*H*-indolo[2,3-b]quinoline.

**Scheme 5:**
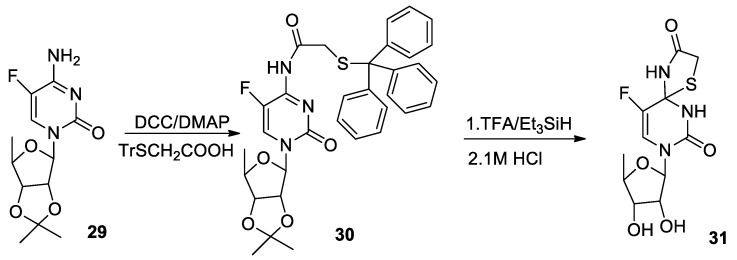
Synthesis of thiol derivative of capecitabine.

The syntheses of the thiol derivatives of 5,11-dimethyl-5*H*-indolo[2,3-b]quinoline (**2**) and capecitabine (**3**) are depicted in (Scheme 4) and (Scheme 5). In the first step of **28** synthesis, the amino group of **2** reacts with TrSCH_2_COOH under standard conditions affording thus the amide **27** in 95% yield. The subsequent deprotection using a TFA/Et_3_SiH system provided the product **28** in 84% yield. It should be noted that we did not observe any cleavage of the amide bond during the deprotection.

In an effort to obtain a thiol derivative of capecitabine (**3**), we utilized its known derivative **29** (Scheme 5) [19]. To our satisfaction, the acylation of **29** yielded **30** in 73% yield smoothly. The complete deprotection of trityl and isopropylidene protecting groups was achieved by a two-step protocol. In the first step, the trityl group was removed using TFA/Et_3_SiH, and the thus obtained crude material (containing traces of the compound **31** already) was treated with 1M HCl to complete the isopropylidene group removal. The thus obtained product **31** was purified by column chromatography, and its structure was determined by NMR studies. It was found that the compound is a spiro-compound containing an additional oxothiazolidine ring. This ring closure results likely from the thiol-group formed as an intermediate in addition to the tautomeric imine type bond in capecitabine derivative under the acid-catalyzed conditions. The reactions with electron-rich as well as electron-deficient aromatic *N*-acyl imines with a broad range of aliphatic and aromatic thiols are well known and were reported, e.g., by Antilla and co-workers [44], the Masuda group [45], and Zhao [46].

We believe that even compound **31** might be capable as a ligand creating metal nanoparticles with gold, platinum, or silver. Therefore, we decided to involve this derivative into our further investigation. The structures of all new compounds were confirmed by NMR as well as HRMS studies.

### 2.2. Theoretical Models of Synthesis of Thiol-Genistein Derivatives

The protection of genistein (**1**) with the tritylthiol reagent (Scheme 2) was modeled with the following equation:

**1** + TrSCH_2_COOH + DCC → **22** + dicyclohexylurea (1)

According to the calculated difference of the Gibbs free energy (details in the Table S1) of molecules composing products and substrates of this equation, the reaction energy output of about −43.0 kcal/mol was obtained, indicating a spontaneous reaction. However, in our laboratory, it was observed that the above reaction does not proceed under standard conditions. Fortunately, after addition of the CeCl_3_ catalyst, this deprotection reactions afforded high yield, and this corroborates the theoretical predictions based on thermodynamic grounds. The molecular model of the role of the CeCl_3_ catalyst is to be investigated. One can hypothesize that the cerium cation coordinates the reagents which are now positioned close together facilitating the reaction.

The reaction of the genistein derivative (**23**) following the Scheme 3 (top) was modeled with the equation:

**23** + TrSCH_2_COOH + EDCI → **24** + urea-derivative (2)

The Gibbs free energy output was calculated to be about −30.8 kcal/mol. Again, this reaction was predicted to go spontaneously. In the course of the synthesis of **24**, the impurity **25** (Figure 3) was found. Both terminal hydroxyl groups in **25** are protected by tritylthiol reagent. The corresponding model reaction is given below.

**23** + 2 TrSCH_2_COOH + 2 EDCI → **25** + 2 urea-derivative (3)

A Gibbs free energy output of about −54.6 kcal/mol was calculated, again pointing to a spontaneous reaction. Thus, the theoretically predicted spontaneous model reactions of **23** with TrSCH_2_COOH and EDCI corroborate laboratory observations.

An intriguing case of deprotection failure of **22** under commonly used reagents TFA/Et_3_SiH prompted us to take a deeper look into the molecular mechanism of the reaction.

**Scheme 6:**
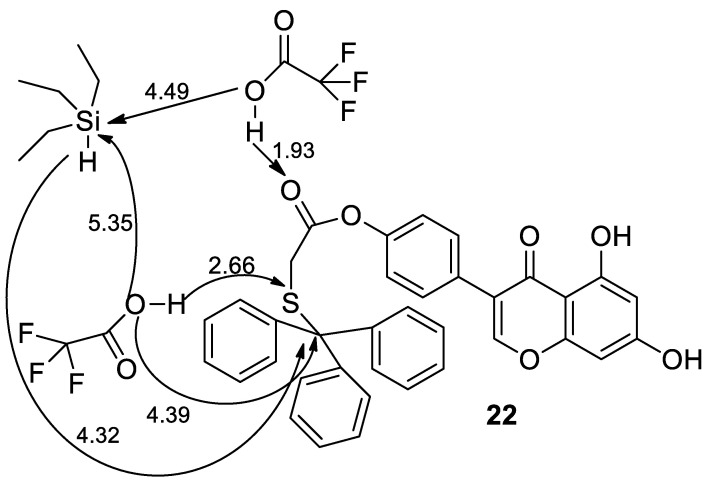
Proposed mechanism of blocking the **22** deprotection. Initially, a weak C(trityl)---S bond is broken, leading to the formation of triphenylcarbenium cation which is supposed to join the TFA anion and form the TrTFA molecule. The H-bonded TFA molecule blocks the approach of a hydrophobic Et_3_SiH towards the TrTFA molecule. The Et_3_SiH molecule is located too far from this TrTFA molecule to complete the deprotection which arrested.

**Scheme 7:**
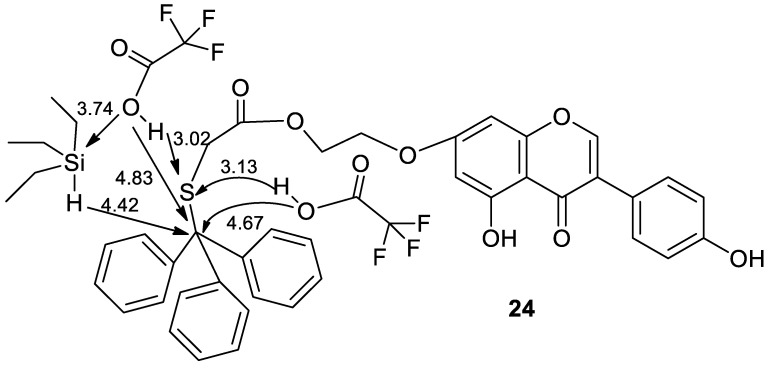
Proposed mechanism of **24** deprotection. There are two TFA molecules around the Tr-S-CH_2_- residue of **24** pointing their hydrogens towards the S atom and weakening the C(trityl)-S bond. There is more freedom around the Tr-S-CH_2_- residue making possible the access of TFA anion and Et_3_SiH molecules to **24** and promoting the deprotection reaction.

A simple look at the chemical formula of **22** and **24** suggests that the -CH_2_-CH_2_-O- linker in **24** removes a possible sterical hindrance of the deprotection reaction. It is a problem that the phenyl group does not interact directly with the reaction center being the Tr-S bond. An insertion of the -CH_2_-CH_2_-O- linker in **24** has likely another role different from a simple alleviation of the phenyl ring. In a search for the molecular basis of deprotection hindrance in **22**, two mechanisms were suggested, one for a hypothetical deprotection of **22** (Scheme 6) and another one for **24** (Scheme 7). Details are given below, but we briefly summarize our idea here. We think the linker can influence on the spatial rearrangement of the TFA molecules around the reaction center. As a consequence of a TFA rearrangement in **22** different from **24**, a subsequent attack of Et_3_SiH on TrTFA is blocked in **22**, but it is allowed in **24**. One can therefore say that the linker influences indirectly the deprotection reaction. Now, some quantum mechanical considerations are presented.

At first the following model reactions were considered:

**22** + TFA + Et_3_SiH → **22′** + CH(Ph)_3_ + CF_3_COOSiEt_3_ (4)

**24** + TFA + Et_3_SiH → **26** + CH(Ph)_3_ + CF_3_COOSiEt_3_ (5)

Following the results of the present calculations, one can suggest that for both model reactions, the Gibbs energy out becomes nearly identical, i.e., −47.3 kcal/mol and −47.8 kcal/mol for the reaction with **22** and **24**, respectively. As long as the deprotection of **22** is not observed experimentally, one may consider the presence of a certain steric hindrance blocking the reaction.

In the search of such a steric hindrance, our attention was focused on an interesting paper of Imagawa et al. 2003 [47]. In this paper, a mechanism of triethylsilyl triflate-catalyzed reductive cleavage of trityl ether (R-O-Tr) with triethylsilane was proposed. An original idea was promoted of an equilibrium between trityl ether and triethylsilyl ether as well as a consecutive fast formation of triphenylmethane and alcohols R-OH (R being e.g., various pivaloyl or benzoyl groups). Taking into account a formal similarity of R-O-Tr and R-S-Tr structures, two model reactions were proposed in the form of (Equation (1)) and (Equation (2)):

R-S-Tr + HO-CO-CF_3_ → R-S-H + Tr-O-CO-CF_3_ (6)

Tr-O-CO-CF_3_ + Et_3_SiH → CH(Ph)_3_ + CF_3_COOSiEt_3_ (7)

where R denotes the **22** or **24** residues. Following the calculated Gibbs free energies of the reaction components, it was predicted that the reaction of Equation (1) becomes nearly reversible based on the Gibbs free energy output of -3.8 kcal/mol (R = **22**) and −4.3 kcal/mol (R = **24**). The reaction (Equation (2)) should be strongly shifted to the right as it can be concluded from the Gibbs free energy output of about −43.5 kcal/mol. In view of the above model reactions, one can suggest that the yet unknown steric hindrance should affect the components shown in the reaction (Equation (1)). The Gibbs free energy output of the reaction (Equation (1)) is small and likely to be sensitive to various intermolecular interactions in the reaction mixture.

Simplified 3D models of ternary complexes [**22**---TFA---Et_3_SiH] and [**24**---TFA---Et_3_SiH] were designed in order to find possible reaction paths leading to the products. Tentatively, it was assumed that the deprotection reaction runs according to the following steps:

Step 1: TFA approaches the thiol-residue of **22** or **24**. The COOH residue of TFA forms a H-bond with the thiol sulfur atom. It occurs a proton transfer along this H-bond towards the sulfur atom. As a result, the S-C(trityl) bond is weakened and finally becomes broken. In this way, the trityl carbocation is formed and then joined to the TFA anion. At this step, the (hypothetical) **22′** or **26** should be formed. We take into account an experimental observation that the deprotection product of **22**, i.e., **22′** is not formed. This fact will be commented on later in the text.

Step 2: Et_3_SiH approaches Tr-O-CO-CF_3_ from the trityl-side pointing the Si-H bond towards the trityl carbon atom. The hydrogen atom, in fact, the hydride H(−), leaves the Et_3_SiH and is attached to the trityl carbocation forming trityl methane. Simultaneously, the Et_3_Si(+) carbocation moves towards the O-CO-CF_3_(-) anion forming Et_3_Si-O-CO-CF_3_.

Scheme 6 and Scheme 7 present the basis ideas of the proposed deprotection mechanism of **22** and **24**.

This hypothesis is based on three prerequisites: the C(trityl)-S bond must be weakened and then broken, the Et_3_SiH should approach the C(trityl) carbocation, and the resulting Et_3_Si carbocation should approach the TFA anion. The analysis of geometry of the **22**---Et_3_SiH---(TFA)_4_ complex, Scheme 6, reveals a rather long intermolecular distance between Et_3_SiH and one of the TFA molecules supposed to create a TFA anion (5.35 Å between the Si atom and the oxygen of the carboxyl group of TFA). It is then reasonable to conclude that the expected reaction between Et_3_SiH and TFA will not occur, and therefore, the deprotection reaction will be arrested. The case of the deprotection of **24** is somewhat different. In the **24**---Et_3_SiH---(TFA)_4_ complex the Et_3_SiH and TFA intermolecular distance is considerably shorter (3.74 Å between the Si atom and the oxygen of the carboxyl group of TFA). Moreover, the sulfur atom of the C(trityl)-S residue of **24** is H-bonded to two TFA molecules that facilitates the break of the C(trityl)-S bond.

The distances in Å correspond to the molecular complex of **22**---Et_3_SiH---(TFA)_4_ and **24**---Et_3_SiH---(TFA)_4_ optimized with the quantum mechanical semiempirical PM7 method. Details of the 3D structures are given in the Supplementary Materials.

In the deprotection reaction, four components are present: **22** or **24**, TFA, TrTFA, and Et_3_SiH. It is assumed that before the reaction is initiated, three components, i.e., **22** or **24,** TFA, and Et_3_SiH should meet together forming intermolecular complexes. The TrTFA component is created in the course of reaction. Molecular models of such a complexes were designed and are composed of the following six molecules:

Complex 1: **22** + Et_3_SiH + 4 TFA (8)

Complex 2: **24** + Et_3_SiH + 4 TFA (9)

The 3D structures were obtained with the use of the quantum mechanical semiempirical PM7 method [48] and are available in the Supplementary Material as the Figure S1.xyz and Figure S2.xyz files for the Complex 1 and Complex 2, respectively.

The four TFA molecules represent a high concentration of trifluoroacetic acid used experimentally in the course of the deprotection reaction. One can observe in Complex 1 the formation of a medium strength hydrogen bond S---H(OOCCF_3_) and relatively large interatomic distance of (Et_3_Si)H---(CPh_3_) and Et_3_Si---O(COCF_3_) of 1.93, 4.32, and 4.49 Å, respectively. In Complex 2, the corresponding interatomic distances are 3.02, 4.42, and 3.74 Å. It is remarkable that a larger interatomic distance of Si---O (4.49 Å) in Complex 1 is found when compared to Si---O (3.74 Å) in Complex 2. Perhaps it is an indication of a weaker intermolecular interaction of Et_3_SiH with solvated (by TFA) **22** molecule. The product of deprotection can be also modeled, and it is presented in the files available in the Supplementary material as the Figure S3.xyz and Figure S4.xyz for the hypothetical product or disintegration of the Complex 1 and for disintegration of the Complex 2, respectively.

Both product structures are not yet optimized. They are presented here for a tentative view of how the atom rearrangement would proceed. The energy optimization of the Complex 1 or Complex 2 does not lead by itself to the deprotection products. One can conclude that when modeling the deprotection reaction, one needs to put some impulse to start atom rearrangement. Such an energetical impulse can originate from the thermal motions or intermolecular collisions of the TFA molecules surrounding **22** or **24**. More detailed studies of these motions demand not only a static approach as presented here but rather molecular dynamics including chemical reactions with the quantum chemical approach for **22** and **24** with Et_3_SiH in the bath of TFA molecules. We think this is a line for the future studies.

## 3. Materials and Methods

### 3.1. General

The ^1^H and ^13^C NMR spectra of all compounds were measured using Bruker AVANCE III HD spectrometer at the 500 MHz transmitter frequency for ^1^H at a temperature of 298 K. The spectra were measured at a temperature of 295 K in the CDCl_3_ solution relatively to the TMS signal as a ^1^H and ^13^C chemical shift standard. The NMR studies were measured using the results from one- and two-dimensional NMR spectroscopy: ^1^H, ^13^C, HSQC, HMBC, and COSY. The ESI-MS spectra were recorded on a PE Biosystems Mariner mass spectrometer. The progress of the reaction was monitored by thin layer chromatography (TLC) with Merck DC-Alufolien Kieselgel 60 F_254_. The chemicals and solvents were purchased from Fluka Company. Column chromatography was performed on Merck silica gel 60 (230–400 mesh).

### 3.2. General Procedure for the Synthesis of Compounds **10–15, 24, 27, 30**

Compound **2**, **4**, **5**, **6**, **7**, **8**, **9**, **23**, or **29** (1 mM) and Tr-S-CH_2_-COOH (1.2 mM) were dissolved in 10 mL dichloromethane. Then DMAP (1.5 mM) was added to the solution. To the clear solution, EDCI or DCC (1.5 mM) was added portion-wise, and the reaction was stirred for 2–24 h at RT (TLC monitoring: hexane-ethyl acetate). The mixture was treated with 10 mL water. Next, the organic solution was washed with 0.1 M NaOH and brine. The organic phase was dried over anhydrous magnesium sulphate, filtered and concentrated to the crude solid. The crude products were purified by column chromatography (hexane/ethyl acetate 5:1→1:1) to give **10–15**, **24**, **27**, or **30** as white solids or foams.

### 3.3. General Procedure for the Synthesis of Compounds **16–21, 26, 28**

The intermediates **10–15**, **24**, or **27** (1 mM) were dissolved in 10 mL dichloromethane in argon atmosphere and cooled to 0 °C (ice bath). The colorless solution was treated with 2 mL of trifluoroacetic acid, and next, triethylsilane (0.5 mL) was immediately added to the reaction mixture. The reaction was monitored by TLC (hexane:ethyl acetate 1:1 or hexane:ethyl acetate:methanol 5:3:1), and it was completed after 20 min. Then the reaction was quenched with triethylamine (1 mL), and the solution was washed with water (20 mL) and brine (20 mL). The organic phase was evaporated to the oil at room temperature. The crude oil was purified by column chromatography (hexane:ethyl acetate 1:1 or hexane:ethyl acetate:methanol 5:3:1) to afford of **16**, **17, 18**, **19**, **20**, **21**, **26**, or **28** as white solids or foams.

diosgenin 2-(tritylmercapto)acetate (**10**): Yield 83%, m.p. 216.1 °C, ^1^HNMR (500 MHz, CDCl_3_): δ 7.42–7.22 (m, 15H, Ar-H), 5.35 (d, *J* = 5.0 Hz, 1H, H-6), 4.50 (m, 1H, H-3), 4.41 (m,1H, H-16), 3.47 (m, 1H, one of H-27 protons), 3.38 (t, *J* = 11.0 Hz, 1H, one of H-27 protons), 2.92 (s, 2H, -CO-*CH_2_*-S-), 2.25 (m, 2H, H-4), 1.99 and 1.54 (m,2H, H-7), 1.99 and 1.28 (m, 2H, m, H-15), 1.88 (m, 1H, H-20), 1.83 and 1.09 (m, 2H, H-1), 1.78 and 1.58 (m, 2H, H-2), 1.79 (m, 1H, H-17), 1.73 and 1.18 (m, 2H, H-12), 1.69 and 1.60 (m, 2H, H-23), 1.63 and 1.46 (m, 2H, H-24), 1.63 (m, 1H, H-25), 1.60 (m, 1H, H-8), 1.49 (m, 2H, H-11), 1.11 (m, 1H, H-14), 1.01 (s, 3H, CH3-19), 0.97 (m, 1H, H-9), 0.97 (d, *J* = 7.0 Hz, 3H, CH3-21), 0.79 (d, 3H, CH3-26), 0.78 (s, 3H, CH3-18);^13^C NMR (125 MHz, CDCl_3_): δ 169.0 (CO), 144.1 (Ar-C), 139.5 (C-5), 129.5–126.8 (Ar-C), 122.5 (C-6), 109.3 (C-22), 80.8 (C-16), 75.0 (C-3), 67.1 (-*C*-Ph), 66.8 (C-27), 62.0 (C-17), 56.4 (C-14), 49.8 (C-9), 41.6 (C-20), 40.2 (C-13), 39.7 (C-12), 37.8 (C-4), 36.8 (C-1), 36.7 (C-10), 35.03(-*CH_2_*-S-), 32.0 (C-7), 31.8 (C-15), 31.4 (C-23), 31.3 (C-8), 30.3 (C-25), 28.8 (C-24), 27.5 (C-2), 20.8 (C-11), 19.3 (C-19), 17.1 (C-26), 16.3 (C-18), 14.5 (C-21). HRMS (ESI) calcd. for C_48_H_58_O_4_SNa (M+Na)^+^: 753.3954. Found: 753.3977.

tigogenin 2-(tritylmercapto)acetate (**11**): Yield 54%, m.p. 230.3 °C, ^1^HNMR (500 MHz, CDCl_3_): δ 7.41–7.22 (m, 15H, Ar-H), 4.59 (m, 1H, H-3), 4.39 (m, 1H, H-16), 3.46 and 3.38 (2xm, 2H, H-27), 2.91 (s, 2H, -CO-*CH_2_*-S-), 1.95 (m, 1H, H-20), 1.76 (m,1H, H-17), 1.53 (m, 1H, H-8), 1.62 (m,1H, H-25), 1.12 (m,1H, H-5), 1.12 (m, 1H, H-14), 0.65 (m, 1H, H-9), 1.97 and 1.24 (2xm, 2H, H-15), 1.74 and 1.43 (2xm, 2H, H-2), 1.69 and 0.97 (2xm, 2H, H-1), 1.66 and 0.89 (2xm, 2H, H-7), 1.52 and 1.29 (2xm, 2H, H-4), 1.69 and 1.13 (2xm, 2H, H-12), 1.67 and 1.59 (2xm, 2H, H-23), 1.62 and 1.46 (2xm, 2H, H-24), 1.48 and 1.28 (2xm, 2H, H-11), 1.26 (2xm, 2H, H-6), 0.96 (d, *J =* 10Hz, 3H, CH3-21), 0.81 (s, 3H, CH3-19), 0.75 (s, 3H, CH3-18), 0.79 (d, *J =* 5Hz, 3H, CH3-26); ^13^C NMR (125 MHz, CDCl_3_): δ 169.1 (CO), 144.1 (Ar-C), 129.6–126.8 (Ar-C), 109.2 (C-22), 80.8 (C-16), 74.8 (C-3), 67.0 (-*C*-Ph), 66.8 (C-27), 62.1 (C-17), 56.2 (C-14), 54.1 (C-9), 44.5 (C-5), 41.6 (C-20), 40.5 (C-13), 40.0 (C-12), 36.6 (C-1), 36.1 (-*CH_2_*-S-), 35.5 (C-10), 35.0 (C-8), 33.7 (C-4), 32.1 (C-7), 31.7 (C-15), 31.3 (C-23), 30.3 (C-25), 28.8 (C-24), 28.4 (C-6), 27.2 (C-2), 21.0 (C-11), 17.1 (CH3-26), 16.5 (CH3-18), 14.5 (CH3-21), 12.2 (CH3-19). HRMS (ESI) calcd. for C_48_H_60_O_4_SNa (M+Na)^+^: 755.4110. Found: 755.4121.

flumethasone 2-(tritylmercapto)acetate (**12**): Yield 98%, m.p. 223.0 °C, ^1^HNMR (500 MHz, CDCl_3_): δ 7.41–7.22 (m, 15H, Ar-H), δ 7.14 (dd, 1H, H-1), 6.40 (bs, 1H, H-4), 6.35 (dd, *J* = 10 Hz, 1H, H-2), 5.41 and 5.31 (2xm, 1H, H-6), 4.82 and 4.72 (2xd, *J* = 30 Hz, 2H, H-22), 4.35 (m, 1H, H-11), 3.11 (m, 2H, -*CH_2_*-S-), 3.0 (m, 1H, H-16), 2.37 and 1.65 (2xm, 2H, H-12), 2.23 and 1.71 (2xm, 2H, H-7), 2.42 (m, 1H, H-8), 2.23 (m, 1H, H-14), 1.24 and 1.17 (2xm, 2H, H-15), 1.50 (s, 3H, CH3-19), 0.99 (s, 3H, CH3-18), 0.90 (d, *J* = 5 Hz, 3H, H-20); ^13^C NMR (125 MHz, CDCl_3_): δ 204.1 (C-21), 185.7 (C-3), 169.8 (CO, -*C*O-CH_2_-S-), 161.65 (d, *J* = 13.7 Hz, C-5), 151.0 (C-1), 143.9 (Ar-C) 130.0 (C-2), 129.5–126.9 (Ar-C), 121.1 (d, *J* = 13.0 Hz, C-4), 98.8 (d, *J* = 177.2 Hz, C-9), 91.00 (C-17), 86.5 (d, *J* = 183.9 Hz, C-6), 71.7 (d, *J* = 38.0 Hz, C-11), 69.2 (C-22), 48.4 (C-13), 48.1 (dd, *J_1_* = 22.6 Hz, *J_2_* = 3.7 Hz, C-10), 43.7 (C-14), 36.2 (C-12), 35.9 (C-16), 33.7 (d, *J* = 19.8 Hz, C-7), 32.7 (dd, *J_1_* = 19.3 Hz, *J_2_* = 11.1 Hz, C-8), 32.0 (C-15), 34.3 (-*CH_2_*-S-), 22.9 (d, *J* = 5.4 Hz, CH3-19), 16.4 (CH3-18), 14.6 (CH3-20); HRMS (ESI) calcd. for C_43_H_44_O_6_F_2_SNa (M+Na)^+^: 749.2724. Found: 749.2747.

11-*O*-[2-(tritylmercapto)acetyl]-fluticasone propionate (**13**): Yield 71%, m.p. 115.1 °C; ^1^HNMR (500 MHz, CDCl_3_): δ 7.36–7.29 (m, 15H, Ar-H), 6.67–6.66 (dd, 1H, H-1), 6.48 (s, 1H, H-4), 6.35–6.34 (dd, 1H, H-2), 5.89–5.88 (m, 1H, -S-CH_2_-F), 5.79–5.78 (m, 1H, -S-CH_2_-F), 5.43–5.42 (m, 1H, H-11), 5.25–5.24 (m, 1H, H-6), 3.40 (brs, 1H, H-16), 2.99–2.98 (m, 2H, -CO-CH_2_-S-), 2.37–2.36 (m, 2H, -CO-C*H*_2_-CH_3_), 2.31 (m, 1H, H-12), 2.32–2.31 (m, 1H, H-8), 2.26–2.27 (m, 1H, H-14), 2.27 (m, 1H, H-7), 1.93 (m, 1H, H-12), 1.84 (m, 1H, H-7), 1.85 (m, 1H, H-15), 1.34 (m, 1H, H-15), 1.11–0.98 (m, 12H, CH_3_); ^13^C NMR (125 MHz, CDCl_3_): δ 192.8 (*C*O-S-CH_2_-F), 184.8 (CO, C-3), 172.7 (O-*C*O-CH_2_-CH_3_), 167.2 (-O-*C*O-CH_2_-S-Tr), 159.7 (d, *J* = 14.5 Hz, C-5), 148.6 (C-1), 143.5 (Ar-C), 130.8 (C-2), 129.4–127.1 (Ar-C), 121.4 (d, *J* = 13.0 Hz, C-4), 97.5 (d, *J* = 179.5 Hz, C-9). 95.9 (C-17), 86.1 (d, *J* = 185.1 Hz, C-6), 80.7 (d, *J* = 216.8 Hz, -S-CH_2_-F), 71.9 (d, *J* = 40.8 Hz, C-11), 67.6 (-*C*-Ph), 47.9 (C-13), 47.1 (dd, *J_1_* = 33.4Hz, *J_2_* = 3.8 Hz, C-10), 42.6 (C-14), 36.1 (C-16), 34.9 (-CO-*C*H_2_-S-Tr), 33.9 (C-15), 33.4 (d, *J* = 20.1 Hz, C-7), 33.1 (dd, *J_1_* = 19.2 Hz, *J_2_* = 11.4 Hz, C-8), 32.7 (C-12), 27.5 (CO-*C*H_2_-CH_3_), 23.1 (d, *J* = 5.2 Hz, CH_3_), 17.1 (CH_3_), 16.2 (CH_3_), 9.0 (CH_3_); HRMS (ESI) calcd. for C_46_H_47_O_6_S_2_ (M+Na)^+^: 839.2684. Found: 839.2664.

3-*O*-[2-(tritylmercapto)acetyl]-ursolic acid methyl ester (**14**): Yield 84%, m.p. 219.3 °C, ^1^HNMR (500 MHz, CDCl_3_): δ 7.41–7.22 (m, 15H, Ar-H), 5.24 (t, 1H, *J =* 5Hz, H-12), 4.46 (m, 1H, H-3), 3.60 (s, 3H, H-31), 2.92 (d, *J =* 5Hz, 2H, -C*H*_2_-S-), 2.23 (d, *J* = 15 Hz, 1H, H-18), 2.00 and 1.68 (2xm, 2H, H-16), 1.90 (m, 2H, H-11), 1.77 and 1.06 (2xm, 2H, H-15), 1.58 (m, 2H, H-2), 1.67 and 1.60 (2xm, 2H, H-22), 1.63 and 1.05 (2xm, 2H, H-1), 1.52 (m, 1H, H-9), 1.51 and 1.36 (2xm, 2H, H-6), 1.48 and 1.30 (2xm, 2H, H-21), 1.48 and 1.31 (2xm, 2H, H-7), 1.34 (m, 1H, H-19), 1.07 (s, 3H, H-27), 1.00 (m, 1H, H-20), 0.93 (s, 3H, CH3-25), 0.94 (d, *J =* 10 Hz, 3H, CH3-30), 0.84 (m, 3H, CH3-23), 0.84 (m, 3H, CH3-24), 0.85 (d, *J =* 5Hz, 3H, CH3-29), 0.80 (m, 1H, H-5), 0.74 (s, 3H, CH3-26); ^13^C NMR (125 MHz, CDCl_3_): δ 178.1 (C-28), 169.4 (CO), 144.1 (Ar-C), 138.2 (C-13), 129.5–126.8 (Ar-C), 125.4 (C-12), 82.1 (C-3), 67.1 (-*C*-Ph) 55.2 (C-5), 52.8 (C-18), 51.4 (C-31), 48.1 (C-17), 47.4 (C-9), 41.9 (C-14), 39.5 (C-8), 39.0 (C-19), 38.8 (C-20), 38.2 (C-1), 37.8 (C-4), 36.8 (C-10), 36.6 (C-22), 35.2 (-C*H*_2_-S-), 32.8 (C-7), 30.6 (C-21), 28.1 (C-23), 27.9 (C-15), 24.2 (C-16), 23.5 (C-27), 23.4 (C-2), 23.3 (C-11), 21.2 (CH3-30), 18.1 (C-6), 17.0 (CH3-29), 16.9 (CH3-26), 16.8 (CH3-24), 15.5 (CH3-25). HRMS (ESI) calcd. for C_52_H_66_O_4_SNa (M+Na)^+^: 809.4580. Found: 809.4597.

β-sitosterol 2-(tritylmercapto)acetate (**15**): Yield 75%, ^1^HNMR (500 MHz, CDCl_3_): δ 7.43–7.23 (m, 15H, Ar-H), 5.36 (d, *J* = 5.0 Hz, 1H, H-6), 4.51 (m, 1H, H-3), 2.93 (s, 2H, -C*H*_2_-S-), 2.25 (d, *J* = 5.0 Hz, 2H, H-4), 2.01 and 1.18 (2xm, 2H, H-12), 1.85 and 1.27 (2xm, 2H, H-16), 1.97 and 1.54 (2xm, 2H, H-7), 0.94 (m,1H, H-9), 1.81 and 1.52 (2xm, 2H, H-2), 1.84 and 1.10 (2xm, 2H, H-1), 1.67 (m, 1H, H-25), 1.58 and 1.07 (2xm, 2H, H-15), 1.49 (m, 2H, H-11), 1.44 (m, 1H, H-8), 1.36 (m, 1H, H-20), 1.33 and 1.02 (2xm, 2H, H-22), 1.27 (m, 2H, H-28), 1.18 (m, 2H, H-23), 1.12 (m, 1H, H-17), 1.00 (m,1H, H-14), 1.00 (s, 3H, CH3-19), 0.94 (m, 1H, H-24), 0.93 (d, *J* = 5 Hz, 3H, CH3-21), 0.85 (m, 3H, CH3-29), 0.85 (m, 3H, H-27), 0.82 (m, 3H, CH3-26), 0.68 (s, 3H, CH3-18); ^13^C NMR (125 MHz, CDCl_3_): δ 169.0 (CO), 139.5 (C-5), 144.1 (Ar-C), 129.6–126.9 (Ar-C), 122.8 (C-6), 75.1 (C-3), 67.1 (-*C*-Ph), 56.7 (C-14), 56.0 (C-17), 50.0 (C-9), 45.8 (C-24), 42.3 (C-13), 39.7 (C-12), 37.9 (C-4), 36.9 (C-1), 36.5 (C-10), 36.2 (C-20), 35.1 (-C*H*_2_-S-), 33.9 (C-22), 31.9 (C-7), 31.8 (C-8), 29.1 (C-25), 28.3 (C-16), 27.6 (C-2), 26.0 (C-23), 24.3 (C-15), 23.1 (C-28), 21.0 (C-11), 19.8 (CH3-27), 19.3 (CH3-19), 19.0 (CH3-26), 18.8 (CH3-21), 12.0 (CH3-29), 11.9 (CH3-18). HRMS (ESI) calcd. for C_50_H_66_O_2_SNa (M+Na)^+^: 753.4681. Found: 753.4700.

flumethasone 2-mercaptoacetate (**16**): Yield 71%, m.p. 244.2 °C, ^1^HNMR (500 MHz, CDCl_3_): δ 7.11 (d, *J* = 5 Hz, 1H, H-1), 6.42 (s, 1H, H-4), 6.37 (dd, *J* = 10 Hz, 1H, H-2), 5.43 and 5.33 (2xm, 1H, H-6), 4.98 and 4.91(2xd, *J* = 20 Hz, 2H, H-22), 4.40 (m, 1H, H-11), 3.42 (m, 2H,-*CH_2_*-SH), 3.13 (m, 1H, H-16), 2.45 and 1.68 (2xm, 2H, H-12), 2.25 and 1.74 (2xm, 2H, H-7), 2.44 (m, 1H, H-8), 2.26 (m, 1H, H-14), 1.80 and 1.17 (2xm, 2H, H-15), 1.52 (s, 3H, CH3-19), 1.04 (s, 3H, CH3-18), 0.93 (d, *J* = 5 Hz, 3H, H-20); ^13^C NMR (125 MHz, CDCl_3_): δ 204.1 (C-21), 185.5 (C-3), 170.9 (CO, -*C*O-CH_2_-SH), 161 (C-5), 150.4 (C-1), 130.3 (C-2), 121.2 (d, *J* = 13.1 Hz, C-4), 98.7 (C-9), 90.9 (C-17), 86.5 (d, *J* = 184.2 Hz, C-6), 71.8 (d, *J* = 38.3 Hz, C-11), 69.3 (C-22), 48.5 (C-13), 48 (C-10), 43.7 (C-14), 36.5 (C-12), 35.9 (C-16), 33.7 (d, *J* = 19.8 Hz, C-7), 33.7 (C-8), 32.0 (C-15), 26.2 (-*CH_2_*-SH), 23.0 (d, *J* = 6.0 Hz, CH3-19), 16.5 (CH3-18), 14.5 (CH3-20); HRMS (ESI) calcd. for C_24_H_30_O_6_F_2_SNa (M+Na)^+^: 507.1629. Found: 507.1650.

11-*O*-[2-(mercapto)acetyl]-fluticasone propionate (**17**): Yield 93%, m.p. 169–170 °C; ^1^HNMR (500 MHz, CDCl_3_): δ 6.90–6.88 (m, 1H, H-1), 6.33–6.31 (m, 1H, H-2), 6.14 (bs, 1H, H-4), 5.93–5.91 (m, 1H, -S-CH_2_-F), 5.83–5.81 (m, 1H, -S-CH_2_-F), 5.67–5.58 (m, 1H, H-6), 5.24–5.23 (m, 1H, H-11), 3.88–3.87 (m, 2H, -C*H*_2_-SH), 3.29 (s, 1H, H-16), 2.65–2.64 (m, 1H, H-8), 2.38–2.36 (m, 2H, -CO-C*H*_2_-CH_3_), 2.28 (m, 1H, H-7), 2.21 (m, 1H, H-12), 2.15–2.13 (m, 1H, H-14), 1.95 (m, 1H, H-12), 1.88 (m, 1H, H-15), 1.53 (m, 1H, H-7), 1.29 (m, 1H, H-15), 1.11–0.98 (m, 12H, CH_3_); ^13^C NMR (125 MHz, CDCl_3_): δ 193.1 (CO, -*C*O-S-CH_2_-F), 183.9 (CO, C-3), 172.3 (CO, -*C*O-CH_2_-CH_3_), 167.7 (CO, -*C*O-CH_2_-SH), 161.6 (d, *J* = 13.8 Hz, C-5), 149.9 (C-1), 129.7 (C-2), 119.7 (d, *J* = 12.5 Hz, C-4), 98.4 (d, *J* = 176.8 Hz, C-9), 95.5 (C-17), 86.4 (d, *J* = 180.8 Hz, C-6), 80.9 (d, *J* = 212.5 Hz-S-C*H*_2_-F), 72.05 (d, *J* = 41.3 Hz, C-11), 47.6 (C-13), 47.2 (dd, *J*_1_ = 22.1 Hz, *J*_2_ = 3.2 Hz, C-10), 42.3 (C-14), 40.5 (-CH_2_-SH), 35.7 (C-16), 33.4 (d, *J* = 19.2 Hz, C-7), 33.1 (C-15), 32.3 (C-8), 31.9 (C-12), 26.8 (-*C*H_2_-CH_3_), 22.3 (d, *J* = 5.2 Hz, CH_3_), 16.7 (CH_3_), 15.7 (CH_3_), 8.9 (CH_3_). HRMS (ESI) calcd. for C_27_H_33_O_6_F_3_S_2_Na (M+Na)^+^: 597.6762. Found: 597.6758.

3-*O*-[2-(mercapto)acetyl]-ursolic acid methyl ester (**18**): Yield 95%, m.p. 155.8 °C, ^1^HNMR (500 MHz, CDCl_3_): δ 5.24 (t, *J =* 5Hz, 1H, H-12), 4.53 (m,1H, H-3), 3.60 (s, 3H, H-31), 3.25 (d, *J =* 5Hz, 2H, -C*H*_2_-SH), 2.23 (d, *J* = 15 Hz, 1H, H-18), 1.99 and 1.65 (2xm, 2H, H-16), 1.90 (m, 2H, H-11), 1.77 and 1.06 (2xm, 2H, H-15), 1.65 (m, 2H, H-2), 1.65 and 1.58 (2xm, 2H, H-22), 1.65 and 1.07 (2xm, 2H, H-1), 1.52 (m,1H, H-9), 1.50 and 1.36 (2xm, 2H, H-6), 1.48 and 1.29 (2xm, 2H, H-21), 1.48 and 1.32 (2xm, 2H, H-7), 1.33 (m, 1H, H-19), 1.07 (s, 3H, H-27), 1.00 (m, 1H, H-20), 0.94 (s, 3H, CH3-25), 0.94 (d, *J =* 5 Hz, 3H, CH3-30), 0.89 (s, 3H, CH3-23), 0.87 (m, 3H, CH3-24), 0.86 (d, *J =* 5Hz, 3H, CH3-29), 0.82 (m, 1H, H-5), 0.74 (s, 3H, CH3-26); ^13^C NMR (125 MHz, CDCl_3_): δ 178.1 (C-28), 170.7 (CO), 138.2 (C-13), 125.4 (C-12), 82.4 (C-3), 55.3 (C-5), 52.8 (C-18), 51.5 (C-31), 48.0 (C-17), 47.4 (C-9), 41.9 (C-14), 39.4 (C-8), 39.0 (C-19), 38.8 (C-20), 38.2 (C-1), 37.9 (C-4), 36.8 (C-10), 36.6 (C-22), 32.8 (C-7), 30.6 (C-21), 28.0 (C-23), 27.9 (C-15), 26.9 (-C*H*_2_-SH), 24.2 (C-16), 23.5 (C-27), 23.4 (C-2), 23.3 (C-11), 21.2 (CH3-30), 18.1 (C-6), 17.0 (CH3-29), 16.9 (CH3-26), 16.7 (CH3-24), 15.5 (CH3-25). HRMS (ESI) calcd. for C_33_H_52_O_4_SNa (M+Na)^+^: 567.3484. Found: 567.3475.

β-sitosterol 2-mercaptoacetate (**19**): Yield 51%, m.p. 166.7 °C, ^1^HNMR (500 MHz, CDCl_3_): δ 5.39 (d, *J* = 5.0 Hz, 1H, H-6), 4.64 (m, 1H, H-3), 3.22 (m, 2H, -C*H*_2_-SH), 2.34 (m, 2H, H-4), 2.01 and 1.17 (2xm, 2H, H-12), 1.84 and 1.26 (2xm, 2H, H-16), 1.97 and 1.56 (2xm, 2H, H-7), 1.95 (m,1H, H-9), 1.88 and 1.62 (2xm, 2H, H-2), 1.88 and 1.14 (2xm, 2H, H-1), 1.67 (m, 1H, H-25), 1.58 and 1.05 (2xm, 2H, H-15), 1.49 (m, 2H, H-11), 1.46 (m, 1H, H-8), 1.36 (m, 1H, H-20), 1.33 and 1.01 (2xm, 2H, H-22), 1.26 (m, 2H, H-28), 1.16 (2xm, 2H, H-23), 1.11 (m, 1H, H-17), 1.01 (m, 1H, H-14), 1.02 (s, 3H, CH3-19), 0.92 (m, 1H, H-24), 0.92 (d, *J* = 5 Hz, 3H, CH3-21), 0.84 (m, 3H, CH3-29), 0.84 (m, 3H, H-27), 0.81 (m, 3H, CH3-26), 0.68 (s, 3H, CH3-18); ^13^C NMR (125 MHz, CDCl_3_): δ 170.3 (CO), 139.3 (C-5), 122.9 (C-6), 75.4 (C-3), 56.6 (C-14), 56.0 (C-17), 50.0 (C-9), 45.8 (C-24), 42.3 (C-13), 39.7 (C-12), 37.9 (C-4), 36.9 (C-1), 36.5 (C-10), 36.1 (C-20), 33.9 (C-22), 31.9 (C-7), 31.8 (C-8), 29.1 (C-25), 28.2 (C-16), 27.6 (C-2), 26.9 (-C*H*_2_-SH), 26.0 (C-23), 24.3 (C-15), 23.0 (C-28), 21.0 (C-11), 19.8 (CH3-27), 19.3 (CH3-19), 19.0 (CH3-26), 18.7 (CH3-21), 12.0 (CH3-29), 11.8 (CH3-18). HRMS (ESI) calcd. for C_31_H_52_O_2_SNa (M+Na)^+^: 511.3586. Found: 511.3596.

3-*O*-[2-(mercapto)acetyl]-(3β, 25*R*)–furost–5–ene-3,26-diol (**20**): Yield 81%, m.p. 108.1 °C, ^1^HNMR (500 MHz, CDCl_3_): δ 5.38 (d, *J* = 5.0 Hz, 1H, H-6), 4.65 (m, 1H, H-3), 4.31 (m, 1H, H-16), 3.47 (m, 2H, m, H-27), 3.33 (m, 1H, H-22), 3.23 (d, *J* = 10 Hz, 2H, -C*H*_2_-SH), 2.35 (m, 2H, H-4), 2.00 and 1.30 (2xm, 2H, H-15), 2.00 and 1.54 (2xm, 2H, H-7), 1.89 and 1.62 (2xm, 2H, H-2), 1.89 and 1.13 (2xm, 2H, H-1), 1.75 (m, 1H, H-20), 1.72 and 1.12 (2xm, 2H, H-12), 1.66 (m, 1H, H-25), 1.62 (m, 1H, H-8), 1.61 (m, 1H, H-17), 1.59 (m, 2H, H-23), 1.48 (m, 2H, H-11), 1.47 and 1.35 (2xm, 2H, H-24), 1.09 (m, 1H, H-14), 1.03 (s, 3H, CH3-19), 1.00 (d, *J* = 5 Hz, 3H, CH3-21), 0.95 (m, 1H, H-9), 0.91 (d, *J* = 10 Hz, 3H, CH3-26), 0.81 (s, 3H, CH3-18); ^13^C NMR (125 MHz, CDCl_3_): δ 170.5 (CO), 139.3 (C-5), 122.6 (C-6), 90.4 (C-22), 83.2 (C-16), 75.3 (C-3), 68.0 (C-27), 65.0 (C-17), 56.9 (C-14), 50.0 (C-9), 40.7 (C-13), 39.4 (C-12), 37.9 (C-20), 37.8 (C-4), 37.0 (C-1), 36.7 (C-10), 35.7 (C-25), 32.2 (C-15), 32.0 (C-7), 31.6 (C-8), 30.4 (C-23), 30.1 (C-24), 27.6 (C-2), 26.8 (-C*H*_2_-SH), 20.6 (C-11), 19.3 (CH3-19), 18.9 (CH3-21), 16.6 (CH3-26), 16.4 (CH3-18). HRMS (ESI) calcd. for C_29_H_46_O_4_SNa (M+Na)^+^: 513.3015. Found: 513.3035.

3-*O*-[2-(mercapto)acetyl]-(3β, 25 *R*)–furostane-3,26-diol (**21**): Yield 54%, m.p. 122.3 °C, ^1^HNMR (500 MHz, CDCl_3_): δ 4.70 (m, 1H, H-3), 4.27 (m, 1H, H-16), 3.45 (m, 2H, H-27), 3.30 (m, 1H, H-22), 3.20 (d, *J =* 10Hz, 2H, -CO-*CH_2_*-S-), 1.98 and 1.25 (2xm, 2H, H-15), 1.82 and 1.51 (2xm, 2H, H-2), 1.73 and 1.01 (2xm, 2H, H-1), 1.73 (m,1H, H-20), 1.67 and 1.07 (2xm, 2H, H-12), 1.66 and 0.86 (2xm, 2H, H-7), 1.65 (m, 1H, H-25), 1.60 and 1.37 (2xm, 2H, H-4), 1.58 (m, 1H, H-17), 1.57 (m, 2H, H-23), 1.51 (m, 1H, H-8), 1.48 and 1.27 (2xm, 2H, H-11), 1.45 and 1.33 (2xm, 2H, H-24), 1.26 (2xm, 2H, H-6), 1.15 (m, 1H, H-5), 1.06 (m, 1H, H-14), 0.97 (d, *J =* 10Hz, 3H, CH3-21), 0.89 (d, *J =* 5Hz, 3H, CH3-26), 0.82 (s, 3H, CH3-19), 0.76 (s, 3H, CH3-18), 0.63 (m, 1H, H-9); ^13^C NMR (125 MHz, CDCl_3_): δ 170.4 (CO), 90.2 (C-22), 83.2 (C-16), 75.1 (C-3), 67.9 (C-27), 65.1 (C-17), 56.6 (C-14), 54.1 (C-9), 44.6 (C-5), 40.8 (C-13), 40.0 (C-12), 37.9 (C-20), 36.6 (C-1), 35.7 (C-25), 35.5 (C-10), 35.2 (C-8), 33.7 (C-4), 32.1 (C-7), 32.01 (C-15), 30.3 (C-23), 30.0 (C-24), 28.4 (C-6), 27.2 (C-2), 26.8 (-*CH_2_*-S-), 20.8 (C-11), 18.9 (CH3-21), 16.6 (CH3-18), 16.6 (CH3-26), 12.2 (CH3-19). HRMS (ESI) calcd. for C_29_H_48_O_4_SNa (M+Na)^+^: 515.3171. Found: 515.3159.

4′-*O*-[2-(tritylmercapto)acetyl]-genistein (**22**): Compound **1** (100 mg, 0.37 mM) and Tr-S-CH_2_-COOH (248 mg, 0.74 mM) were dissolved in 8 mL THF. Then, DMAP (18 mg, 0.148 mM), and CeCl_3_ (28 mg, 0.074 mM) were added to the solution. To the clear solution was added portionwise of DCC (382 mg, 1.85 mM), and the reaction was stirred for 24h at RT (TLC monitoring: hexane-ethyl acetate). The mixture was treated with 10 mL water, DCM (20 mL) next the organic solution was washed with 0.1 M NaOH, and brine. The organic phase dried over anhydrous magnesium sulphate, filtered and concentrated to the crude solid. The crude product was purified by column chromatography (hexane/ethyl acetate 5:1→1:1) to give **22** (87 mg, 40%) as white solid. Yield 40%, m.p. 162.5 °C; ^1^H NMR (CDCl_3_, 500 MHz): δ 7.82 (s, 1H, H-1), 7.47–7.45 (m, 5H, Ar-H), 7.46–7.44 (m, 2H, H-2′, H-6′), 7.29–7.21 (m, 10H, Ar-H), 7.02–7.01 (m, 2H, H-3′, H-5′), 6.32 (d, 1H, H-8), 6.25 (d, 1H, H-6), 3.19 (brs, 2H, -CH_2_-S-); ^13^C NMR (CDCl_3_, 125 MHz): δ 180.2 (C=O), 168.1 (C=O), 164.1 (C-7), 162.1 (C-5), 158.0 (C-9), 153.1 (C-1), 150.3 (C-4′), 143.8 (C-Ar), 129.8 (C-2′, C-6′), 129.4 (C-Ar), 129.3 (C-Ar), 128.5 (C-1′), 128.0 (C-Ar), 127.9 (C-Ar), 126.9 (C-Ar), 126.7 (C-Ar), 122.8 (C-2), 121.3 (C-3′, C-5′), 105.2 (C-4), 99.3 (C-6), 94.1 (C-8), 67.4 (-S-C-), 34.6 (-CH_2_-); HRMS (ESI) calcd. for C_36_H_26_O_6_SNa (M+H)^+^: 609.1340. Found: 609.1348.

7-*O*-[2-(tritylmercaptomethylcarboxy)ethyl]-genistein (**24**): Yield 80%, m.p. 142.4 °C; ^1^H NMR (CDCl_3_, 500 MHz): δ 12.96 (s, 1H, OH-5), 9.61 (s, 1H, OH-4′), 8.40 (s, 1H, H-1), 7.38–7.37 (m, 2H, H-2′, H-6′), 7.33–7.30 (m, 12H, Ar-H), 7.24–7.23 (m, 3H, Ar-H), 6.83–6.82 (m, 2H, H-3′, H-5′), 6.64 (d, 1H, H-8), 6.38 (d, 1H, H-6), 4.21–4.20 (m, 4H, -CH_2_-CH_2_-), 2.99 (brs, 2H, -CH_2_-); ^13^C NMR (CDCl_3_, 125 MHz): δ 180.4 (C=O), 168.8 (O=C-O), 163.9 (C-7), 161.7 (C-5), 157.5 (C-4′), 157.4 (C-9), 154.4 (C-1), 143.6 (Ar-C), 130.1 (C-2′, C-6′), 129.0 (Ar-C), 128.1 (Ar-C), 126.9 (Ar-C), 122.5 (C-2), 121.0 (C-1′), 115.0 (C-3′, C-5′), 105.5 (C-4), 98.3 (C-6), 92.9 (C-8), 66.6 (-S-C), 66.3 (-O-CH_2_-*C*H_2_-O-), 63.1 (-O-*C*H_2_-CH_2_-O-), 33.8 (-CH_2_-S-); HRMS (ESI) calcd. for C_38_H_30_O_7_S (M+Na)^+^: 653.1635. Found: 653.1610.

4′-*O*-[2-(tritylmercapto)acetyl]-7-O-[2-(tritylmercaptomethylcarboxy)ethyl]-genistein (**25**): ^1^H NMR (DMSO, 500 MHz): δ 12.82 (s, 1H, OH-5), 8.52 (s, 1H, H-1), 7.59–7.57 (m, 2H, H-2′, H-6′), 7.37–7.24 (m, 30H, Ar-H), 7.07–7.05 (m, 2H, H-3′, H-5′), 6.68 (d, 1H, H-8), 6.41 (d, 1H, H-6), 4.22–4.21 (m, 4H, -C*H*_2_-C*H*_2_-), 3.33–3.31 (m, 2H, -CO-CH_2_-), 2.99–2.98 (m, 2H, -CO-CH_2_-); ^13^C NMR (CDCl_3_, 125 MHz): δ 180.0 (C=O),168.8 (C=O), 167.7 (C=O), 164.1 (C-7), 161.7 (C-5), 157.4 (C-9), 155.5 (C-1), 150.0 (C-4′), 143.6 (Ar-C), 130.0 (C-2′, C-6′), 129.1 (Ar-C), 129.0–126.9 (Ar-C), 121.7 (C-2), 121.3 (C-3′, C-5′), 105.5 (C-4), 98.5 (C-6), 93.1 (C-8), 66.9 (-S-*C*-Ar), 66.8 (-S-*C*-Ar), 66.4 (-O-CH_2_-*C*H_2_-CO), 63.0 (-O-*C*H_2_-CH_2_-CO), 34.1 (CO-CH_2_-S-), 33.8 (CO-CH_2_-S-); HRMS (ESI) calcd. for C_59_H_46_O_8_S_2_ (M+Na)^+^: 969.2523. Found: 969.2532.

7-*O*-[2-(mercaptomethylcarboxy)ethyl-genistein (**26**): Yield 81 %, m.p. 168 °C; ^1^H NMR (DMSO, 500 MHz): δ 12.95 (s, 1H, OH-4′), 9.61 (s, 1H, OH-5), 8.41 (s, 1H, H-1), 7.39–7.37 (m, 2H, H-2′, H-6′), 6.82–6.81 (m, 2H, H-3′, H-5′), 6.69–6.68 (m, 1H, H-8), 6.42–6.41 (m, 1H, H-6), 4.41–4.32 (m, 4H, -O-CH_2_-CH_2_-), 3.38–3.33 (m, 2H, -CH_2_-SH); ^13^C NMR (DMSO, 125 MHz): δ 180.4 (C=O), 170.8 (-O-C=O-), 163.9 (C-7), 161.7 (C-5), 157.4 (C-9), 154.4 (C-1), 130.1 (C-2′), 122.5 (C-2), 121.0 (C-1′), 115.1 (C-3′), 105.5 (C-4), 98.4 (C-6), 92.9 (C-8), 66.6 (-O-*C*H_2_-CH_2_-), 63.1 (-O-CH_2_-*C*H_2_-), 25.5 (-CH_2_-SH); HRMS (ESI) calcd. for C_19_H_16_O_7_S (M+Na)^+^: 411.0523. Found: 411.0514.

*N*-(5,11-dimethyl-5H-indolo[2,3-b]quinolin-9-yl)-2-(tritylmercapto)acetamid (**27**): Yield 95%, m.p. 181 °C; ^1^H NMR (CDCl_3_, 500 MHz): δ 8.34–8.33 (d, 1H, *J* = 5 Hz), 8.18–8.16 (dd, 1H, *J* = 5 Hz, *J* = 10 Hz), 8.01–8.00 (m, 2H), 7.75–7.70 (m, 2H), 7.59–7.57 (d, 1H, *J* = 10 Hz), 7.49–7.48 (m, 6H), 7.32–7.26 (m, 6H), 7.22–7.20 (m, 2H), 7.13–7.11 (m, 1H), 4.28 (s, 3H), 3.35 (s, 2H), 3.06 (s, 3H); ^13^C NMR (CDCl_3_, 125 MHz): δ 165.8 (C=O), 162.4, 143.8, 136.6, 130.2, 129.8, 129.4, 128.2, 127.1, 125.7, 124.5, 121.6, 121.2, 121.1, 116.1, 115.6, 114.3, 68.0, 36.7 (CH_2_), 36.4, 32.9, 31.3, 15.4 (CH_3_); HRMS (ESI) calcd. for C_38_H_31_N_3_OS (M+H)^+^: 578.2248. Found: 578.2266.

*N*-(5,11-dimethyl-5H-indolo[2,3-b]quinolin-9-yl)-2-mercaptoacetamid (**28**): Yield 84%, m.p. 228.6 °C; ^1^H NMR (CDCl_3_, 500 MHz): δ 13.7 (brs, 1H), 10.37 (brs, 1H), 8.71–8.70 (m, 1H), 8.60–8.58 (d, 1H, *J* = 10 Hz), 8.33–8.31 (d, 1H, *J* = 10 Hz), 8.13–8.10 (m, 1H), 7.85–7.83 (m, 1H), 7.68–7.66 (dd, 1H, *J* = 5 Hz, *J* = 10 Hz), 7.61–7.59 (d, 1H, *J* = 10 Hz), 4.38 (s, 3H), 3.36–3.34 (m, 2H), 3.23 (s, 3H), 3.04–3.01 (m, 1H); ^13^C NMR (CDCl_3_, 125 MHz): δ 168.6 (C=O), 158.1, 157.9, 148.4, 146.8, 135.3, 134.8, 133.2, 126.8, 125.5, 122.7, 120.7, 120.5, 119.5, 116.9, 114.0, 112.8, 36.2, 28.3 (CH_2_), 15.8; HRMS (ESI) calcd. for C_19_H_17_N_3_OS (M+H)^+^: 336.1176. Found: 336.1171.

2′,3′-*O*-isopropyliden-5′-deoxy-5-fluoro-N^4^-[2-(tritylmercapto)acetyl]cytidine (**30**): Yield 73%; ^1^H NMR (CDCl_3_, 500 MHz): δ 7.59–7.59 (m, 1H, H-6), 7.58–7.20 (m, 15H, Ar-H), 5.64–5.63 (m, 1H, H-11), 5.30 (s, 1H, NH), 4.90–4.89 (m, 1H, H-12), 4.50–4.49 (m, 1H, H-13), 4.33–4.32 (m, 1H, H-14), 3.48 (brs, 2H, -C*H*_2_-S-Tr), 1.57 (s, 3H, CH_3_), 1.40 (d, 3H, H-15), 1.34 (s, 3H, CH_3_); ^13^C NMR (CDCl_3_, 125 MHz): δ 171.1 (CO, -CO-CH_2_-S-Tr), 152.3 (C-4), 143.9, 143.8, 129.4 (C-6), 129.3–128.0 (Ar-C), 114.5 (C-16), 94.3 (C-11), 85.3 (C-12), 84.8 (C-13), 83.8 (C-14), 38.8 (-CH_2_-S-), 35.3, 34.1, 29.6, 29.3, 27.1 (C-18), 25.2 (C-17), 19.1 (C-15); HRMS (ESI) calcd. for C_33_H_32_O_5_SFN_3_ (M+Na)^+^: 624.1929. Found: 624.1944.

1-(5-deoxy-β-d-ribofuranosyl)-5-fluoro-1*H*-spiro[pyrimidine-4,2′-[1,3]thiazolidine]-2,4′-dione (**31**): The intermediate **30** (582 mg, 0.97 mM) was dissolved in 10 mL dichloromethane in argon atmosphere and cooled to 0 °C (ice bath). The colorless solution was treated with 2.0 mL of trifluoroacetic acid, and next, triethylsilane (0.5 mL) was immediately added to the reaction mixture. The reaction was monitored by TLC (hexane:ethyl acetate:methanol 5:3:1), and it was completed after 30 min. Then the reaction was quenched with triethylamine (1 mL), and solution was washed with water (20 mL) and brine (20 mL). The organic phase was evaporated to the oil at room temperature. The crude oil was dissolved in 6 mL methanol, and 1M HCl (3 mL) was added to the solution. The mixture was stirred for 20 min at room temperature. Next, water (10 mL) and ethyl acetate (20 mL) were added. The organic phase was separated, washed with brine, and dried over anhydrous MgSO_4_. After evaporation, the oil was purified by column chromatography (hexane: ethyl acetate: methanol 5:3:1) to afford of **31** as a white solid (247 mg, 80%). m.p. 160.2 °C; ^1^H NMR (CDCl_3_, 500 MHz): δ 9.48 (brs, 1H, NH, H-7), 8.94–8.93 (m, 1H, NH, H-3), 6.76–6.74 (m, 1H, H-6), 5.57–5.56 (dd, 1H, H-11), 5.18–5.04 (m, 1H, OH), 5.00–4.94 (m, 1H, OH), 3.95–3.93 (m, 1H, H-12), 3.71–3.68 (m, 2H, H-14, -C*H*_2_-S-), 3.61–3.57 (m, 2H, H-13, -C*H*_2_-S-), 1.18 (s, 3H, CH_3_); ^13^C NMR (CDCl_3_, 125 MHz): δ 170.0 (CO, -*C*O-CH_2_-S), 148.5 (CO), 139.0 (d, *J* = 232.0 Hz, C-5), 110.2 (C-6), 87.9 (C-11), 78.4 (C-14), 77.2 (C-4), 74.2 (C-13), 71.2 (C-12), 33.8 (C-9), 18.9 (CH_3_, C-15); HRMS (ESI) calcd. for C_11_H_14_O_5_SFN_3_ (M+Na)^+^: 342.0525. Found: 342.0536.

### 3.4. Theoretical Calculations

The density functional theory at the B3LYP/6-31G(d) level was used with the Gaussian G16 suite of programs [49]. The molecular geometries, harmonic frequencies, and the Gibbs free energies were calculated following standard settings within the Gaussian code. For the largest molecule, **M25** (Figure 3, 115 atoms, 1135 basis functions), the numerical frequencies were used in order to overcome extreme demand of the available computer RAM memory for analytically calculated frequencies. In selected cases, the semiempirical quantum mechanical PM7 method [49, also available in 48] was used for preliminary studies of 3D structure of larger complexes.

## 4. Conclusions

In summary, we described here the synthesis of several various biologically active compounds modified with a thiol-linker. This simple two-step procedure provides an advantageous method for the synthesis of thiol analogues useful for potential building theirs conjugates with nanoparticles. Moreover, the above methodology has given access to new sulfur derivatives, which might have potential therapeutic benefits. Further studies, focusing on the building of conjugates of thiol-containing derivatives with gold nanoparticles, will be published elsewhere.

## Supplementary Materials

The Supplementary Materials are available online. Figure S1: The initial step of the **22** deprotection when **22** is surrounded by the Et 3 SiH and four TFA molecules; Figure S2: The initial step of the **24** deprotection when **24** is surrounded by the Et 3 SiH and four; Figure S3: The hypothetical deprotection product of the **22** molecule surrounded by the Et 3 SiOOCCF 3, triphenyl methane and three TFA molecules; Figure S4 The deprotection product **26** of the **24** molecule surrounded by the Et 3 SiOOCCF 3, triphenyl methane and three TFA molecules TFA molecules; 1 H and 13 C NMR spectra of compounds; The Cartesian coordinates, in Angstroms.
