# Supplementary material for: Synthesis of Thiol Derivatives of Biological Active Compounds for Nanotechnology Application
Source: Molecules. 2020 Jul 30;25(15):3470. doi: 10.3390/molecules25153470 (PMC7435828; doi:10.3390/molecules25153470)
Supplement: Supplementary file 1 [file molecules-25-03470-s001.pdf]

Supplementary Materials

Synthesis of thiol derivatives of biological active compounds for nanotechnology application

Katarzyna Sidoryk[1]*, Olga Michalak[1], Marek Kubiszewski[2], Andrzej Leś[3], Marcin Cybulski[1], Elżbieta Stolarczyk[2], Jan Doubsky[4]

[1] Department of Biomedical Technology, Cosmetic Chemicals and Electrochemistry, Team of Chemistry, Łukasiewicz Research Network – Industrial Chemistry Institute, 8 Rydygiera Str., 01-793 Warsaw, Poland.

[2] Analytical Department, Łukasiewicz Research Network – Industrial Chemistry Institute, 8 Rydygiera Str, 01-793 Warsaw, Poland.

[3] Faculty of Chemistry, University of Warsaw, 1 Pasteur Str. 02-093 Warsaw, Poland

[4] Zentiva Group, a.s., 529/16 U Kabelovny Str., 10200 Prague 10, Czech Republic

List of contents:

1. Theoretical studies.

2. ^{1}H and ^{13}C NMR spectra of compounds:

1. diosgenin 2-(tritylmercapto)acetate **(10)**
2. tigogenin 2-(tritylmercapto)acetate **(11)**
3. flumethasone 2-(tritylmercapto)acetate **(12)**
4. 11-*O*-[2-(tritylmercapto)acetyl]-fluticasone propionate **(13)**
5. 3-*O*-[2-(tritylmercapto)acetyl]-Uursolic acid methyl ester **(14)**
6. β-sitosterol 2-(tritylmercapto)acetate **(15)**
7. flumethasone 2-mercaptoacetate **(16)**
8. 11-*O*-[2-(mercapto)acetyl] -fluticasone propionate **(17)**
9. 3-*O*-[2-(mercapto)acetyl]-ursolic acid methyl ester **(18)**
10. β-sitosterol 2-mercaptoacetate **(19)**
11. 3-*O*-[2-(mercapto)acetyl]-(3β, 25*R*)–furost–5–ene-3,26-diol **(20)**
12. 3-*O*-[2-(mercapto)acetyl]-(3β, 25*R*)–furostane-3,26-diol **(21)**
13. 4’-*O*-[2-(tritylmercapto)acetyl]-genistein **(22)**
14. 7-*O*-[2-(tritylmercaptomethylcarboxy)ethyl]-genistein **(24)**
15. 4’-*O*-[2-(tritylmercapto)acetyl]-7-*O*-[2-(tritylmercaptomethylcarboxy)ethyl]-genistein **(25)**
16. 7-*O*-[2-(mercaptomethylcarboxy)ethyl-genistein **(26)**
17. *N*-(5,11-dimethyl-5*H*-indolo[2,3-b]quinolin-9-yl)-2-(tritylmercapto)acetamid **(27)**
18. *N*-(5,11-dimethyl-5*H*-indolo[2,3-b]quinolin-9-yl)-2-mercaptoacetamid **(28)**
19. 2’,3’-*O*-isopropyliden-5’-deoxy-5-fluoro-*N*^{4}-[2-(tritylmercapto)acetyl]cytidine **(30)**
20. 1-(5-deoxy-β-D-ribofuranosyl)-5-fluoro-1*H*-spiro[pyrimidine-4,2’-[1,3]thiazolidine]-2,4’-dione **(31)**

1. Theoretical studies.

Fig. S1 The initial step of the **22** deprotection when **22** is surrounded by the Et_3SiH and four TFA molecules.

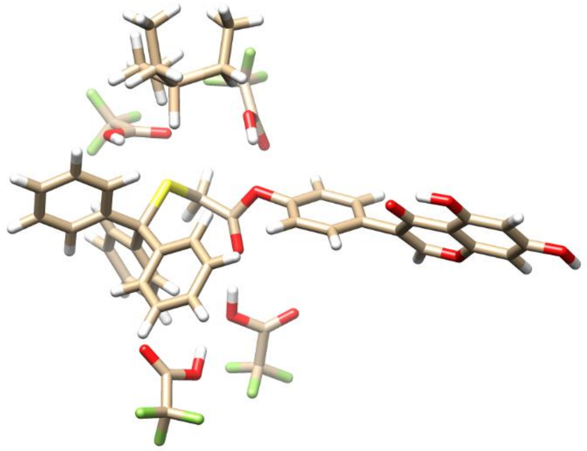

The **22** molecule is seen in the center, two TFA molecules sit below **22**, and another two TFA molecules sit above **22**. At the top there is also the Et_3SiH silane. The TFA molecule which is supposed to be involved in the bonding with Et_3SiH and releasing hydrogen atom towards the sulfur atom is seen at the top-left of the figure. The Cartesian coordinates of this complex are attached, in Angstroms, as the Figure S1.xyz file. They can be used for 3D visualization with the use of molecular graphics software such as, for example, JMol, RasMol, etc. The software used here for molecular graphics: UCSF Chimera 1.14, http://rbvi.ucsf.edu.

Fig. S2 The initial step of the **24** deprotection when **24** is surrounded by the Et_3SiH and four TFA molecules.

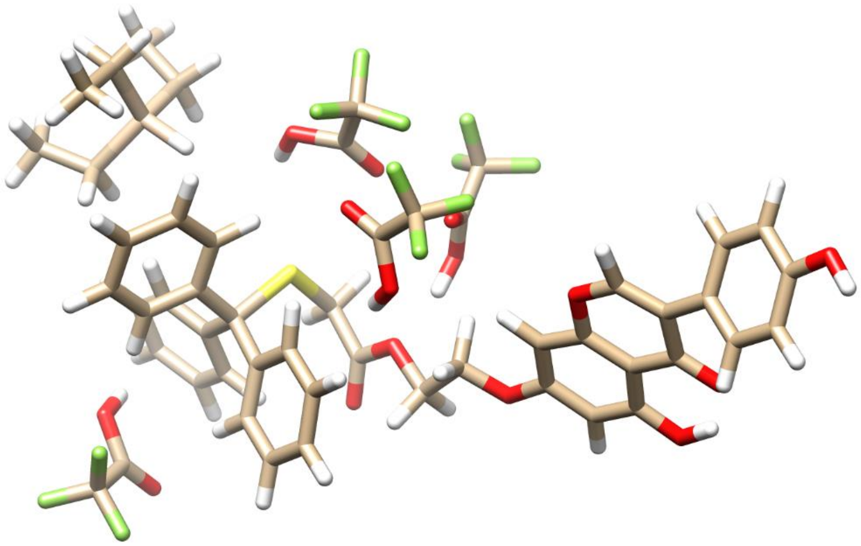

The **24** molecule is seen in the center, three TFA molecules sit above **24** and the 4th TFA molecule is below **24** molecule. At the top-left there is the Et_3SiH silane. The Cartesian coordinates are attached, in Angstroms, as the Figure S2.xyz file.

Fig. S3 The hypothetical deprotection product of the **22** molecule surrounded by the $Et_3SiOOCCF_3$, triphenyl methane and three TFA molecules.

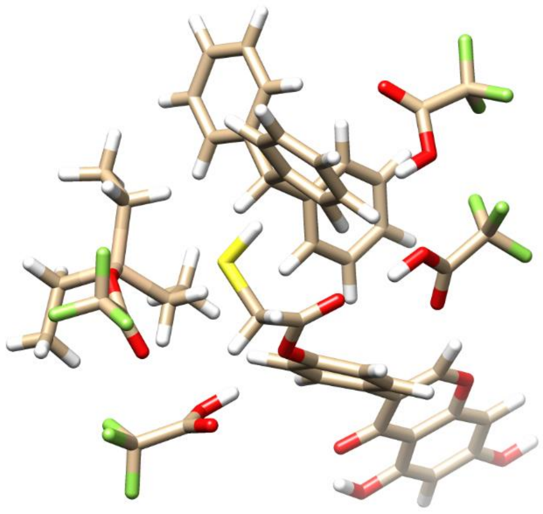

The **22’** molecule is seen in the down-right corner. Two TFA molecules sit above **22’**, and the third TFA molecule sits at the down-left corner. The $Et_3SiOOCCF_3$ molecule sits in the middle-left side. The triphenyl methane sit at the middle-top side. The Cartesian coordinates are attached, in Angstroms, as the Figure S3.xyz file.

Fig. S4 The deprotection product **26** of the **24** molecule surrounded by the $Et_3SiOOCCF_3$, triphenyl methane and three TFA molecules.

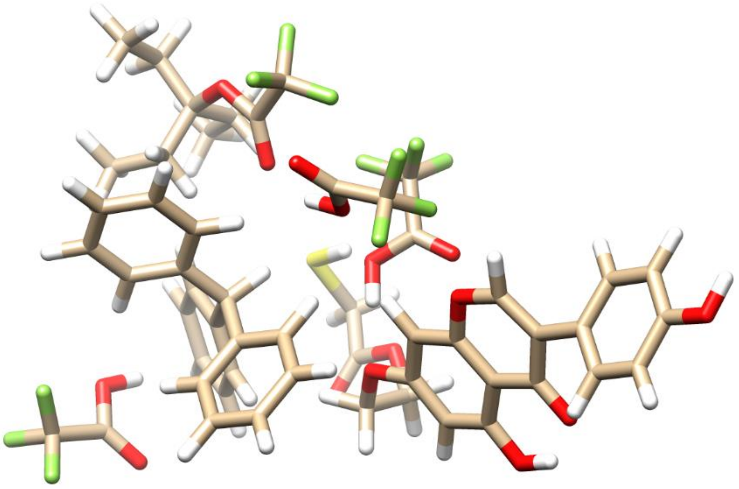

The **26** molecule is seen in the down-right corner. Two TFA molecules sit above **26**, and the third TFA molecule sits at the down-left corner. The $Et_3SiOOCCF_3$ molecule sits at the top-left side. The triphenyl methane sit at the middle-left side. The Cartesian coordinates are attached, in Angstroms, as the Figure S4.xyz file.

The Cartesian coordinates, in Angstroms.

Figure S1.xyz

```
124

C -2.745394 -1.168877 1.327378
C -3.127765 -1.995809 0.1309
C -2.249659 -2.945694 -0.394805
C -2.591687 -3.668975 -1.534831
C -3.806861 -3.436903 -2.171363
C -4.683708 -2.481173 -1.660259
C -4.347137 -1.764481 -0.517486
H -5.047059 -1.022049 -0.129321
H -5.63639 -2.298115 -2.15416
H -4.071959 -4.001468 -3.06336
H -1.906569 -4.418286 -1.930007
H -1.28977 -3.134348 0.090877
C -3.845534 -0.964163 2.344221
C -4.933879 -1.836773 2.405501
C -5.926887 -1.659124 3.365928
C -5.835496 -0.612665 4.279958
C -4.731413 0.237007 4.248715
C -3.730434 0.051618 3.29928
H -2.845625 0.689136 3.335057
H -4.642398 1.04266 4.976191
H -6.616128 -0.469174 5.023649
H -6.770881 -2.346079 3.402111
H -5.008716 -2.67737 1.714299
C -1.577134 -1.733286 2.102419
C -0.392189 -1.040052 2.342583
C 0.625822 -1.632847 3.09268
C 0.463798 -2.914015 3.612477
C -0.72933 -3.603856 3.393347
C -1.743044 -3.015559 2.646159
H -2.675641 -3.560337 2.478298
H -0.874017 -4.602546 3.805543
H 1.261864 -3.376368 4.190068
H 1.547985 -1.084233 3.278867
H -0.230703 -0.027728 1.968903
S -2.429852 0.532297 0.620375
C -1.580218 0.25885 -0.967279
C -0.146033 -0.036535 -0.73273
O 0.40613 -1.106633 -0.697975
O 0.549187 1.120175 -0.537406
C 1.935657 1.047887 -0.530277
C 2.584875 1.589358 -1.636067
C 3.974724 1.63077 -1.608742
C 4.675201 1.131221 -0.503164
C 3.985944 0.586414 0.588821
C 2.59538 0.532824 0.578399
H 2.036826 0.09451 1.403586
H 4.539849 0.207818 1.45288
C 6.133451 1.18538 -0.487611
C 6.877109 0.737658 -1.518536
H 6.534412 0.264047 -2.438279
O 8.233341 0.796798 -1.584121
C 8.908499 1.379525 -0.544657
C 10.285353 1.449054 -0.711958
C 11.017237 2.041461 0.331411
C 10.422667 2.54183 1.491633
C 9.03287 2.441946 1.613665
C 8.243422 1.861553 0.589772
C 6.799006 1.760898 0.692076
O 6.188874 2.1275 1.682138
```

O 8.495179 2.921706 2.732273
H 7.477845 2.809615 2.740136
H 11.01931 2.995086 2.279649
O 12.356823 2.162075 0.267922
H 12.735088 1.795476 -0.571098
H 10.76277 1.065559 -1.603274
H 4.518145 2.061233 -2.451507
H 2.018188 1.965937 -2.489427
H -2.051304 -0.554398 -1.568636
H -1.721754 1.179011 -1.584976
C -6.148611 1.88335 -2.967215
C -4.9859 1.868487 -1.951163
O -5.486384 1.548401 -0.759152
H -4.80388 1.541394 -0.008273
O -3.844534 2.108241 -2.214694
F -7.123002 2.709745 -2.611145
F -5.751282 2.267512 -4.17277
F -6.70426 0.686877 -3.11829
C -1.784571 4.64381 -3.13136
C -0.900845 3.48341 -2.633234
O -0.719701 3.617716 -1.316996
H -0.190152 2.861026 -0.913101
O -0.450121 2.611602 -3.316651
F -1.620733 5.765767 -2.442146
F -1.526065 4.947383 -4.397643
F -3.077468 4.356224 -3.071689
C 1.587913 -5.219838 -3.116018
C 1.587867 -3.768699 -2.579652
O 0.466213 -3.598797 -1.866292
H 0.385735 -2.676954 -1.440639
O 2.435918 -2.956889 -2.777013
F 0.416264 -5.583441 -3.621982
F 2.476348 -5.388371 -4.085384
F 1.887475 -6.109283 -2.178025
C 0.081621 -7.288449 1.656318
C -0.491139 -6.051179 0.928371
O 0.240155 -5.87236 -0.170616
H 0.050362 -5.025513 -0.674553
O -1.410787 -5.393583 1.309187
F 1.385371 -7.182518 1.879561
F -0.48155 -7.474456 2.843988
F -0.097108 -8.41598 0.981977
Si -2.501864 4.262183 2.750881
C -1.138716 5.225201 1.891802
C -2.705902 4.730058 4.563019
C -4.146646 4.444999 1.862715
C -3.277032 6.127348 4.836341
C -1.37831 6.729286 1.719181
H -0.17804 5.071984 2.442591
H -0.979888 4.770339 0.879415
H -1.711249 4.64319 5.065928
H -3.35712 3.97048 5.059785
H -4.555113 5.454036 2.113932
H -4.879598 3.717169 2.280413
H -4.292057 6.261202 4.441083
H -2.655597 6.940491 4.429817
H -3.339238 6.303686 5.922196
H -2.289425 6.957419 1.149172
H -0.545965 7.179806 1.156497
H -1.436367 7.269144 2.673566
C -4.094383 4.302706 0.338636
H -2.094994 2.861766 2.736435
H -3.633921 3.364428 -0.000929
H -3.534525 5.121173 -0.141998
H -5.112238 4.336368 -0.080422

Figure S2.xyz

```
131

H 14.242439 -0.221469 -3.053839
O 13.651207 -0.975984 -2.817371
C 12.448388 -0.558444 -2.343815
C 11.571582 -1.578382 -1.957898
C 10.322229 -1.220706 -1.46988
C 9.953518 0.128621 -1.375682
C 10.855155 1.128857 -1.756873
C 12.112186 0.793275 -2.249598
H 12.813236 1.565074 -2.545218
H 10.574615 2.179559 -1.660629
C 8.632313 0.488962 -0.874777
C 7.51563 -0.115037 -1.326039
H 7.431544 -0.894831 -2.082026
O 6.252074 0.156474 -0.903119
C 6.085139 1.112295 0.059072
C 4.764671 1.333911 0.432773
C 4.537073 2.312615 1.409286
C 5.572699 3.037839 2.008357
C 6.882603 2.77836 1.599051
C 7.168946 1.808592 0.605365
C 8.521419 1.538287 0.150589
O 9.48284 2.149739 0.587578
O 7.844024 3.491016 2.183487
H 8.769768 3.243373 1.819282
H 5.37004 3.7868 2.769469
O 3.310433 2.655788 1.866882
C 2.202975 1.9968 1.243422
C 0.97439 2.626868 1.897949
O 0.042334 1.537501 2.008847
C -1.174578 1.853778 2.5584
C -2.033787 0.647466 2.694201
S -2.463907 -0.159992 1.123718
C -3.445007 1.049823 0.083217
C -4.622157 1.383037 0.957365
C -4.571801 2.379027 1.936215
C -5.670252 2.633596 2.755309
C -6.835056 1.883382 2.620694
C -6.88972 0.869599 1.665488
C -5.793224 0.621026 0.846348
H -5.853434 -0.184669 0.109737
H -7.789958 0.264037 1.562866
H -7.690767 2.078183 3.264283
H -5.609084 3.425889 3.502942
H -3.673599 2.984447 2.07905
C -3.853129 0.356758 -1.20528
C -4.846077 0.979325 -1.97388
C -5.22939 0.448404 -3.199402
C -4.607729 -0.701479 -3.686367
C -3.607732 -1.311637 -2.937127
C -3.235941 -0.793106 -1.694803
H -2.450937 -1.319236 -1.147423
H -3.103468 -2.201366 -3.315721
H -4.901737 -1.114595 -4.648845
H -6.008313 0.93882 -3.781553
H -5.318291 1.892266 -1.612697
C -2.524008 2.15948 -0.362299
C -1.347803 1.800343 -1.037762
C -0.430664 2.764532 -1.436488
C -0.695624 4.11446 -1.205803
C -1.897664 4.484913 -0.612547
C -2.813439 3.512735 -0.207158
H -3.759167 3.838561 0.222781
H -2.133044 5.538924 -0.460382
```

H 0.023613 4.87282 -1.509771
H 0.486589 2.461628 -1.942755
H -1.165501 0.754135 -1.294999
H -2.949827 0.907063 3.276551
H -1.514963 -0.145872 3.29383
O -1.417006 2.976662 2.901289
H 0.512695 3.418716 1.278614
H 1.176813 3.029327 2.910068
H 2.287479 0.906674 1.442302
H 2.235865 2.189497 0.153814
H 3.965666 0.758186 -0.021475
H 9.628713 -1.996112 -1.146017
H 11.87762 -2.617862 -2.038225
Si -5.693324 -3.811694 -0.961048
C -7.033257 -2.493884 -0.944382
C -5.605684 -4.756503 0.663026
C -5.87391 -4.995376 -2.40097
C -6.784873 -5.688556 0.959968
C -8.467901 -2.99028 -1.152689
H -6.979094 -1.941889 0.024523
H -6.793887 -1.742643 -1.738644
H -5.504569 -4.023454 1.498756
H -4.658808 -5.352658 0.670271
H -6.83703 -5.548566 -2.274876
H -5.071497 -5.770285 -2.334466
H -6.912146 -6.479593 0.208306
H -7.743388 -5.158642 1.046568
H -6.6283 -6.201785 1.920501
H -8.613308 -3.491398 -2.119936
H -9.166453 -2.140644 -1.13782
H -8.804739 -3.683061 -0.36984
C -5.83904 -4.356841 -3.794012
H -4.418037 -3.109427 -1.066796
H -4.885701 -3.856459 -4.01372
H -6.642127 -3.623922 -3.952975
H -5.968376 -5.129889 -4.56601
C 2.172669 -1.63433 -1.568873
C 0.90609 -1.122651 -0.84949
O 1.09801 0.15894 -0.534748
H 0.414848 0.534518 0.104482
O -0.086838 -1.757541 -0.651345
F 3.124898 -2.005649 -0.725731
F 1.913596 -2.685593 -2.336861
F 2.716114 -0.722446 -2.367605
C -0.352791 -4.234028 0.070464
C -1.149636 -3.438234 1.126647
O -2.449042 -3.609202 0.8865
H -3.045216 -3.09946 1.512012
O -0.679961 -2.824817 2.043177
F 0.930676 -3.901163 0.062266
F -0.393481 -5.539756 0.318796
F -0.80588 -4.082029 -1.164737
C 1.533791 -3.027867 3.362991
C 1.449611 -1.728345 2.537031
O 0.99735 -0.760007 3.334077
H 0.791564 0.102959 2.86007
O 1.766318 -1.603953 1.389195
F 0.548899 -3.15466 4.241495
F 1.492074 -4.11055 2.597539
F 2.663541 -3.107908 4.057142
C -6.073972 5.393016 -1.762829
C -6.131966 4.802219 -0.337813
O -6.656376 3.577293 -0.428008
H -6.710445 3.086725 0.450053
O -5.766755 5.34631 0.659655
F -5.301069 6.470577 -1.818391
F -5.591572 4.542476 -2.659482
F -7.269943 5.764514 -2.203342

Figure S3.xyz

```
124

C -0.788025 2.480418 -1.41201
C -1.147147 3.088296 -0.079471
C -0.423989 2.732599 1.061166
C -0.724913 3.298571 2.297471
C -1.769709 4.213951 2.407261
C -2.525792 4.536542 1.280724
C -2.225198 3.967907 0.045416
C -1.051259 3.385455 -2.590551
C -0.882184 4.768892 -2.507211
C -1.108521 5.569202 -3.623654
C -1.497378 4.992136 -4.831384
C -1.651498 3.610286 -4.921066
C -1.427383 2.807774 -3.804909
C 0.6624 2.052556 -1.45106
C 0.987229 0.71165 -1.23532
C 2.319165 0.306413 -1.261014
C 3.331696 1.230162 -1.509079
C 3.008223 2.567216 -1.730108
C 1.678595 2.978298 -1.691872
S -3.504623 0.900281 1.581968
C -2.746936 -0.204 2.817485
C -1.292838 -0.328076 2.534449
O -0.411134 0.428507 2.846258
O -1.056451 -1.474928 1.836812
C 0.227923 -1.841945 1.484721
C 1.291381 -1.76627 2.373938
C 2.532498 -2.224019 1.938563
C 2.686784 -2.732424 0.643145
C 1.577469 -2.849788 -0.205274
C 0.330748 -2.394865 0.208286
C 4.016993 -3.113756 0.182902
C 5.081684 -2.313624 0.40182
O 6.357536 -2.599461 0.039395
C 6.606382 -3.799202 -0.571404
C 7.942161 -4.037906 -0.86598
C 8.239548 -5.257757 -1.497096
C 7.266605 -6.200995 -1.830676
C 5.935973 -5.911842 -1.51391
C 5.576496 -4.700737 -0.86937
C 4.202012 -4.389092 -0.521155
O 3.27785 -5.136591 -0.801397
O 5.02956 -6.827779 -1.847354
O 9.504065 -5.590594 -1.821706
C -6.844597 -0.615453 1.164022
C -5.642537 -1.216736 0.378316
O -5.551213 -0.596863 -0.76158
O -4.970008 -2.106363 0.819817
F -7.990344 -1.196342 0.828968
F -6.710278 -0.777868 2.473625
F -7.014227 0.683835 0.962288
C -4.848723 -4.646336 1.347629
C -3.677805 -3.821614 1.917136
O -2.740179 -3.691785 0.972343
O -3.622102 -3.376241 3.026151
F -4.949522 -4.588479 0.026595
F -4.753004 -5.936931 1.651122
F -6.017979 -4.239834 1.825291
C 4.319621 2.499799 2.226283
C 3.25765 1.376006 2.224943
O 2.099039 1.879581 2.640327
O 3.475111 0.24385 1.909134
F 4.048808 3.500464 3.049599
F 5.505201 2.024078 2.596246
```

F 4.496425 3.02245 1.020376
C 3.673872 6.149792 0.544781
C 2.299376 5.442163 0.572658
O 2.410694 4.369986 1.357262
O 1.324768 5.8094 -0.011192
F 4.599694 5.447838 -0.09709
F 3.610508 7.325656 -0.068364
F 4.15615 6.386796 1.757448
Si -4.5918 -0.674879 -2.099376
C -2.914647 -1.382096 -1.650091
C -4.503071 1.128215 -2.608582
C -5.495082 -1.698864 -3.378314
C -4.444827 1.421379 -4.110286
C -1.867389 -1.424432 -2.765228
C -5.742396 -3.16016 -2.991153
H -2.827659 4.222126 -0.827748
H -3.350688 5.243432 1.364102
H -1.992527 4.67524 3.366461
H -0.149892 3.017357 3.181312
H 0.370767 1.991242 0.975323
H -1.53665 1.727601 -3.884157
H -1.942866 3.155925 -5.865485
H -1.673991 5.618538 -5.702408
H -0.975151 6.647395 -3.550347
H -0.565706 5.229802 -1.568064
H 1.433281 4.027206 -1.863939
H 3.794544 3.291044 -1.938417
H 4.370197 0.908597 -1.534165
H 2.572621 -0.738294 -1.083258
H 0.20431 -0.020896 -1.041919
H -0.543244 -2.474732 -0.437984
H 1.692169 -3.29933 -1.194889
H 5.101542 -1.333437 0.886932
H 4.083307 -6.538997 -1.579727
H 7.532175 -7.134445 -2.321679
H 10.162636 -4.894811 -1.562781
H 8.711676 -3.318757 -0.618533
H 3.388395 -2.178943 2.613594
H 1.168726 -1.368663 3.377532
H -2.90875 0.150552 3.857772
H -3.28802 -1.186958 2.765998
H -2.035198 -3.026551 1.224998
H 1.329133 1.22438 2.691236
H 1.549531 3.854945 1.470962
H -2.510273 -0.802371 -0.782445
H -3.089475 -2.420319 -1.256504
H -3.627667 1.608917 -2.104116
H -5.408803 1.628571 -2.17164
H -4.934976 -1.660598 -4.344951
H -6.47927 -1.202236 -3.578456
H -5.366921 1.145641 -4.639341
H -3.609161 0.927263 -4.624025
H -4.306695 2.502726 -4.274145
H -2.18086 -2.02376 -3.631518
H -0.938256 -1.889575 -2.400571
H -1.588026 -0.428733 -3.137249
H -6.369474 -3.269722 -2.093987
H -4.81807 -3.729762 -2.817904
H -6.276003 -3.680825 -3.80056
H -3.077596 2.094575 2.029253
H -1.43901 1.560601 -1.517179

Figure S4.xyz

```
131

O -12.346412 0.450025 -1.663324
C -11.037818 0.244352 -1.364015
C -10.229003 1.246492 -0.825781
C -8.896933 0.945293 -0.557894
C -8.388608 -0.328893 -0.828681
C -9.227891 -1.321331 -1.357844
C -10.558876 -1.042624 -1.633713
C -6.985905 -0.623972 -0.561912
C -6.004019 0.207608 -0.965671
O -4.678233 0.028233 -0.733781
C -4.294897 -1.063323 -0.004381
C -2.931581 -1.15783 0.233759
C -2.487905 -2.258581 0.983948
C -3.347377 -3.247123 1.455239
C -4.71649 -3.11234 1.181715
C -5.22014 -2.012357 0.449125
C -6.637246 -1.847745 0.17241
O -7.460536 -2.676717 0.52549
O -5.513469 -4.069832 1.649721
O -1.144867 -2.240531 1.173061
C -0.5512 -3.216268 2.021886
C -0.847313 -2.911778 3.482355
O -0.00482 -1.872072 3.967338
C 1.285654 -2.201963 4.260958
C 1.963418 -1.047617 4.916582
S 2.507889 0.178033 3.676411
C 2.328762 -0.835036 -0.485804
C 3.228252 -1.505472 0.518763
C 2.939327 -2.777149 1.017672
C 3.737431 -3.348934 2.007312
C 4.849286 -2.663599 2.493096
C 5.147445 -1.395088 1.995903
C 4.337309 -0.818673 1.023124
C 3.036972 -0.085586 -1.591143
C 4.305551 -0.44537 -2.041872
C 4.884831 0.234876 -3.111981
C 4.199879 1.274928 -3.736054
C 2.926038 1.630041 -3.293726
C 2.345672 0.950573 -2.227513
C 1.400709 -1.786983 -1.202224
C 0.036759 -1.50545 -1.250341
C -0.822706 -2.346559 -1.954692
C -0.323517 -3.474353 -2.601244
C 1.042236 -3.753481 -2.558338
C 1.903961 -2.906056 -1.869173
O 1.733112 -3.293114 4.027116
C 4.757533 2.561365 -0.369234
C 4.222821 3.665533 1.897714
C 5.506841 4.99015 0.119611
C 5.431482 3.173461 2.685742
C 6.219151 2.125168 -0.404275
C 5.554525 5.38112 -1.357217
C -2.741868 2.273064 -1.475476
C -1.2042 2.38233 -1.447786
O -0.750136 1.809158 -0.329149
O -0.533408 2.905417 -2.285358
F -3.288553 2.073437 -0.283799
F -3.302634 3.376526 -1.956472
F -3.157137 1.281895 -2.256091
C 1.102297 4.839439 -0.794459
C 2.141842 3.835896 -0.217925
O 3.284186 4.476368 -0.145476
O 1.873254 2.700511 0.079013
```

F -0.131609 4.557106 -0.394365
F 1.328219 6.090191 -0.415086
F 1.08698 4.844482 -2.118962
C -1.023141 2.707132 2.252392
C -1.484137 1.247204 2.049045
O -0.411661 0.470596 1.858015
O -2.623418 0.897519 2.088549
F 0.273051 2.919336 2.061246
F -1.656582 3.541026 1.437815
F -1.275183 3.130016 3.486511
C 5.724501 -4.661142 -2.718993
C 5.098518 -4.500996 -1.315397
O 5.663228 -3.431373 -0.750405
O 4.264639 -5.206537 -0.837836
F 5.151433 -5.638087 -3.409657
F 5.613737 -3.56535 -3.458675
F 7.019286 -4.947682 -2.666416
H -12.642123 1.365895 -1.44242
H -11.226091 -1.796995 -2.043023
H -8.831378 -2.321722 -1.544253
H -6.10206 1.134662 -1.530376
H -6.496373 -3.892462 1.407895
H -3.002646 -4.10986 2.012701
H 4.563972 0.184404 0.657931
H 6.006471 -0.848446 2.379364
H 5.467346 -3.10838 3.268407
H 3.477841 -4.328647 2.411439
H 2.085264 -3.339883 0.637182
H 1.339268 1.213283 -1.905002
H 2.375893 2.430296 -3.788353
H 4.65122 1.801383 -4.573521
H 5.872128 -0.058017 -3.465079
H 4.845976 -1.263252 -1.568565
H 2.972275 -3.107973 -1.851252
H 1.436807 -4.632003 -3.066226
H -0.997315 -4.134327 -3.142571
H -1.888131 -2.121876 -1.993284
H -0.356059 -0.628216 -0.738366
H 2.871904 -1.400879 5.454977
H 1.311582 -0.574055 5.68056
H -0.740562 -3.809266 4.12032
H -1.854919 -2.46674 3.630807
H 0.526795 -3.081331 1.773187
H -0.856765 -4.232304 1.716933
H -2.237672 -0.400454 -0.118096
H -8.251371 1.707211 -0.121495
H -10.626967 2.232116 -0.615999
H 4.131929 1.744909 0.061568
H 4.388222 2.667309 -1.41597
H 3.384629 2.947846 2.030852
H 3.859181 4.622275 2.327267
H 6.509806 4.67428 0.46737
H 5.242288 5.880275 0.72795
H 6.304849 3.820643 2.551993
H 5.713129 2.15268 2.395452
H 5.207737 3.145872 3.761475
H 6.843977 2.830432 -0.965297
H 6.314024 1.149224 -0.903962
H 6.648191 2.024225 0.597892
H 4.626395 5.875224 -1.675073
H 5.695428 4.510676 -2.009276
H 6.375444 6.080456 -1.554938
H 0.256378 1.912017 -0.204398
H -0.644085 -0.499854 1.690126
H 5.3033 -3.210757 0.167478
C 4.508579 3.855452 0.400389
H 1.311588 0.548045 3.18828
H 1.717442 -0.090587 0.112717

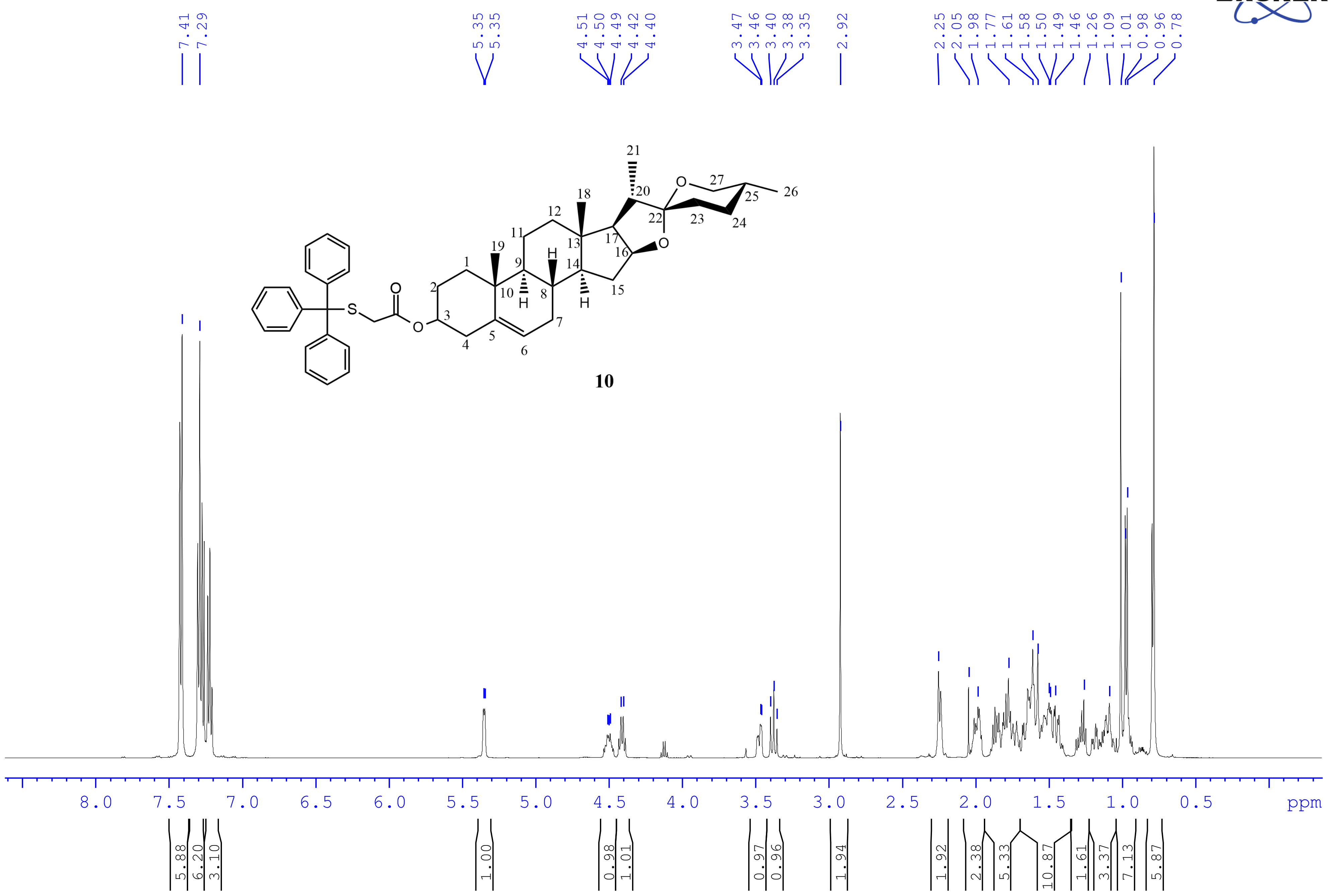
7.41
7.29
5.35
5.35
4.51
4.50
4.49
4.42
4.40
3.47
3.46
3.40
3.38
3.35
2.92
2.25
2.05
1.98
1.77
1.61
1.58
1.50
1.49
1.46
1.26
1.09
1.01
0.98
0.96
0.78
8.0 7.5 7.0 6.5 6.0 5.5 5.0 4.5 4.0 3.5 3.0 2.5 2.0 1.5 1.0 0.5 ppm
5.88
6.20
3.10
1.00
0.98
1.01
0.97
0.96
1.94
1.92
2.38
5.33
10.87
1.61
3.37
7.13
5.87
10

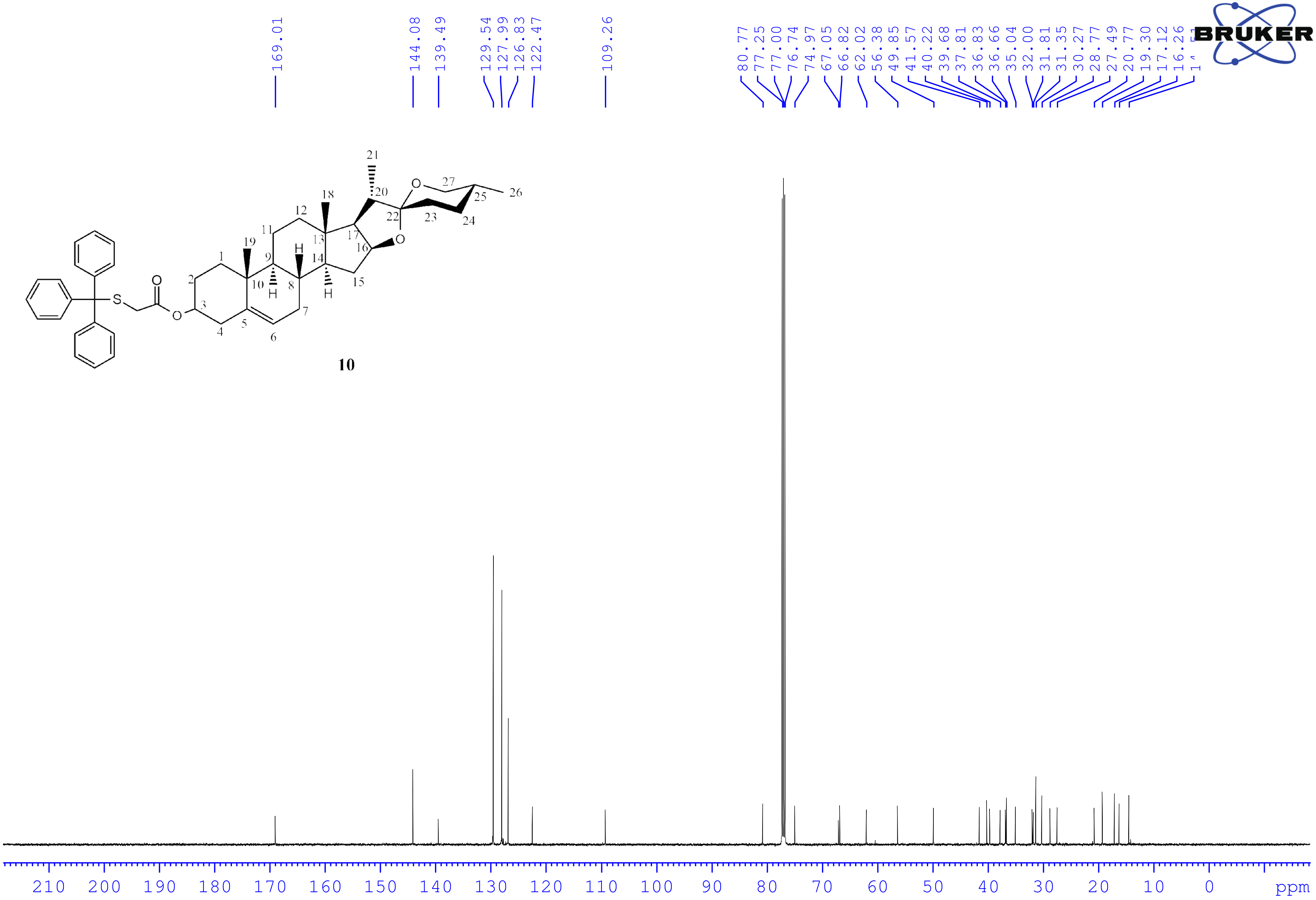
169.01
144.08
139.49
129.54
127.99
126.83
122.47
109.26
80.77
77.25
77.00
76.74
74.97
67.05
66.82
62.02
56.38
49.85
41.57
40.22
39.68
37.81
36.83
36.66
35.04
32.00
31.81
31.35
30.27
28.77
27.49
20.77
19.30
17.12
16.26
BRUKER
10
210 200 190 180 170 160 150 140 130 120 110 100 90 80 70 60 50 40 30 20 10 0 ppm

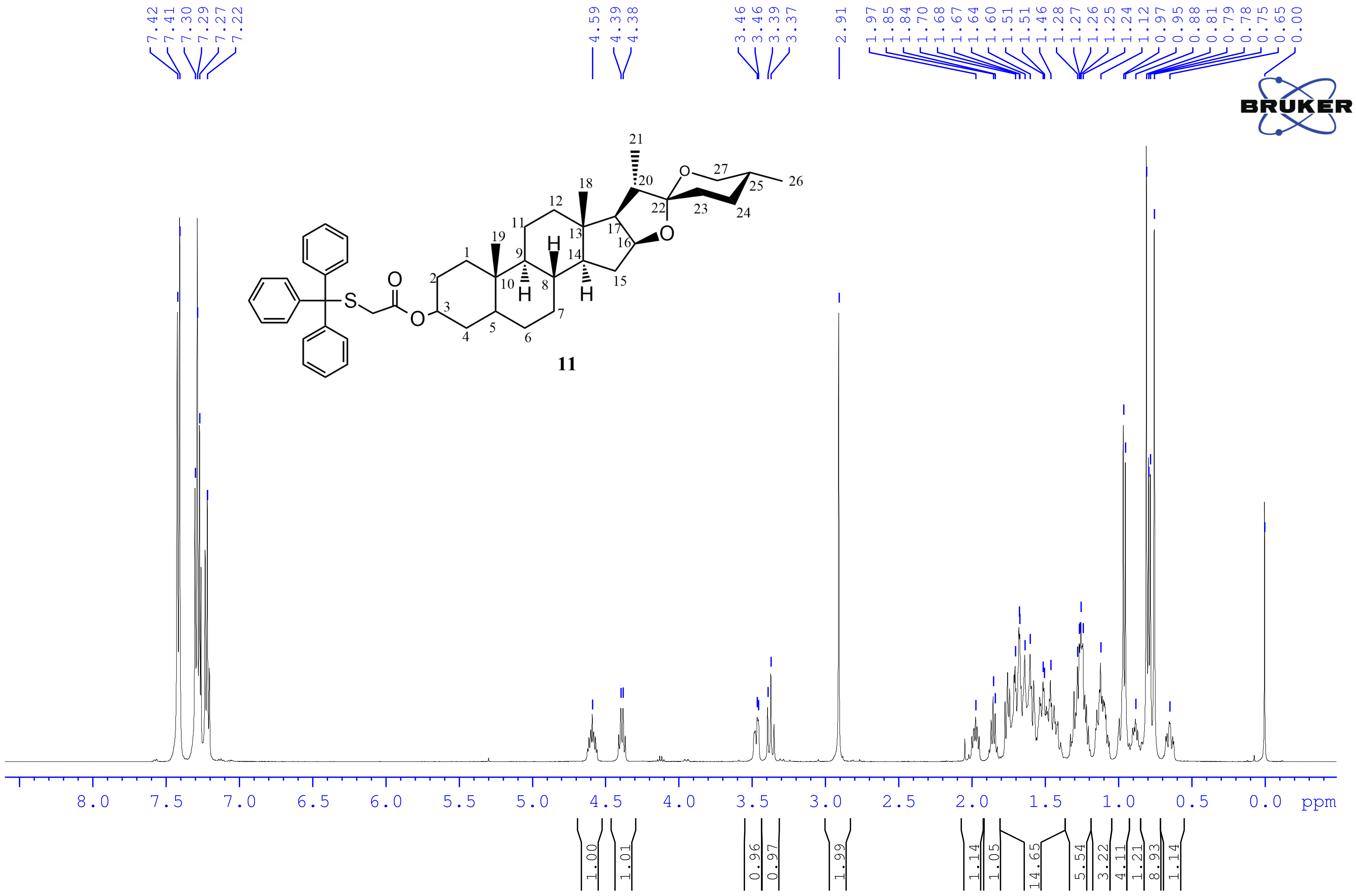

7.42
7.41
7.30
7.29
7.27
7.22
4.59
4.39
4.38
3.46
3.46
3.39
3.37
2.91
1.97
1.85
1.84
1.70
1.68
1.67
1.64
1.60
1.51
1.51
1.46
1.28
1.27
1.26
1.25
1.24
1.12
0.97
0.95
0.88
0.81
0.79
0.78
0.75
0.65
0.00
BRUKER
11
8.0
7.5
7.0
6.5
6.0
5.5
5.0
4.5
4.0
3.5
3.0
2.5
2.0
1.5
1.0
0.5
0.0
ppm
1.00
1.01
0.96
0.97
1.99
1.14
1.05
14.65
5.54
3.22
4.11
1.21
8.93
1.14

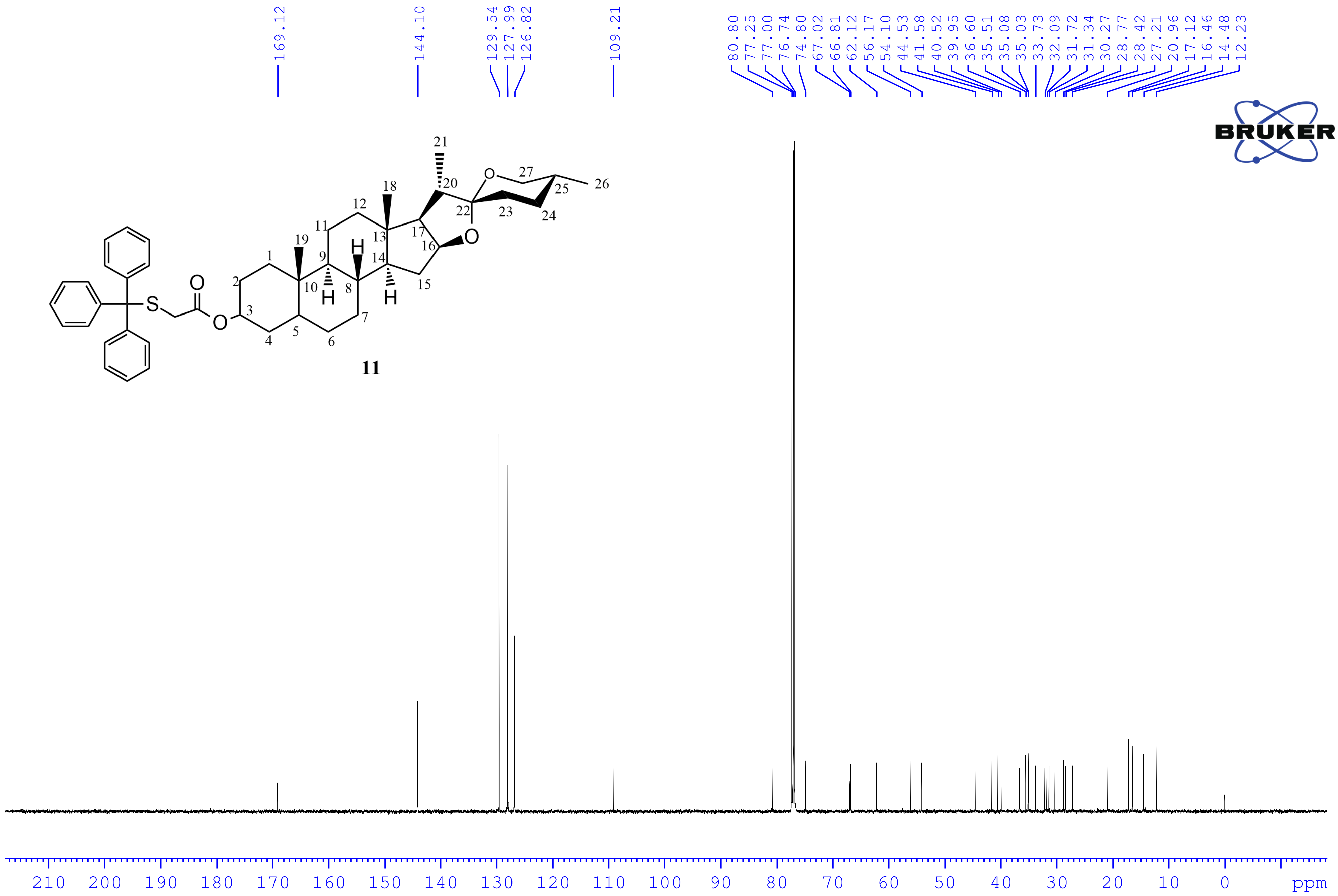
169.12
144.10
129.54
127.99
126.82
109.21
80.80
77.25
77.00
76.74
74.80
67.02
66.81
62.12
56.17
54.10
44.53
41.58
40.52
39.95
36.60
35.51
35.08
35.03
33.73
32.09
31.72
31.34
30.27
28.77
28.42
27.21
20.96
17.12
16.46
14.48
12.23
11
210 200 190 180 170 160 150 140 130 120 110 100 90 80 70 60 50 40 30 20 10 0 ppm

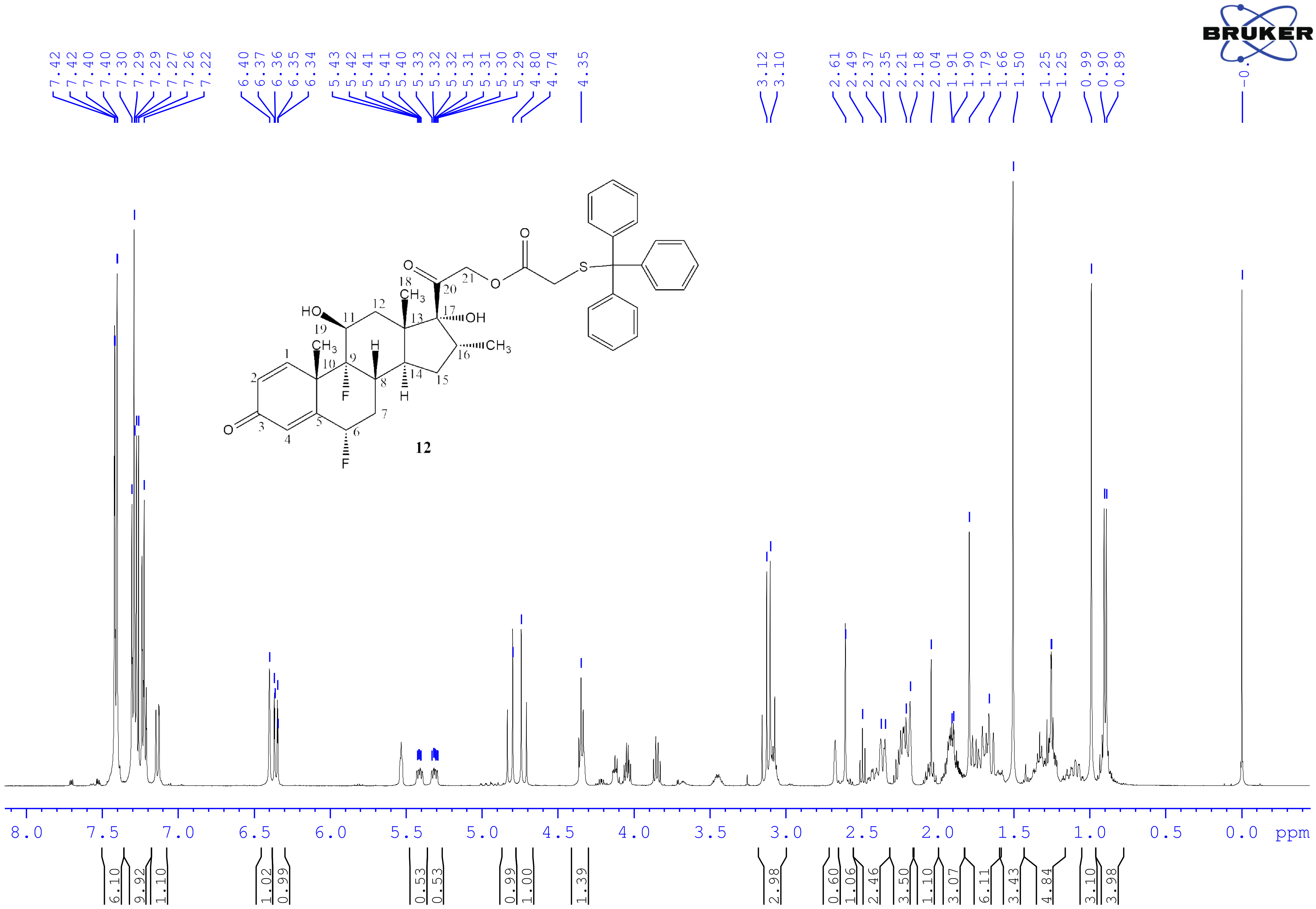
BRUKER
7.42 7.42 7.40 7.40 7.30 7.29 7.29 7.27 7.26 7.22
6.40 6.37 6.36 6.35 6.34
5.43 5.42 5.41 5.41 5.40 5.33 5.32 5.32 5.31 5.31 5.30 5.29 4.80 4.74
4.35
3.12 3.10
2.61 2.49 2.37 2.35 2.21 2.18 2.04 1.91 1.90 1.79 1.66 1.50
1.25 1.25 0.99 0.90 0.89
-0.
HO
OH
CH3
F
H
O
S
12
8.0 7.5 7.0 6.5 6.0 5.5 5.0 4.5 4.0 3.5 3.0 2.5 2.0 1.5 1.0 0.5 0.0 ppm
6.10 9.92 1.10 1.02 0.99 0.53 0.53 0.99 1.00 1.39 2.98 0.60 1.06 2.46 3.50 1.10 3.07 6.11 3.43 4.84 3.10 3.98

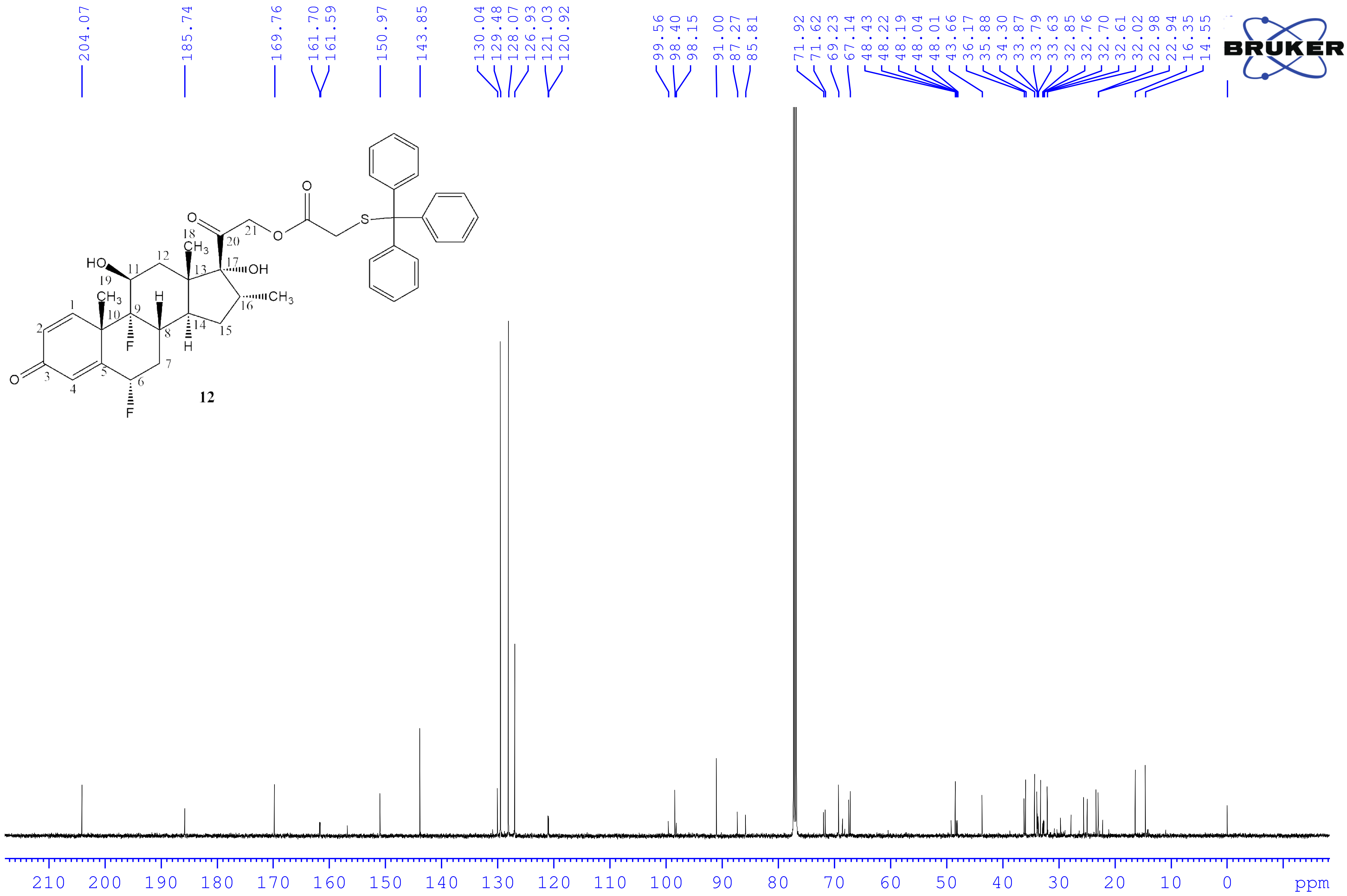
204.07
185.74
169.76
161.70
161.59
150.97
143.85
130.04
129.48
128.07
126.93
121.03
120.92
99.56
98.40
98.15
91.00
87.27
85.81
71.92
71.62
69.23
67.14
48.43
48.22
48.19
48.04
48.01
43.66
36.17
35.88
34.30
33.87
33.79
33.63
32.85
32.76
32.70
32.61
32.02
22.98
22.94
16.35
14.55
HO
O
OH
CH3
S
F
H
12
210 200 190 180 170 160 150 140 130 120 110 100 90 80 70 60 50 40 30 20 10 0 ppm

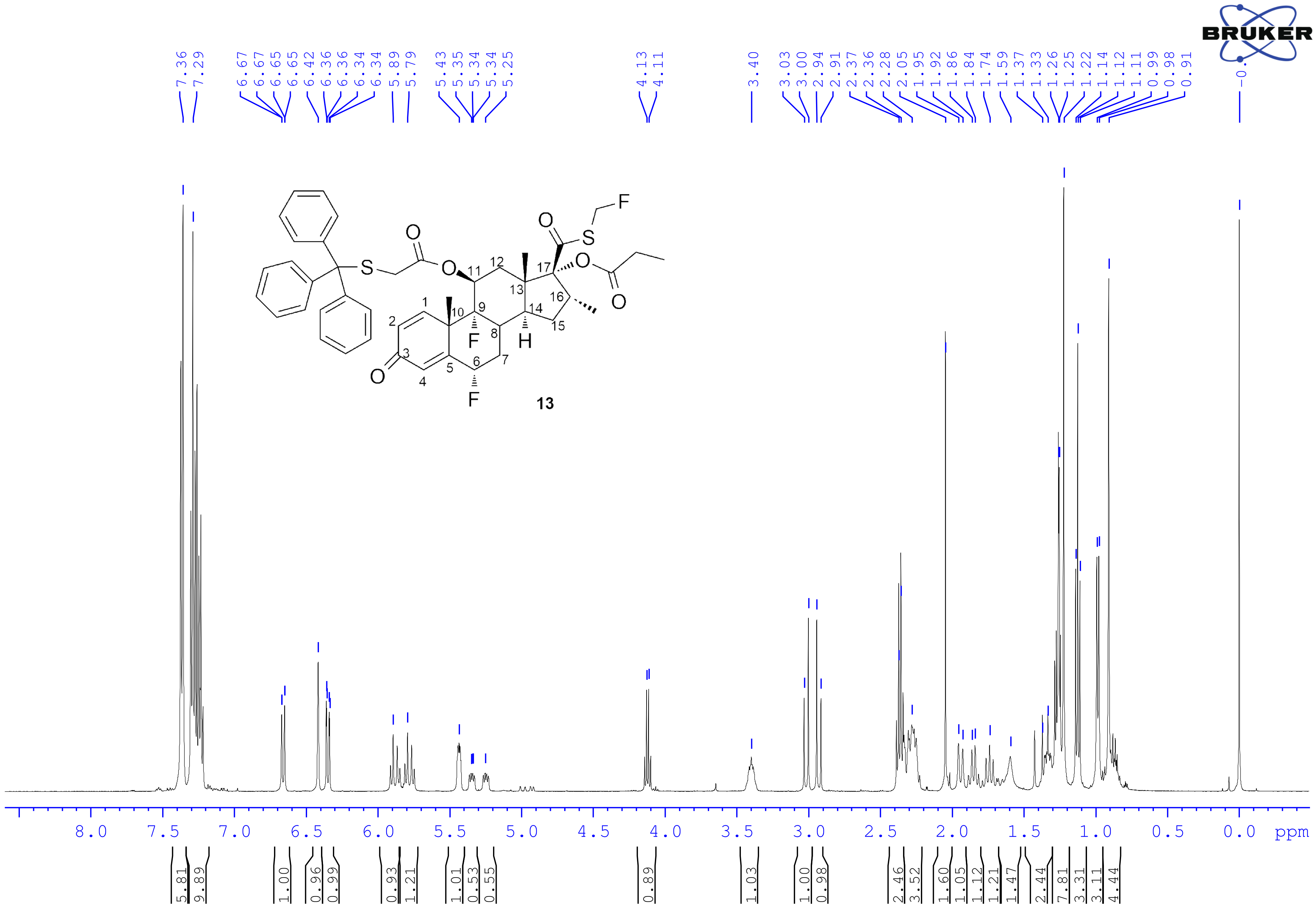

7.36
7.29
6.67
6.67
6.65
6.65
6.42
6.36
6.36
6.34
6.34
5.89
5.79
5.43
5.35
5.34
5.34
5.25
4.13
4.11
3.40
3.03
3.00
2.94
2.91
2.37
2.36
2.28
2.05
1.95
1.92
1.86
1.84
1.74
1.59
1.37
1.33
1.26
1.25
1.22
1.14
1.12
1.11
0.99
0.98
0.91
-0.
8.0 7.5 7.0 6.5 6.0 5.5 5.0 4.5 4.0 3.5 3.0 2.5 2.0 1.5 1.0 0.5 0.0 ppm
5.81
9.89
1.00
0.96
0.99
0.93
1.21
1.01
0.53
0.55
0.89
1.03
1.00
0.98
2.46
3.52
1.60
1.05
1.12
1.21
1.47
2.44
7.81
3.31
3.11
4.44
13

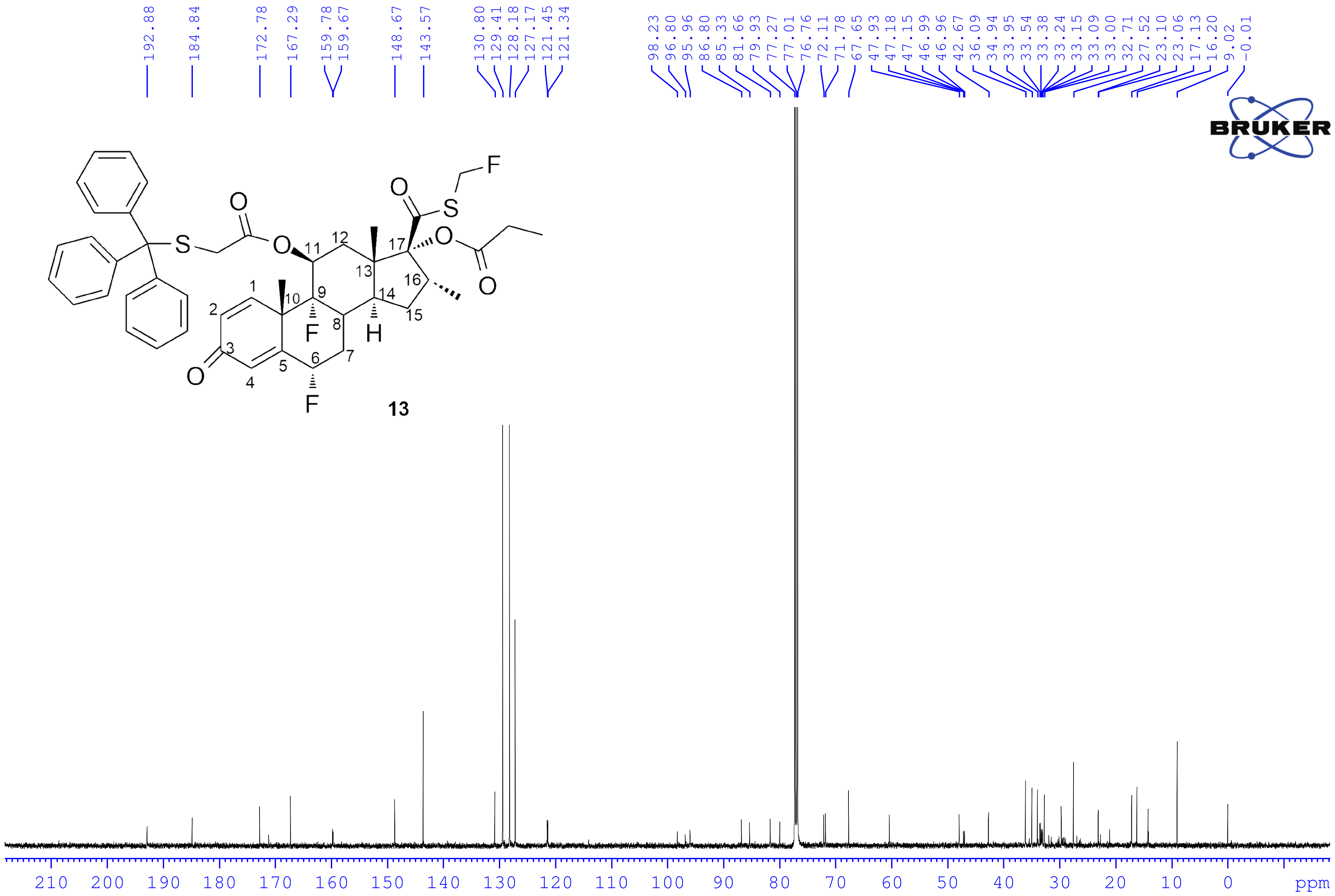
192.88
184.84
172.78
167.29
159.78
159.67
148.67
143.57
130.80
129.41
128.18
127.17
121.45
121.34
98.23
96.80
95.96
86.80
85.33
81.66
79.93
77.27
77.01
76.76
72.11
71.78
67.65
47.93
47.18
47.15
46.99
46.96
42.67
36.09
34.94
33.95
33.54
33.38
33.24
33.15
33.09
33.00
32.71
27.52
23.10
23.06
17.13
16.20
9.02
-0.01
BRUKER
F
O
S
O
O
S
O
11
12
17
O
13
16
1
10
9
14
2
15
F
8
H
3
6
7
O
5
4
F
210
200
190
180
170
160
150
140
130
120
110
100
90
80
70
60
50
40
30
20
10
0
ppm

13

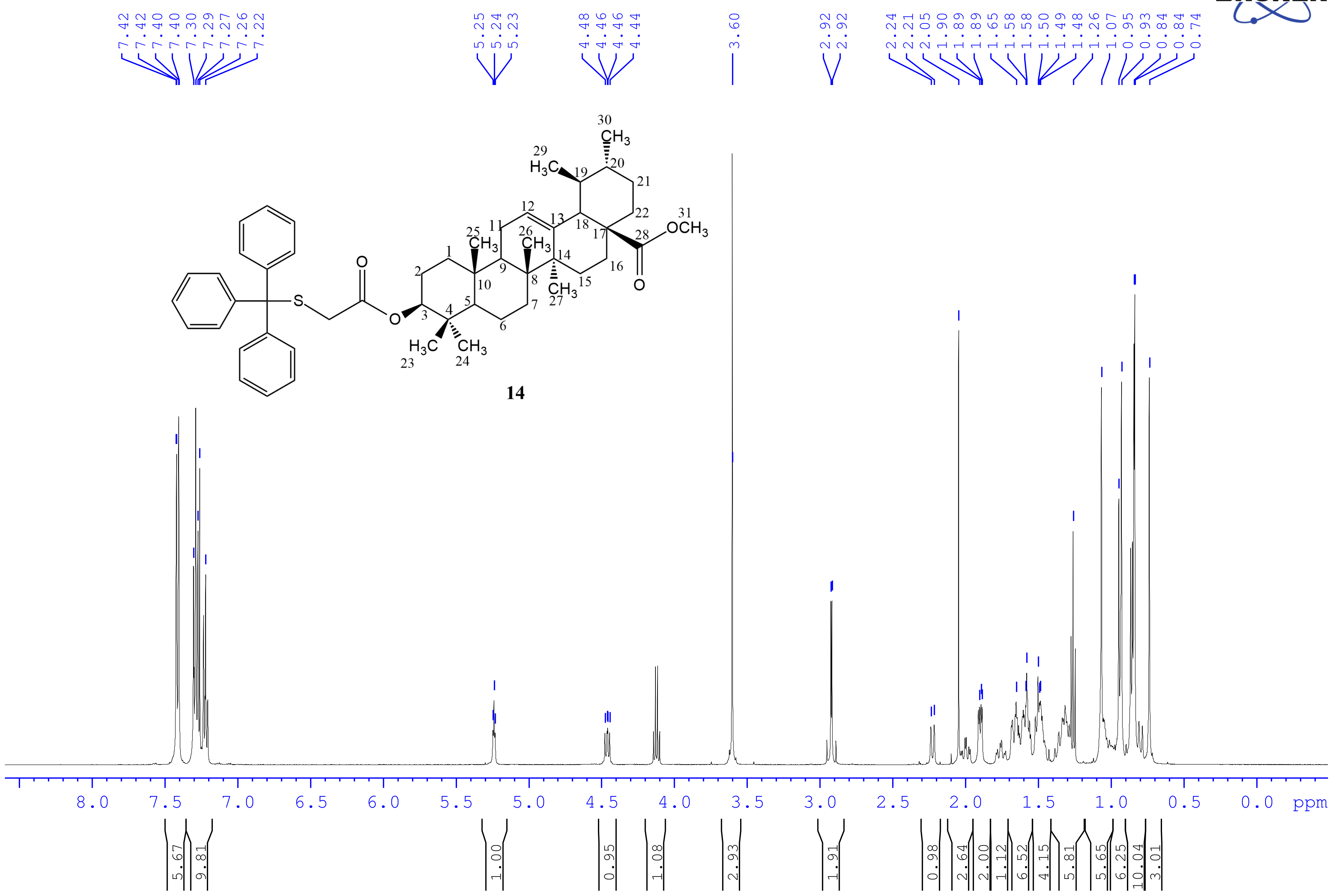

7.42 7.42 7.40 7.40 7.30 7.29 7.27 7.26 7.22
5.25 5.24 5.23
4.48 4.46 4.46 4.44
3.60
2.92 2.92
2.24 2.21 2.05 1.90 1.89 1.89 1.65 1.58 1.58 1.50 1.49 1.48 1.26 1.07 0.95 0.93 0.84 0.84 0.74
14
8.0 7.5 7.0 6.5 6.0 5.5 5.0 4.5 4.0 3.5 3.0 2.5 2.0 1.5 1.0 0.5 0.0 ppm
5.67 9.81 1.00 0.95 1.08 2.93 1.91 0.98 2.64 2.00 1.12 6.52 4.15 5.81 5.65 6.25 10.04 3.01

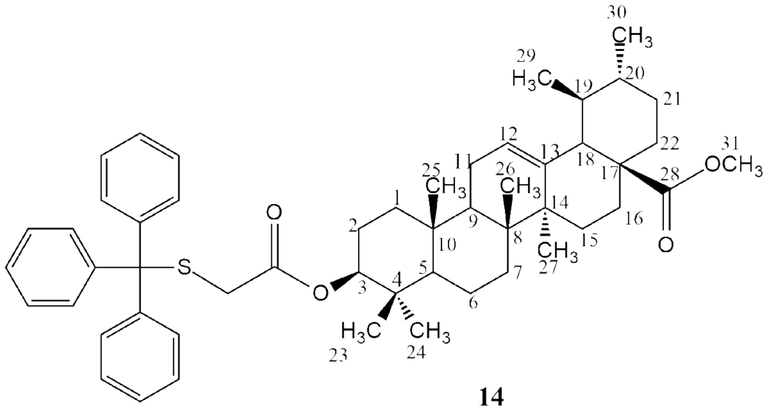

14

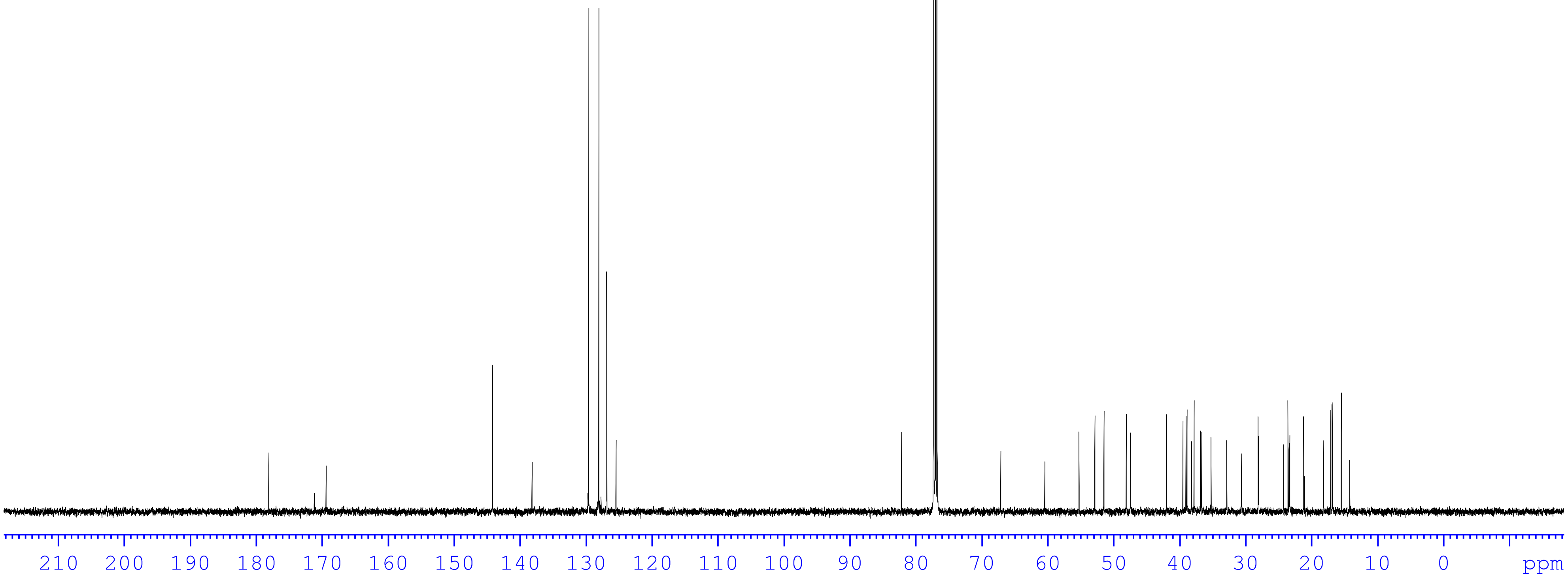
178.06
169.35
144.11
138.15
129.54
128.01
126.83
125.40
82.13
77.25
77.00
76.74
67.08
55.21
52.82
51.44
48.05
47.41
41.94
39.45
39.00
38.83
38.17
37.76
36.79
36.61
35.20
32.83
30.61
28.06
27.97
24.18
23.54
23.36
23.26
21.17
BRUKER
210 200 190 180 170 160 150 140 130 120 110 100 90 80 70 60 50 40 30 20 10 0 ppm

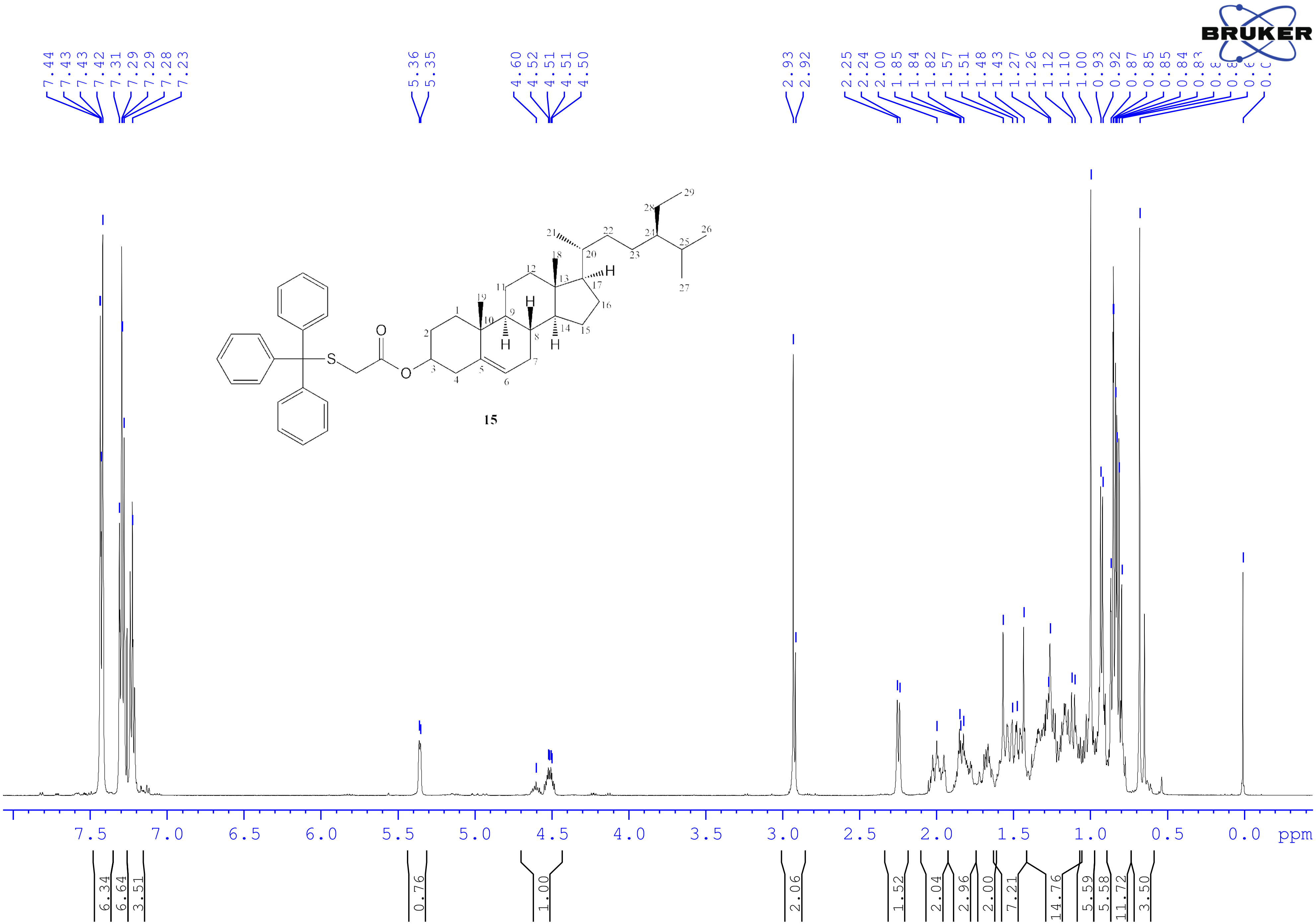
7.44
7.43
7.43
7.42
7.31
7.29
7.29
7.28
7.23
5.36
5.35
4.60
4.52
4.51
4.51
4.50
2.93
2.92
2.25
2.24
2.00
1.85
1.84
1.82
1.57
1.51
1.48
1.43
1.27
1.26
1.12
1.10
1.00
0.93
0.92
0.87
0.85
0.85
0.84
BRUKER
15
7.5
7.0
6.5
6.0
5.5
5.0
4.5
4.0
3.5
3.0
2.5
2.0
1.5
1.0
0.5
0.0
ppm
6.34
6.64
3.51
0.76
1.00
2.06
1.52
2.04
2.96
2.00
7.21
14.76
5.59
5.58
11.72
3.50

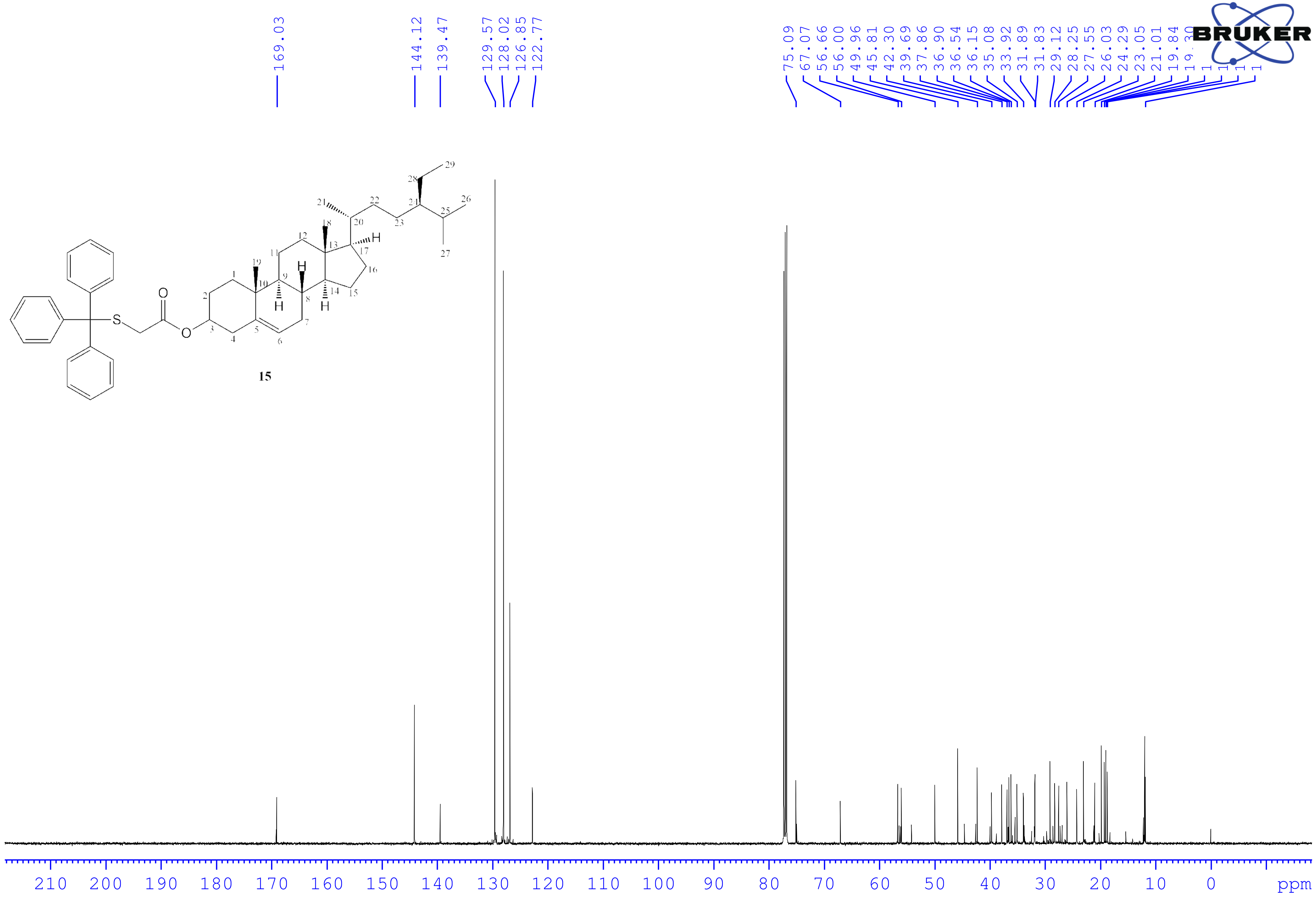
169.03
144.12
139.47
129.57
128.02
126.85
122.77
75.09
67.07
56.66
56.00
49.96
45.81
42.30
39.69
37.86
36.90
36.54
36.15
35.08
33.92
31.89
31.83
29.12
28.25
27.55
26.03
24.29
23.05
21.01
19.84
19.30
BRUKER
15
210 200 190 180 170 160 150 140 130 120 110 100 90 80 70 60 50 40 30 20 10 0 ppm

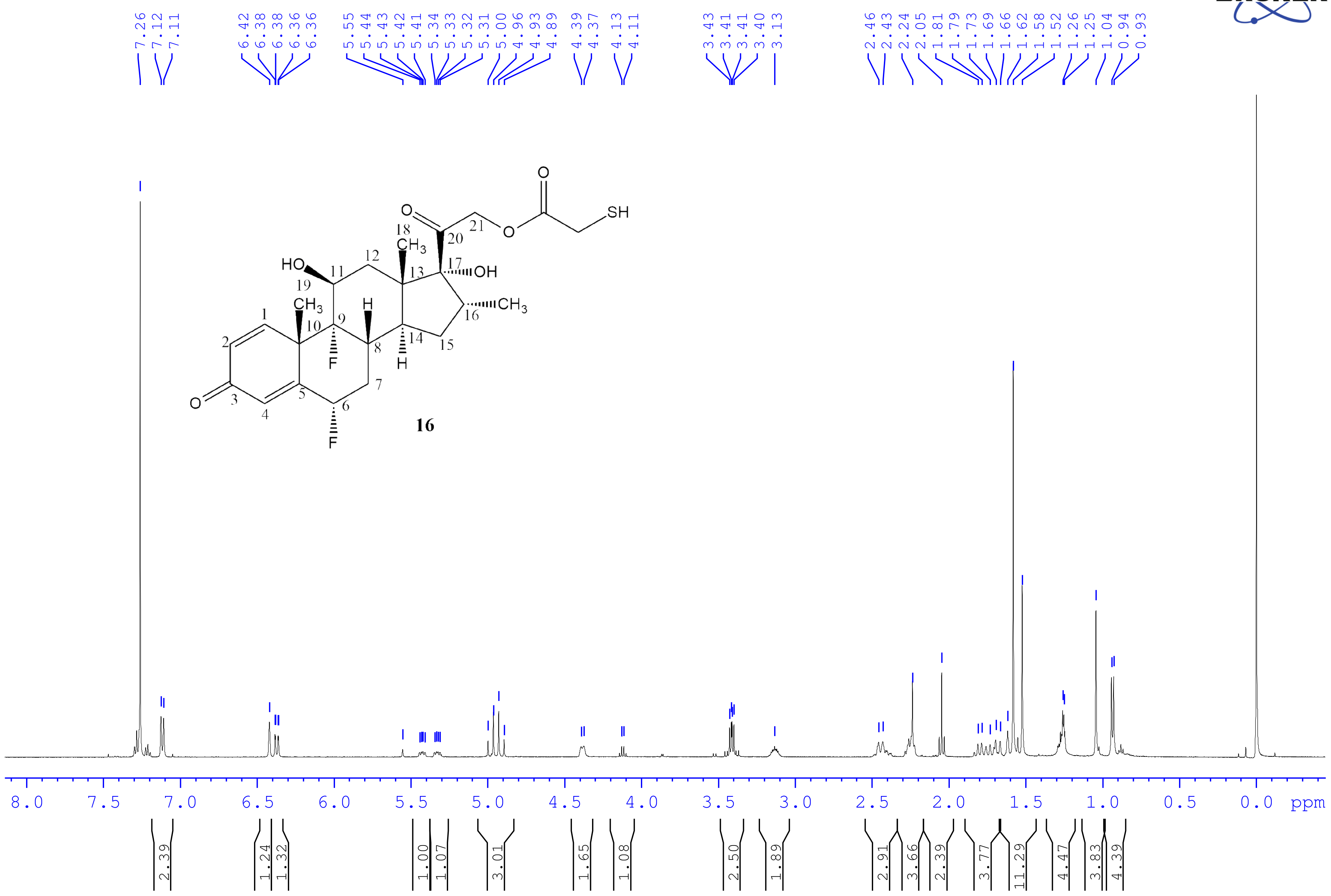

7.26 7.12 7.11
6.42 6.38 6.38 6.36 6.36
5.55 5.44 5.43 5.42 5.41 5.34 5.33 5.32 5.31 5.00 4.96 4.93 4.89
4.39 4.37 4.13 4.11
3.43 3.41 3.41 3.40 3.13
2.46 2.43 2.24 2.05 1.81 1.79 1.73 1.69 1.66 1.62 1.58 1.52 1.26 1.25 1.04 0.94 0.93
O
SH
O
O
HO
OH
CH3
CH3
CH3
H
H
F
F
O
16
8.0 7.5 7.0 6.5 6.0 5.5 5.0 4.5 4.0 3.5 3.0 2.5 2.0 1.5 1.0 0.5 0.0 ppm
2.39 1.24 1.32 1.00 1.07 3.01 1.65 1.08 2.50 1.89 2.91 3.66 2.39 3.77 11.29 4.47 3.83 4.39

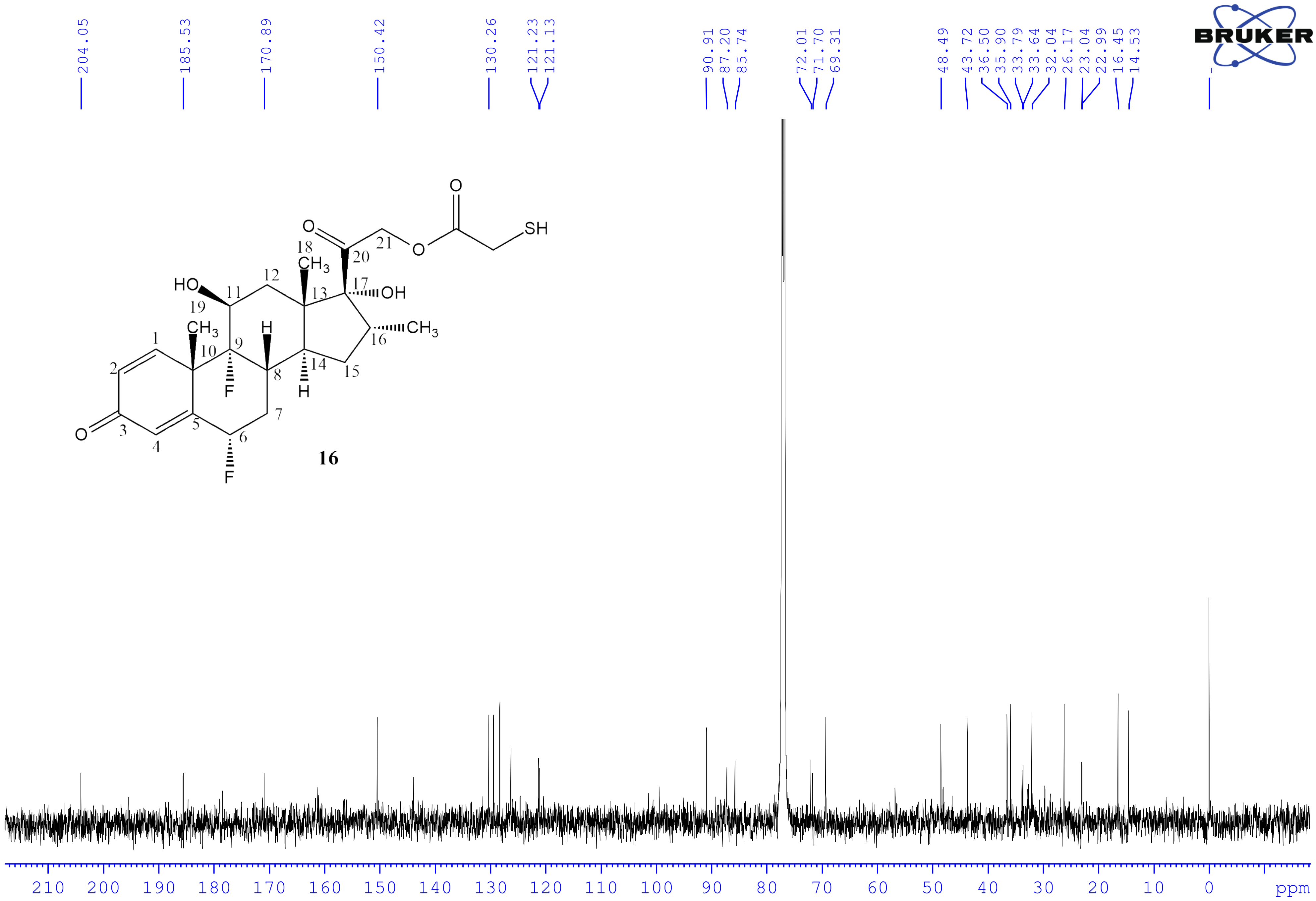
204.05
185.53
170.89
150.42
130.26
121.23
121.13
90.91
87.20
85.74
72.01
71.70
69.31
48.49
43.72
36.50
35.90
33.79
33.64
32.04
26.17
23.04
22.99
16.45
14.53
16
210 200 190 180 170 160 150 140 130 120 110 100 90 80 70 60 50 40 30 20 10 0 ppm

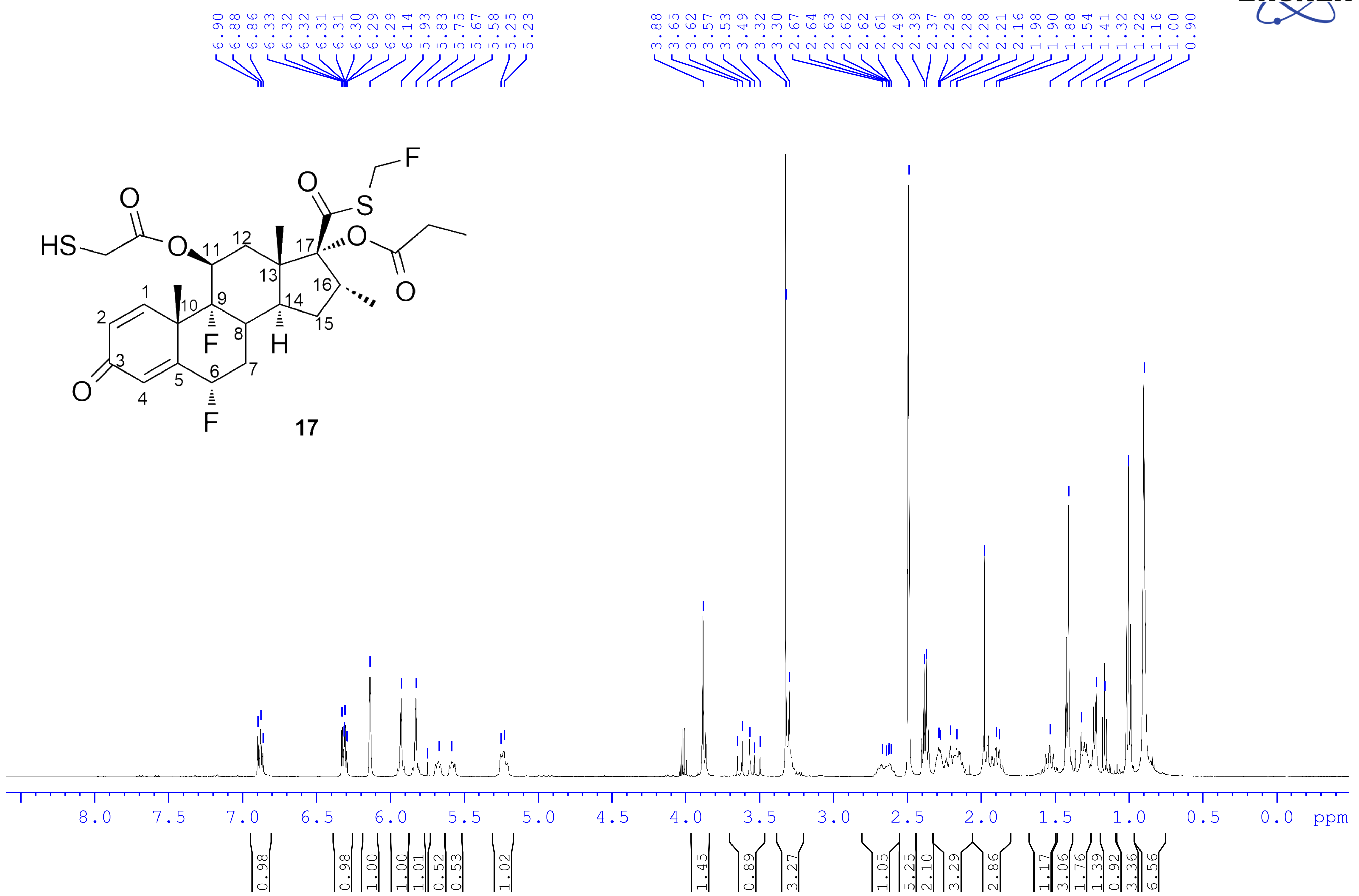
6.90 6.88 6.86 6.33 6.32 6.32 6.31 6.31 6.30 6.29 6.29 6.14 5.93 5.83 5.75 5.67 5.58 5.25 5.23
3.88 3.65 3.62 3.57 3.53 3.49 3.32 3.30 2.67 2.64 2.63 2.62 2.62 2.61 2.49 2.39 2.37 2.29 2.28 2.28 2.21 2.16 1.98 1.90 1.88 1.54 1.41 1.32 1.22 1.16 1.00 0.90
HS
O
O
O
F
S
O
O
H
F
F
O
1 2 3 4 5 6 7 8 9 10 11 12 13 14 15 16 17
17
8.0 7.5 7.0 6.5 6.0 5.5 5.0 4.5 4.0 3.5 3.0 2.5 2.0 1.5 1.0 0.5 0.0 ppm
0.98 0.98 1.00 1.00 1.01 0.52 0.53 1.02 1.45 0.89 3.27 1.05 5.25 2.10 3.29 2.86 1.17 3.06 1.76 1.39 0.92 3.36 6.56

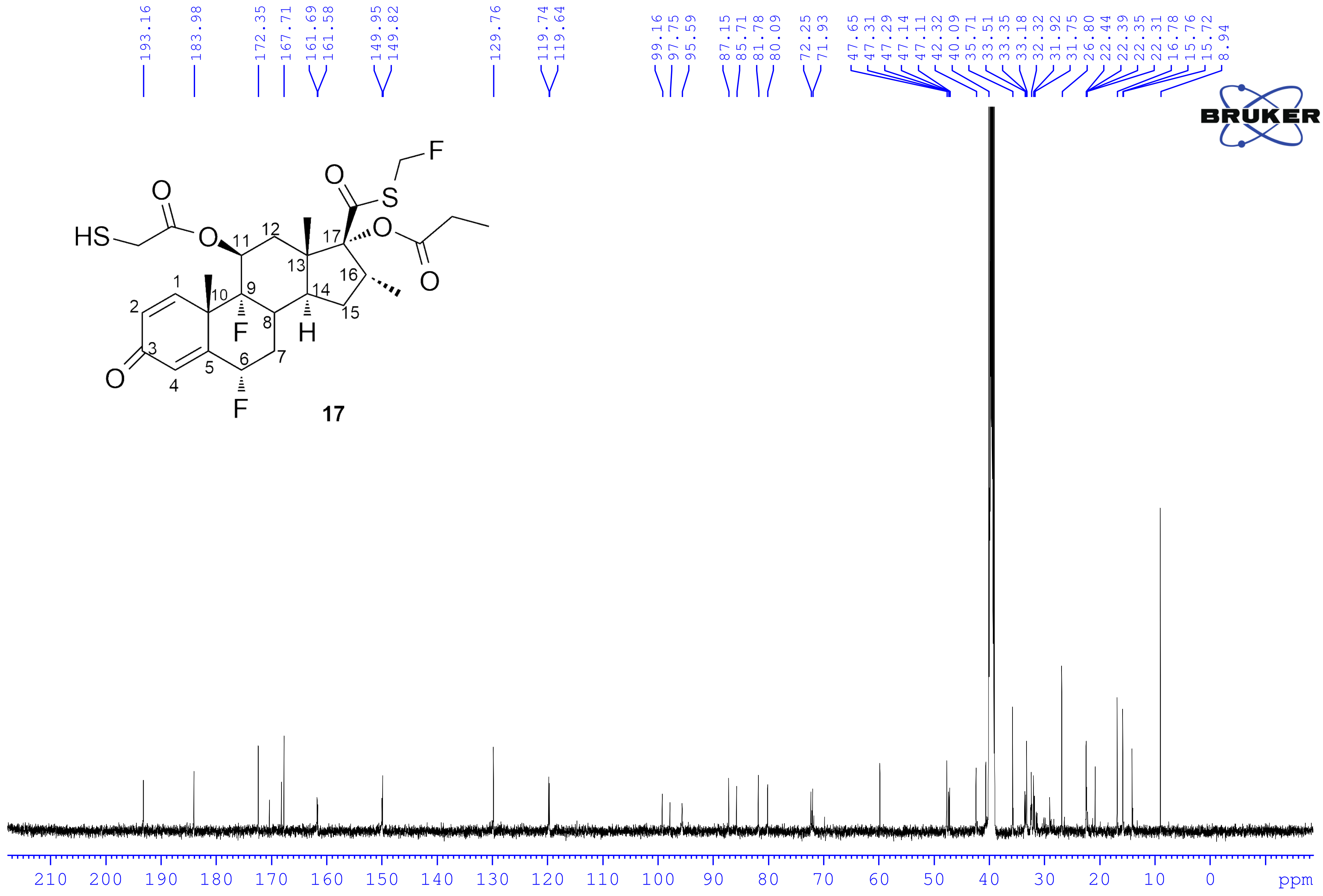
193.16
183.98
172.35
167.71
161.69
161.58
149.95
149.82
129.76
119.74
119.64
99.16
97.75
95.59
87.15
85.71
81.78
80.09
72.25
71.93
47.65
47.31
47.29
47.14
47.11
42.32
40.09
35.71
33.51
33.35
33.18
32.32
31.92
31.75
26.80
22.44
22.39
22.35
22.31
16.78
15.76
15.72
8.94
210 200 190 180 170 160 150 140 130 120 110 100 90 80 70 60 50 40 30 20 10 0 ppm
HS
O
F
S
H
O

17

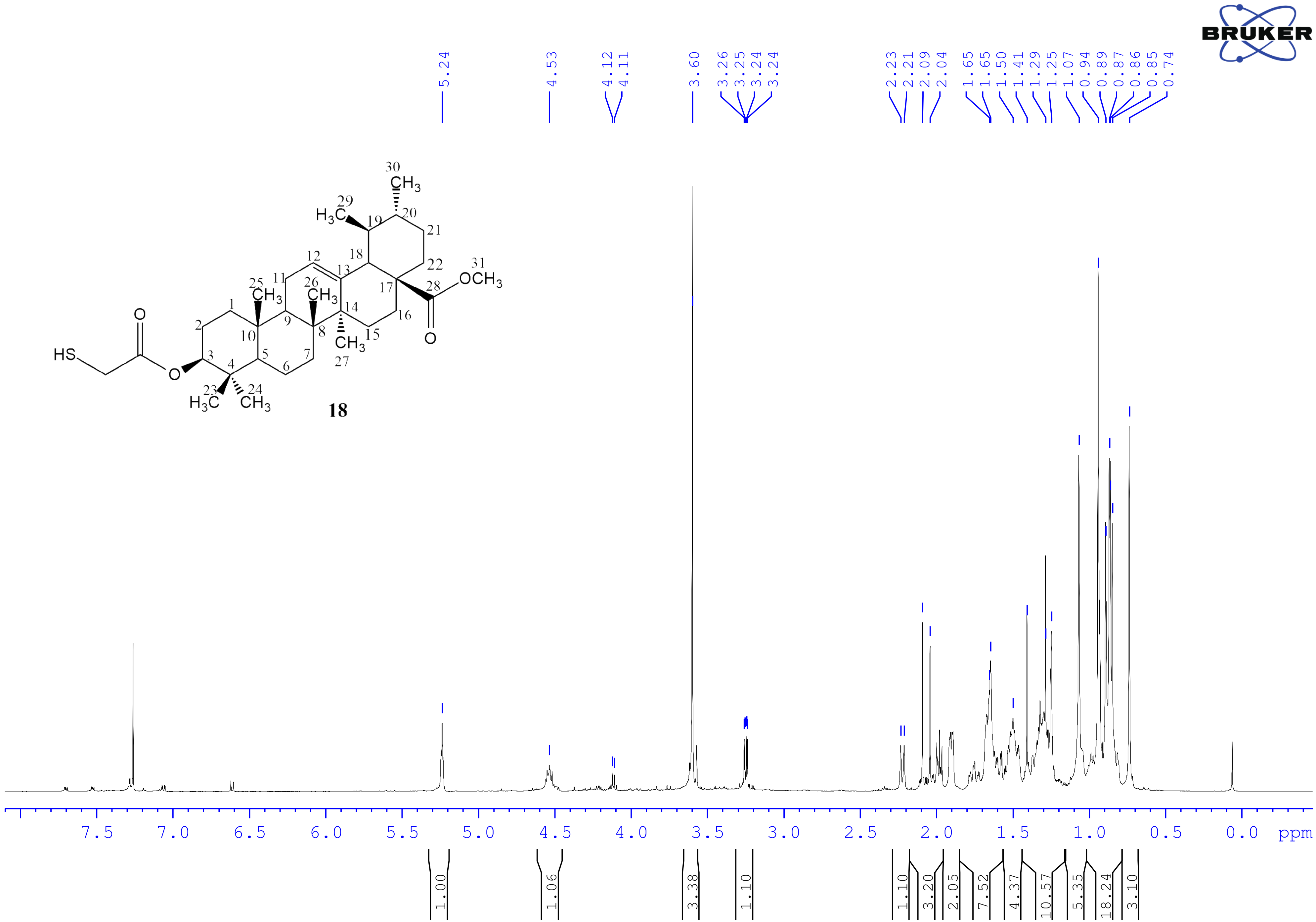

BRUKER
5.24
4.53
4.12
4.11
3.60
3.26
3.25
3.24
3.24
2.23
2.21
2.09
2.04
1.65
1.65
1.50
1.41
1.29
1.25
1.07
0.94
0.89
0.87
0.86
0.85
0.74
HS
O
CH3
OCH3
H3C
18
7.5
7.0
6.5
6.0
5.5
5.0
4.5
4.0
3.5
3.0
2.5
2.0
1.5
1.0
0.5
0.0
ppm
1.00
1.06
3.38
1.10
1.10
3.20
2.05
7.52
4.37
10.57
5.35
18.24
3.10

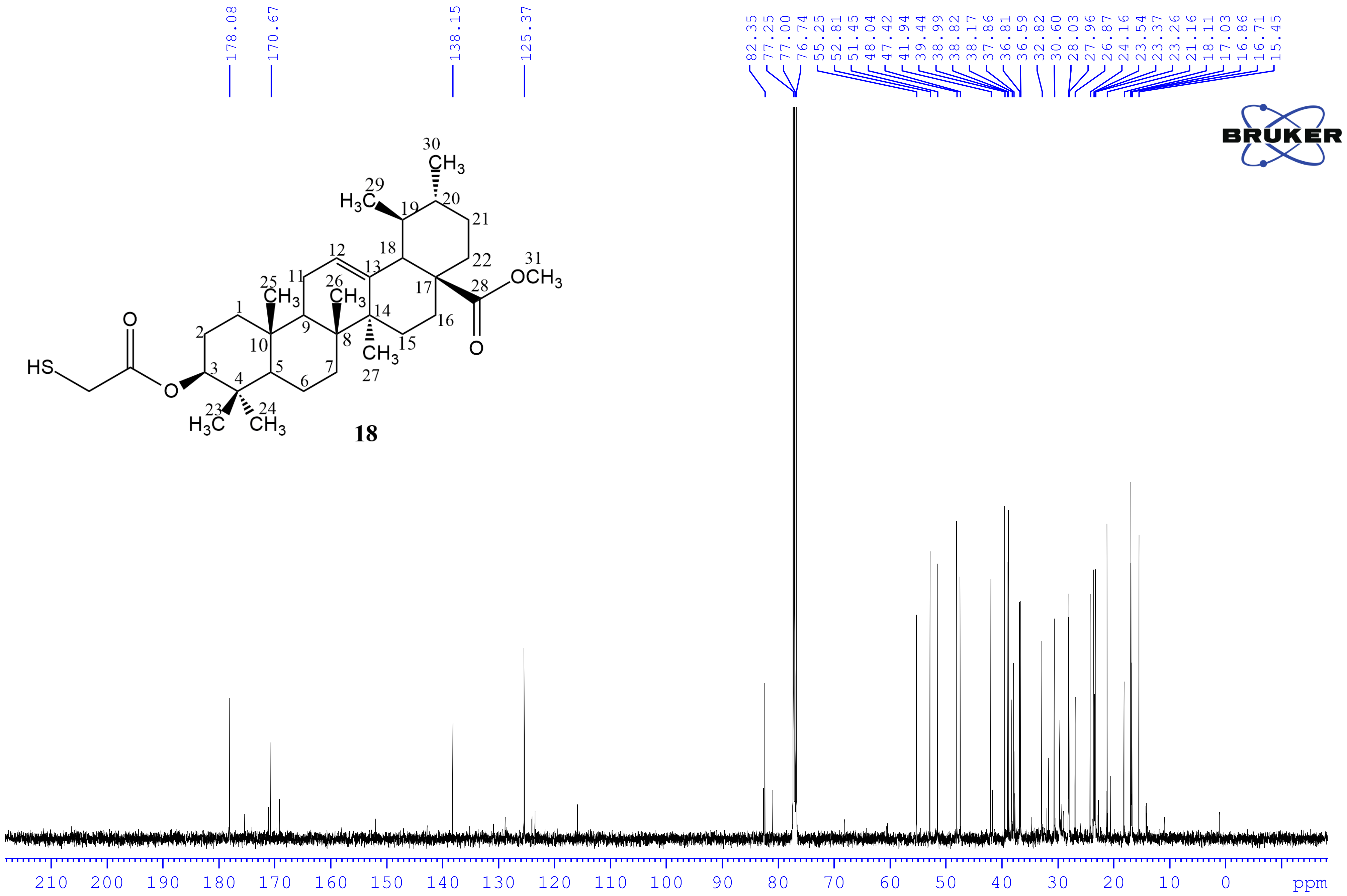
178.08
170.67
138.15
125.37
82.35
77.25
77.00
76.74
55.25
52.81
51.45
48.04
47.42
41.94
39.44
38.99
38.82
38.17
37.86
36.81
36.59
32.82
30.60
28.03
27.96
26.87
24.16
23.54
23.37
23.26
21.16
18.11
17.03
16.86
16.71
15.45
BRUKER
18
210 200 190 180 170 160 150 140 130 120 110 100 90 80 70 60 50 40 30 20 10 0 ppm

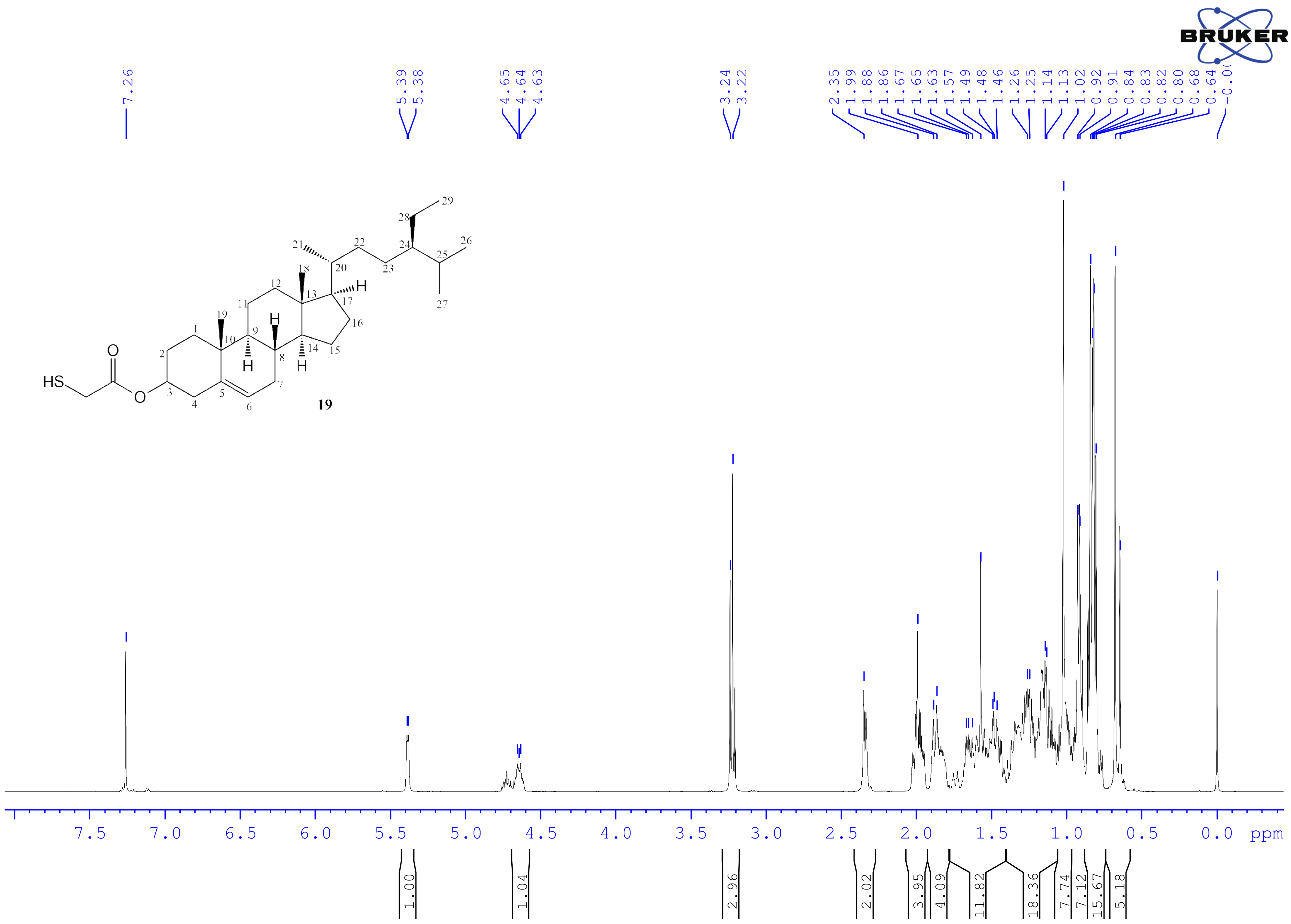
7.26
5.39
5.38
4.65
4.64
4.63
3.24
3.22
2.35
1.99
1.88
1.86
1.67
1.65
1.63
1.57
1.49
1.48
1.46
1.26
1.25
1.14
1.13
1.02
0.92
0.91
0.84
0.83
0.82
0.80
0.68
0.64
HS
O
19
7.5
7.0
6.5
6.0
5.5
5.0
4.5
4.0
3.5
3.0
2.5
2.0
1.5
1.0
0.5
0.0
ppm
1.00
1.04
2.96
2.02
3.95
4.09
11.82
18.36
7.74
7.12
15.67
5.18

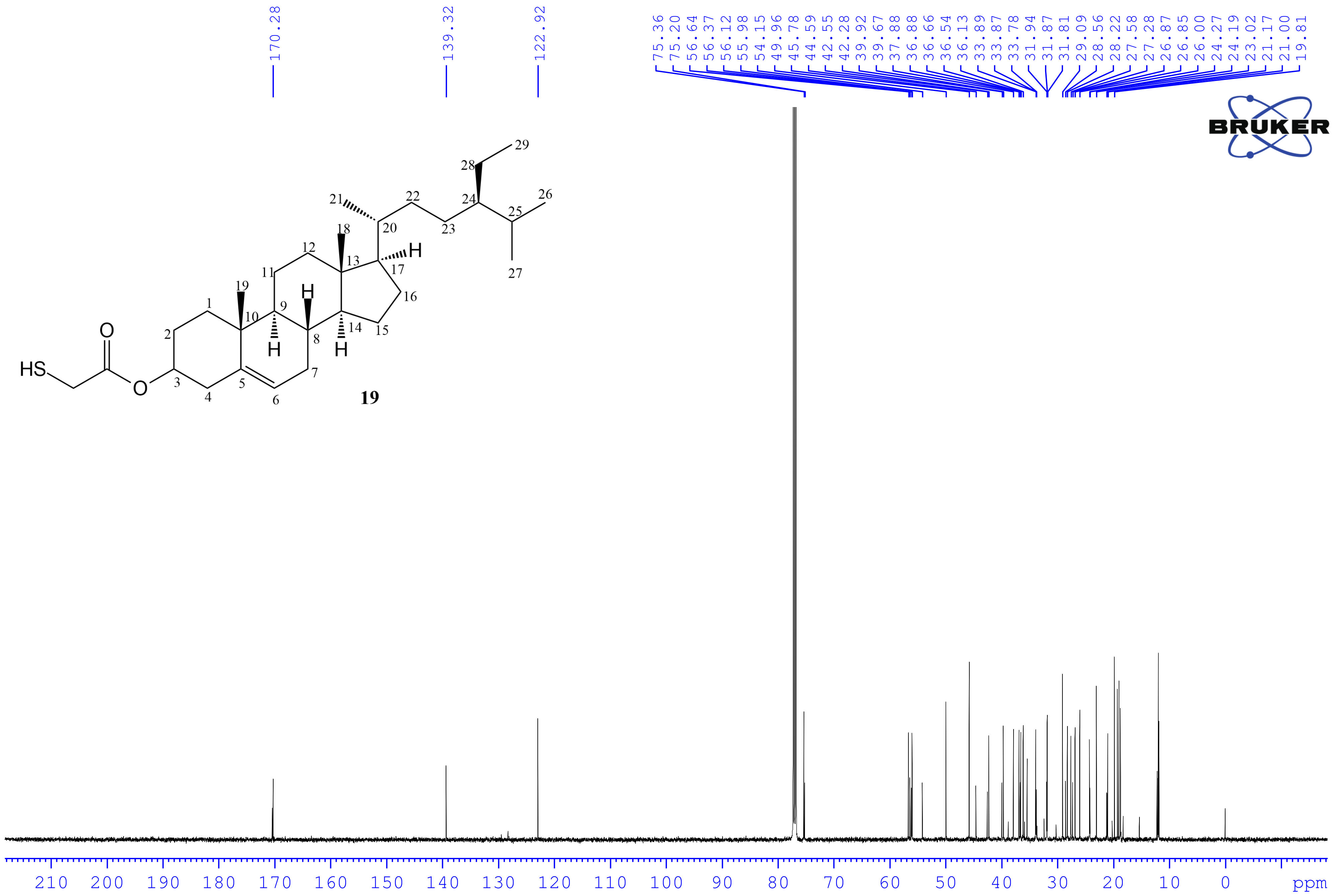
170.28
139.32
122.92
75.36
75.20
56.64
56.37
56.12
55.98
54.15
49.96
45.78
44.59
42.55
42.28
39.92
39.67
37.88
36.88
36.66
36.54
36.13
33.89
33.87
33.78
31.94
31.87
31.81
29.09
28.56
28.22
27.58
27.28
26.87
26.85
26.00
24.27
24.19
23.02
21.17
21.00
19.81
BRUKER
HS
O
19
210 200 190 180 170 160 150 140 130 120 110 100 90 80 70 60 50 40 30 20 10 0 ppm

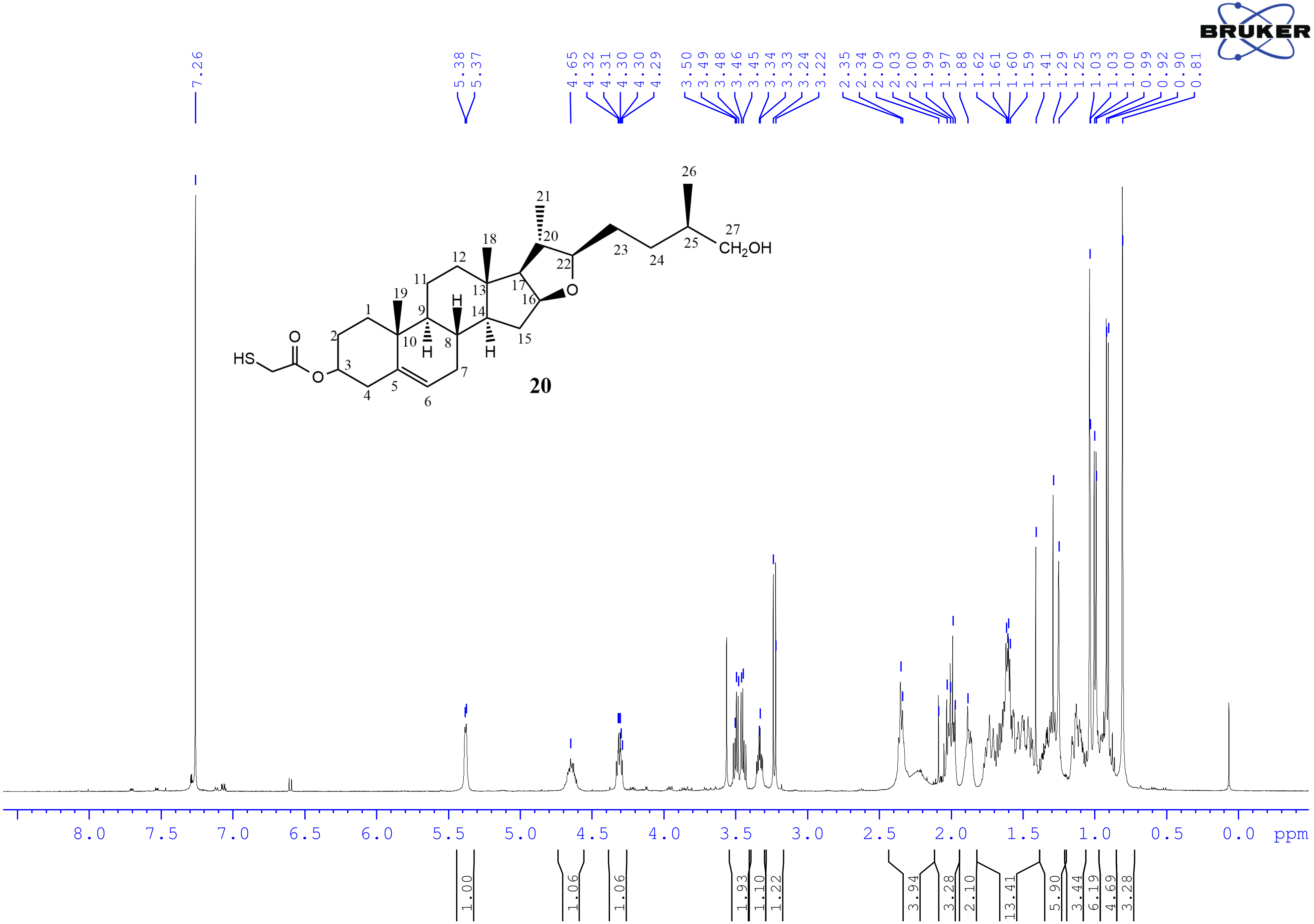

BRUKER
7.26
5.38
5.37
4.65
4.32
4.31
4.30
4.30
4.29
3.50
3.49
3.48
3.46
3.45
3.34
3.33
3.24
3.22
2.35
2.34
2.09
2.03
2.00
1.99
1.97
1.88
1.62
1.61
1.60
1.59
1.41
1.29
1.25
1.03
1.03
1.00
0.99
0.92
0.90
0.81
HS
O
20
CH2OH
8.0 7.5 7.0 6.5 6.0 5.5 5.0 4.5 4.0 3.5 3.0 2.5 2.0 1.5 1.0 0.5 0.0 ppm
1.00
1.06
1.06
1.93
1.10
1.22
3.94
3.28
2.10
13.41
5.90
3.44
6.19
4.69
3.28

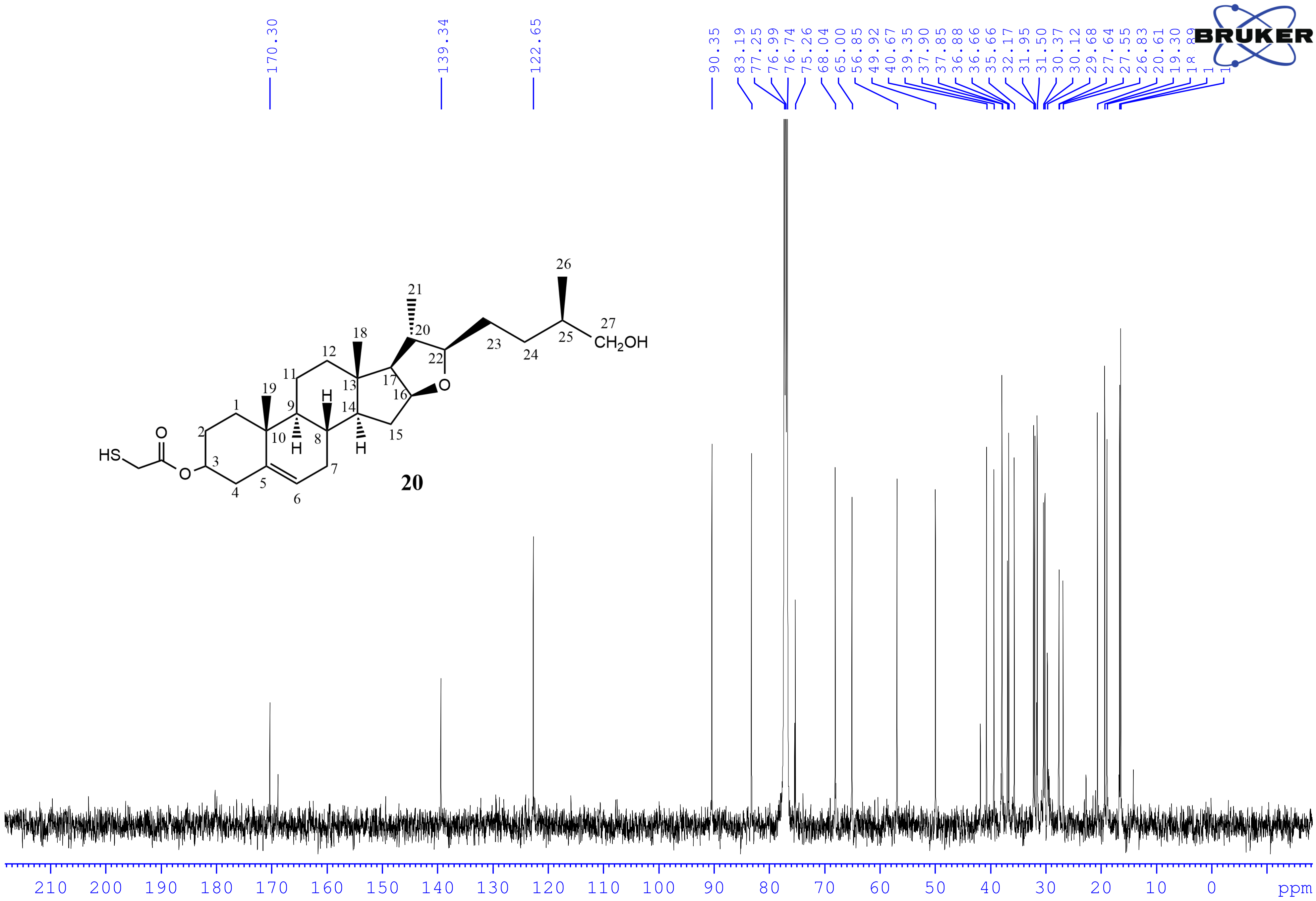

170.30
139.34
122.65
90.35
83.19
77.25
76.99
76.74
75.26
68.04
65.00
56.85
49.92
40.67
39.35
37.90
37.85
36.88
36.66
35.66
32.17
31.95
31.50
30.37
30.12
29.68
27.64
27.55
26.83
20.61
19.30
BRUKER
HS
O
CH2OH
20
210 200 190 180 170 160 150 140 130 120 110 100 90 80 70 60 50 40 30 20 10 0 ppm

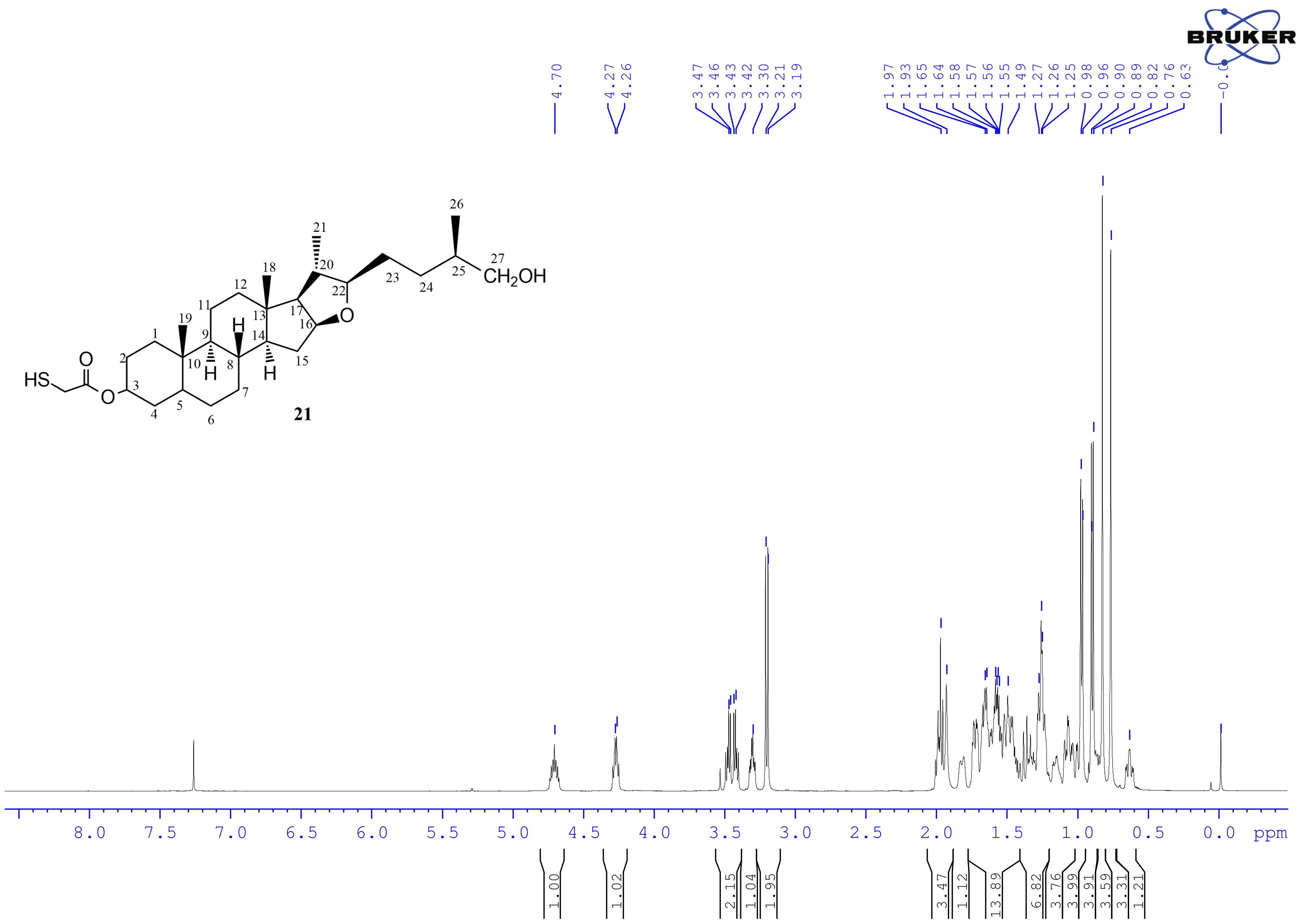
BRUKER
4.70
4.27
4.26
3.47
3.46
3.43
3.42
3.30
3.21
3.19
1.97
1.93
1.65
1.64
1.58
1.57
1.56
1.55
1.49
1.27
1.26
1.25
0.98
0.96
0.90
0.89
0.82
0.76
0.63
-0.0
HS
O
O
CH2OH
21
8.0 7.5 7.0 6.5 6.0 5.5 5.0 4.5 4.0 3.5 3.0 2.5 2.0 1.5 1.0 0.5 0.0 ppm
1.00
1.02
2.15
1.04
1.95
3.47
1.12
13.89
6.82
3.76
3.99
3.91
3.59
3.31
1.21

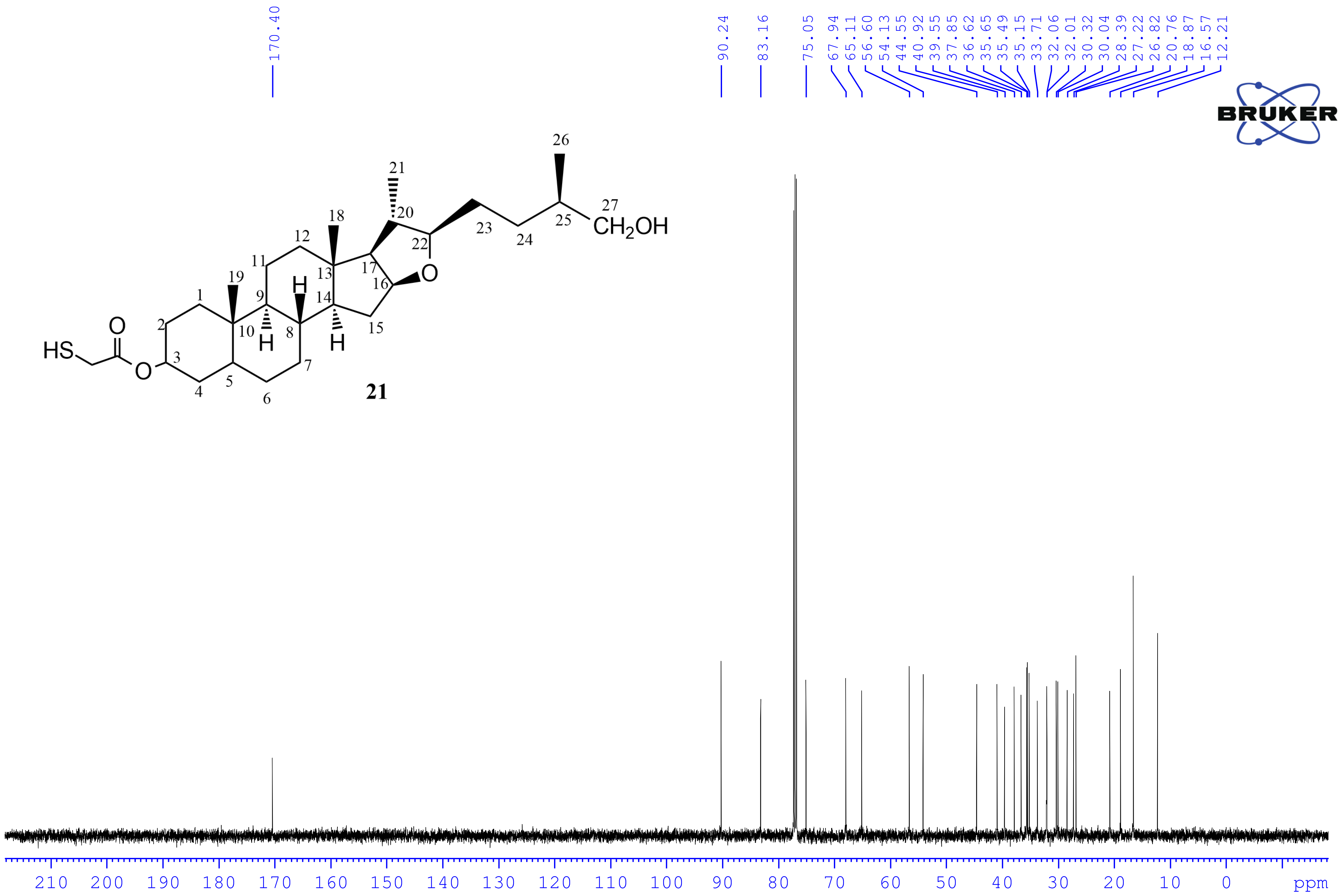
170.40
90.24
83.16
75.05
67.94
65.11
56.60
54.13
44.55
40.92
39.55
37.85
36.62
35.65
35.49
35.15
33.71
32.06
32.01
30.32
30.04
28.39
27.22
26.82
20.76
18.87
16.57
12.21
BRUKER
HS
O
CH2OH
210 200 190 180 170 160 150 140 130 120 110 100 90 80 70 60 50 40 30 20 10 0 ppm

21

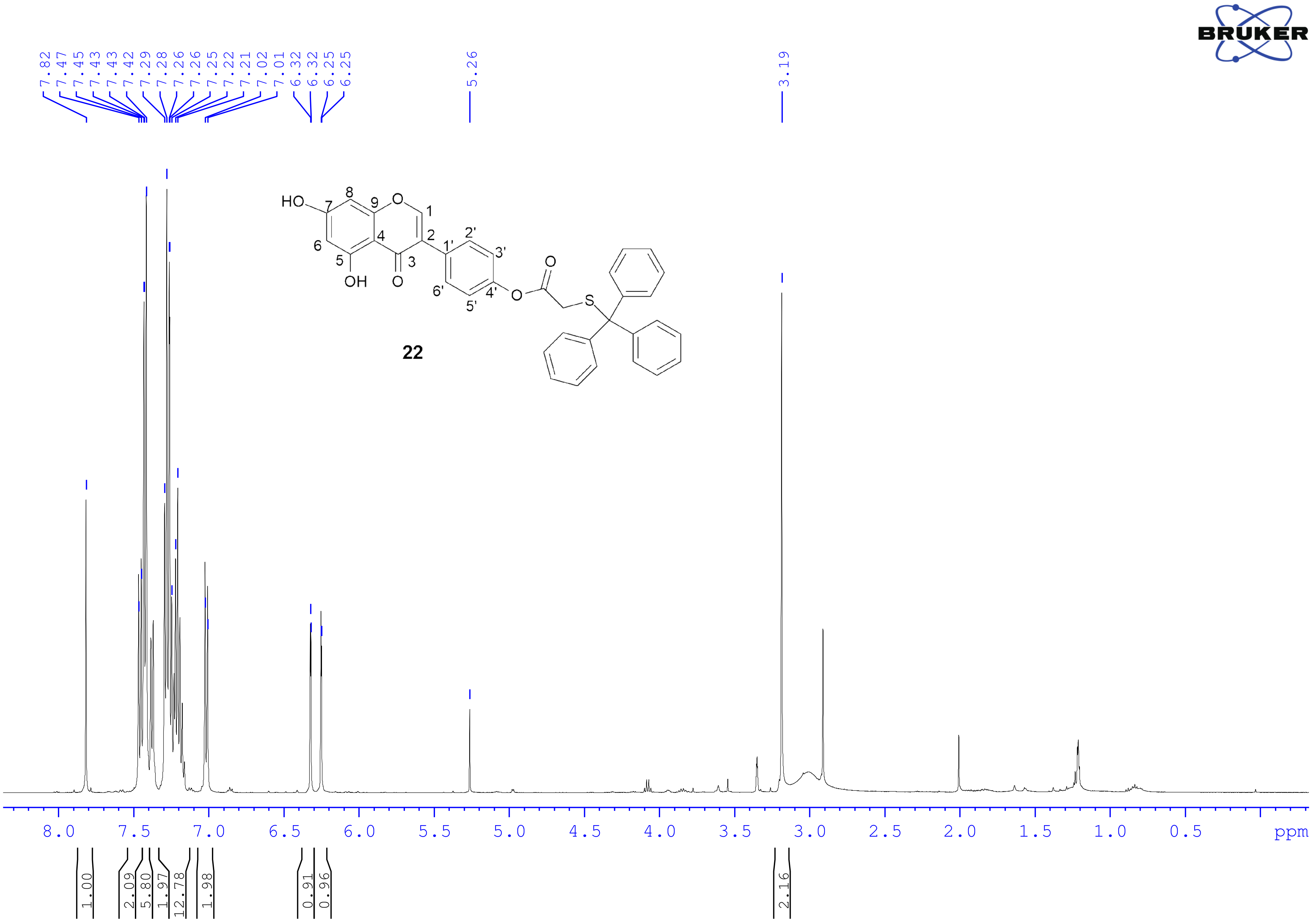
BRUKER
7.82
7.47
7.45
7.43
7.43
7.42
7.29
7.28
7.26
7.26
7.25
7.22
7.21
7.02
7.01
6.32
6.32
6.25
6.25
5.26
3.19
HO
OH
O
S
22
8.0 7.5 7.0 6.5 6.0 5.5 5.0 4.5 4.0 3.5 3.0 2.5 2.0 1.5 1.0 0.5 ppm
1.00
2.09
5.80
1.97
12.78
1.98
0.91
0.96
2.16

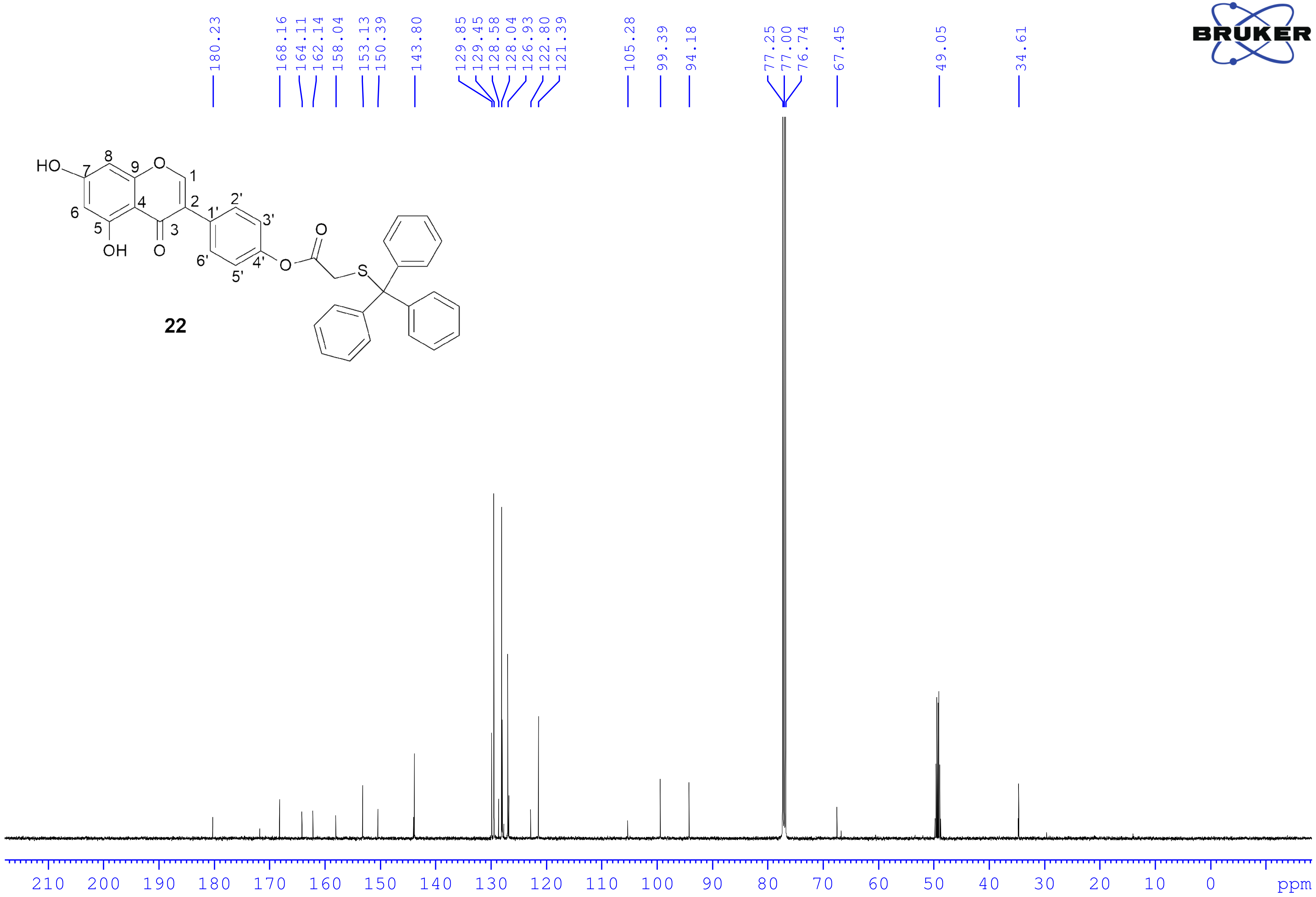
180.23
168.16
164.11
162.14
158.04
153.13
150.39
143.80
129.85
129.45
128.58
128.04
126.93
122.80
121.39
105.28
99.39
94.18
77.25
77.00
76.74
67.45
49.05
34.61
BRUKER
HO
OH
O
S
22
210 200 190 180 170 160 150 140 130 120 110 100 90 80 70 60 50 40 30 20 10 0 ppm

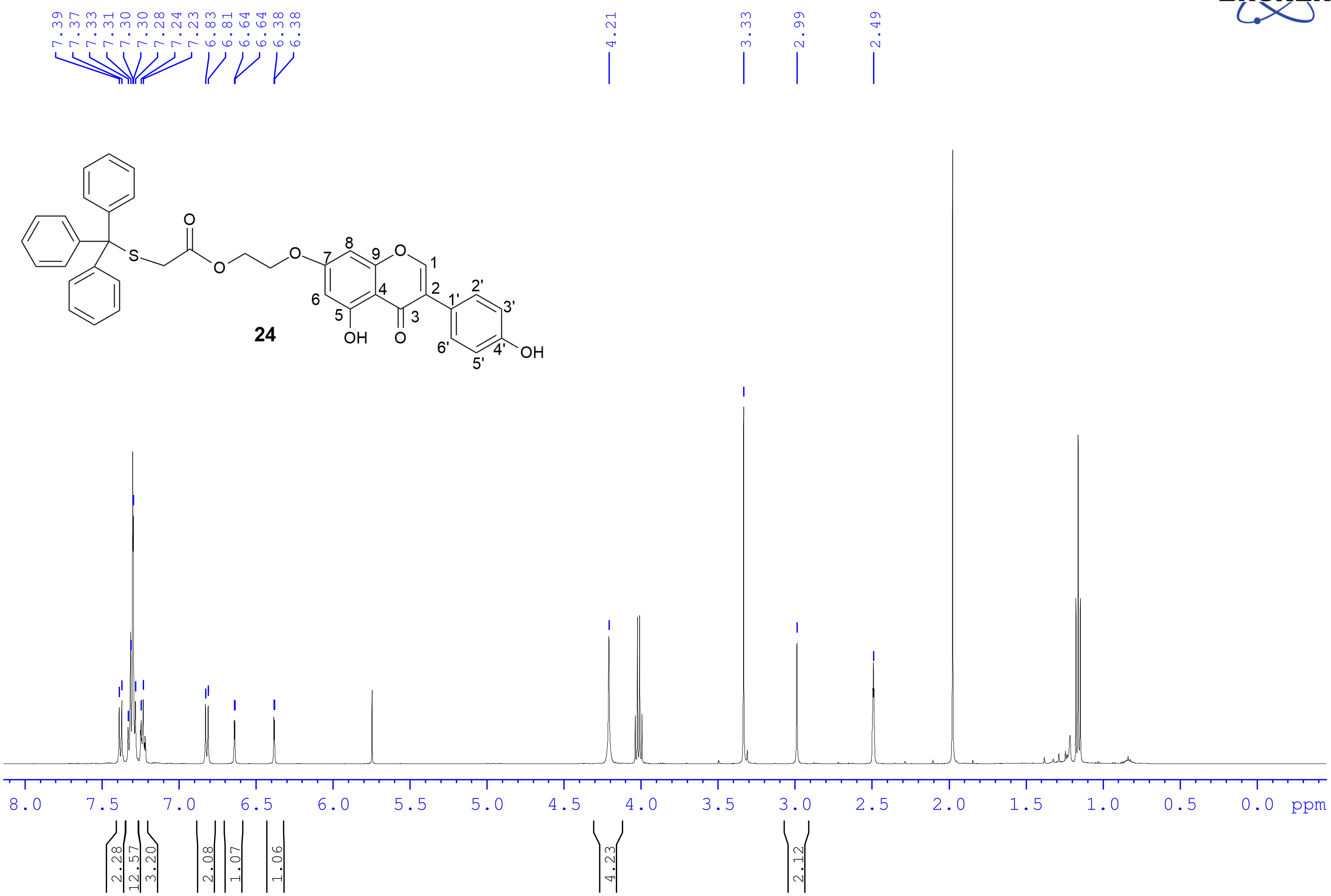
7.39 7.37 7.33 7.31 7.30 7.30 7.28 7.24 7.23 6.83 6.81 6.64 6.64 6.38 6.38
4.21
3.33
2.99
2.49
O
S
O
O
7
8
9
O
1
2
2'
3'
1'
6
5
4
3
24
OH
O
6'
4'
OH
5'
8.0 7.5 7.0 6.5 6.0 5.5 5.0 4.5 4.0 3.5 3.0 2.5 2.0 1.5 1.0 0.5 0.0 ppm
2.28
12.57
3.20
2.08
1.07
1.06
4.23
2.12

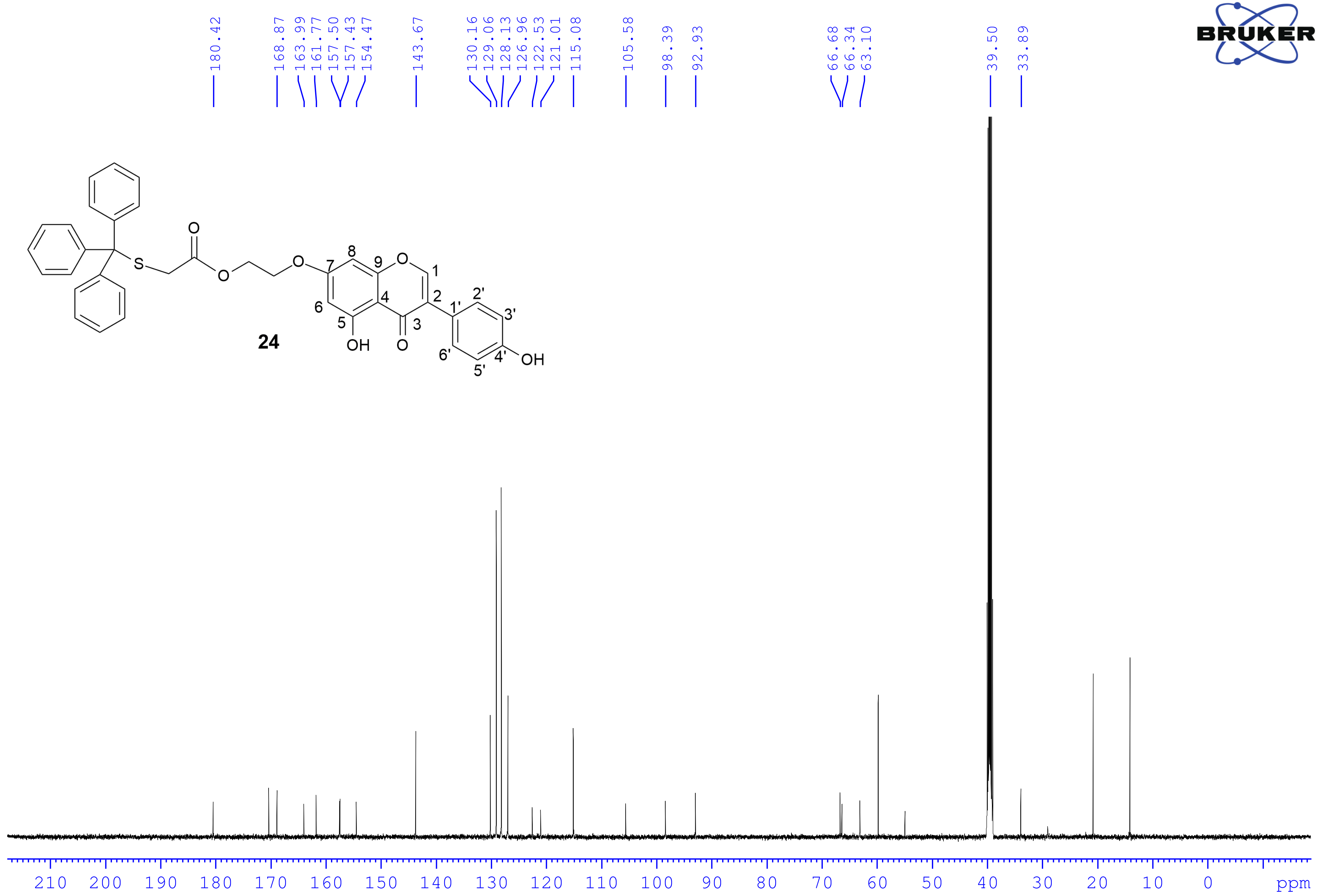
180.42
168.87
163.99
161.77
157.50
157.43
154.47
143.67
130.16
129.06
128.13
126.96
122.53
121.01
115.08
105.58
98.39
92.93
66.68
66.34
63.10
39.50
33.89
24
210 200 190 180 170 160 150 140 130 120 110 100 90 80 70 60 50 40 30 20 10 0 ppm

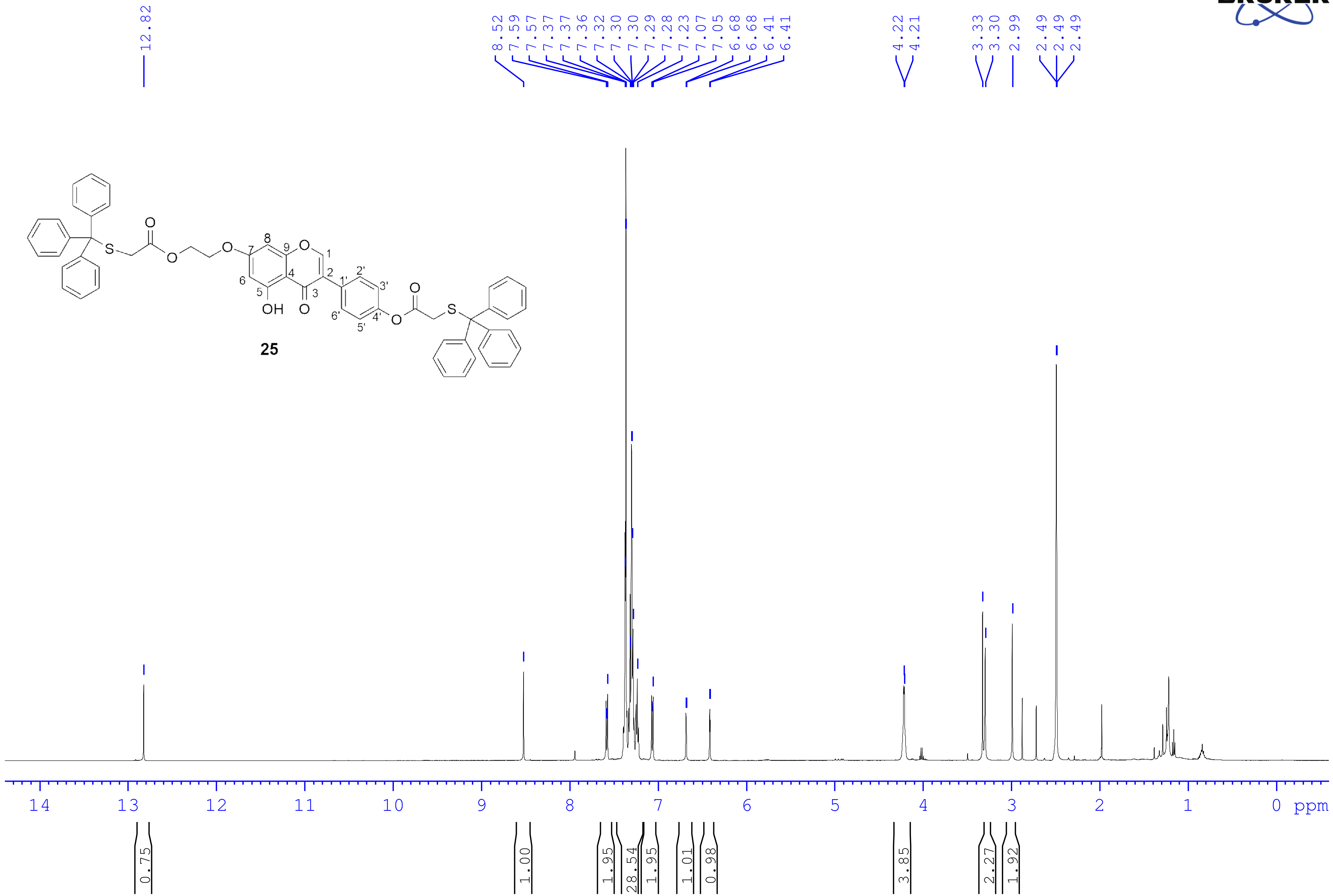
12.82
8.52 7.59 7.57 7.37 7.37 7.36 7.32 7.30 7.30 7.29 7.28 7.23 7.07 7.05 6.68 6.68 6.41 6.41
4.22 4.21
3.33 3.30 2.99 2.49 2.49 2.49
25
14 13 12 11 10 9 8 7 6 5 4 3 2 1 0 ppm
0.75 1.00 1.95 28.54 1.95 1.01 0.98 3.85 2.27 1.92

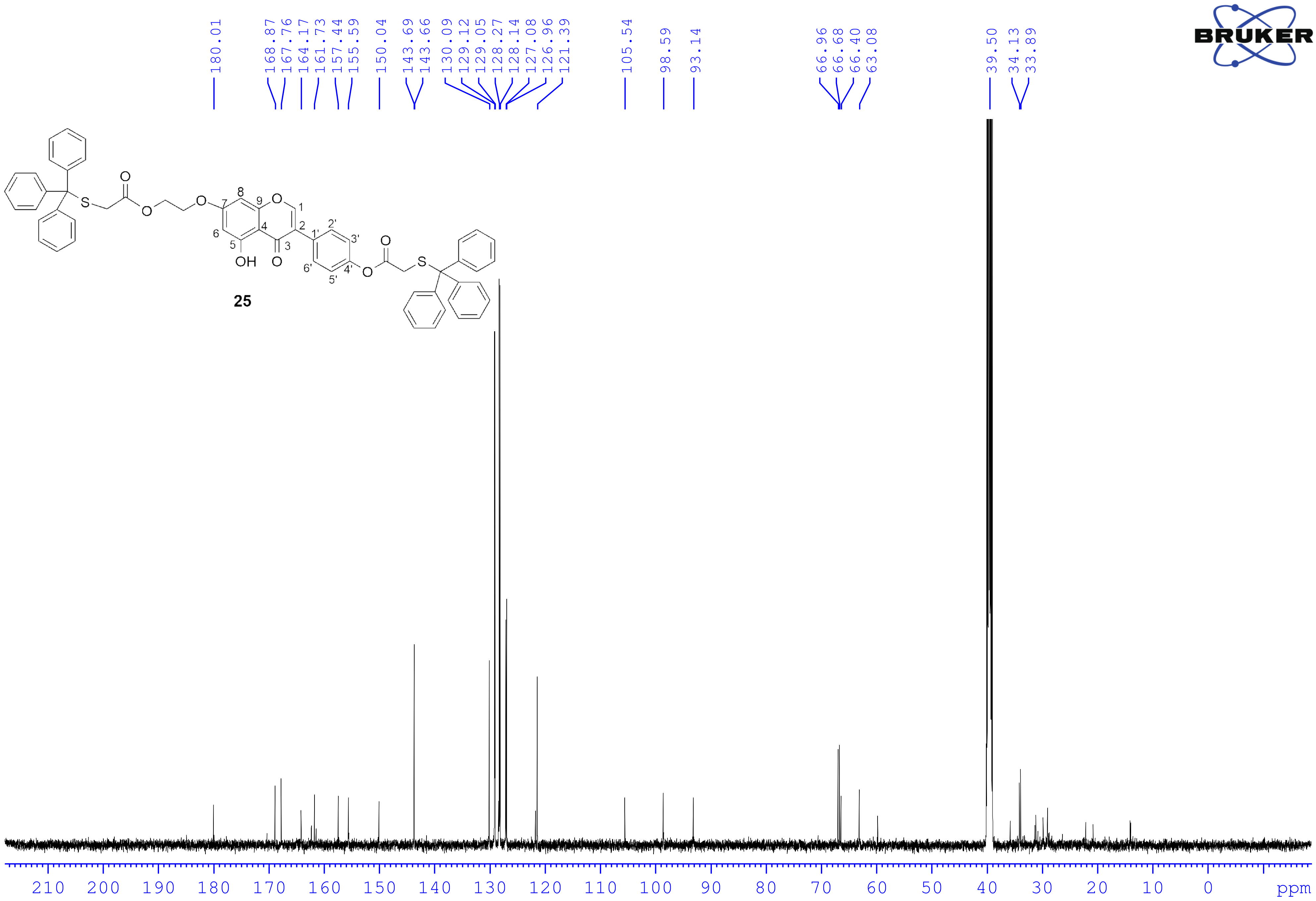
180.01
168.87
167.76
164.17
161.73
157.44
155.59
150.04
143.69
143.66
130.09
129.12
129.05
128.27
128.14
127.08
126.96
121.39
105.54
98.59
93.14
66.96
66.68
66.40
63.08
39.50
34.13
33.89
BRUKER
25
210 200 190 180 170 160 150 140 130 120 110 100 90 80 70 60 50 40 30 20 10 0 ppm

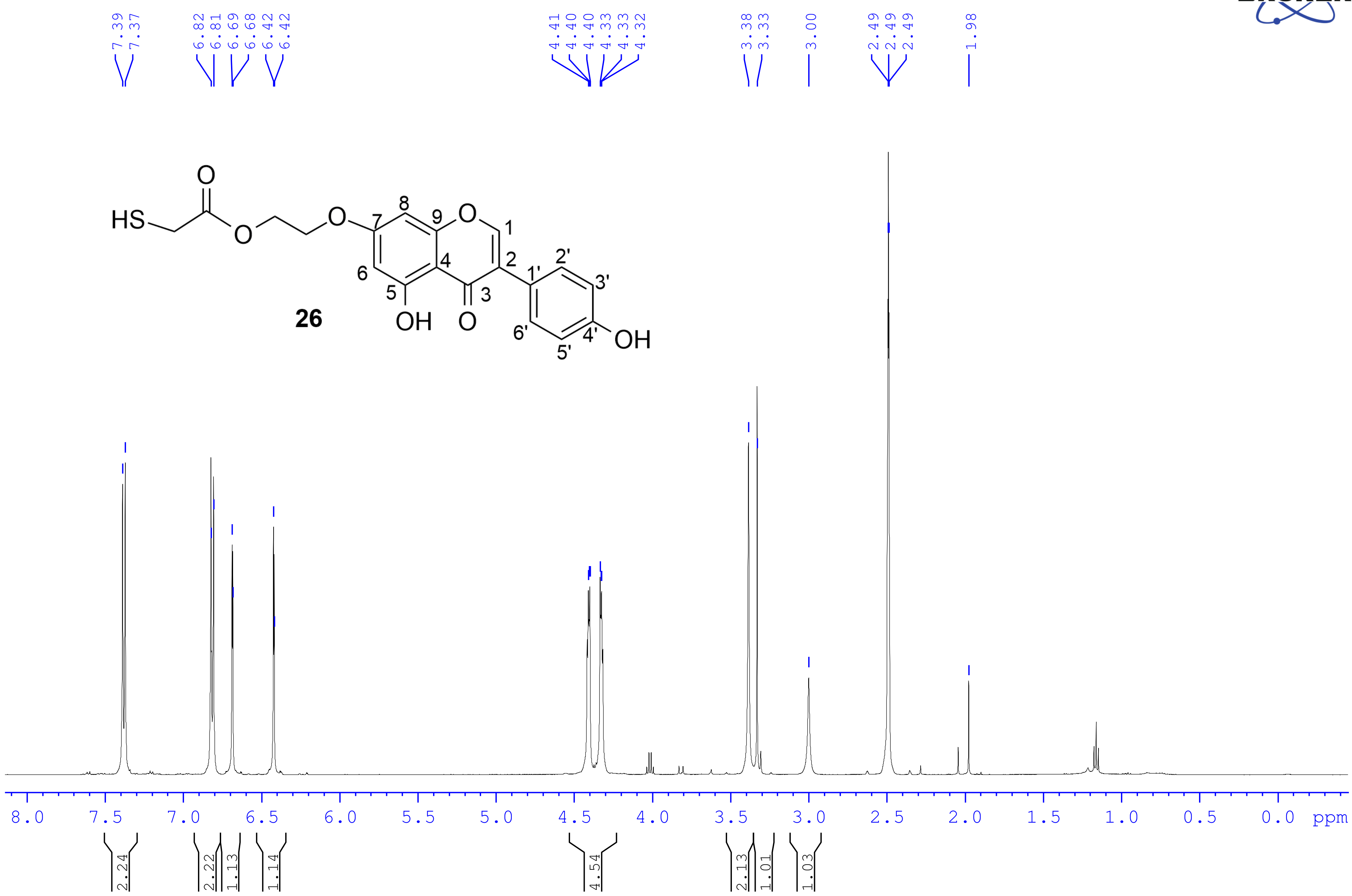
7.39
7.37
6.82
6.81
6.69
6.68
6.42
6.42
4.41
4.40
4.40
4.33
4.33
4.32
3.38
3.33
3.00
2.49
2.49
2.49
1.98
HS
O
OH
26
8.0 7.5 7.0 6.5 6.0 5.5 5.0 4.5 4.0 3.5 3.0 2.5 2.0 1.5 1.0 0.5 0.0 ppm
2.24
2.22
1.13
1.14
4.54
2.13
1.01
1.03

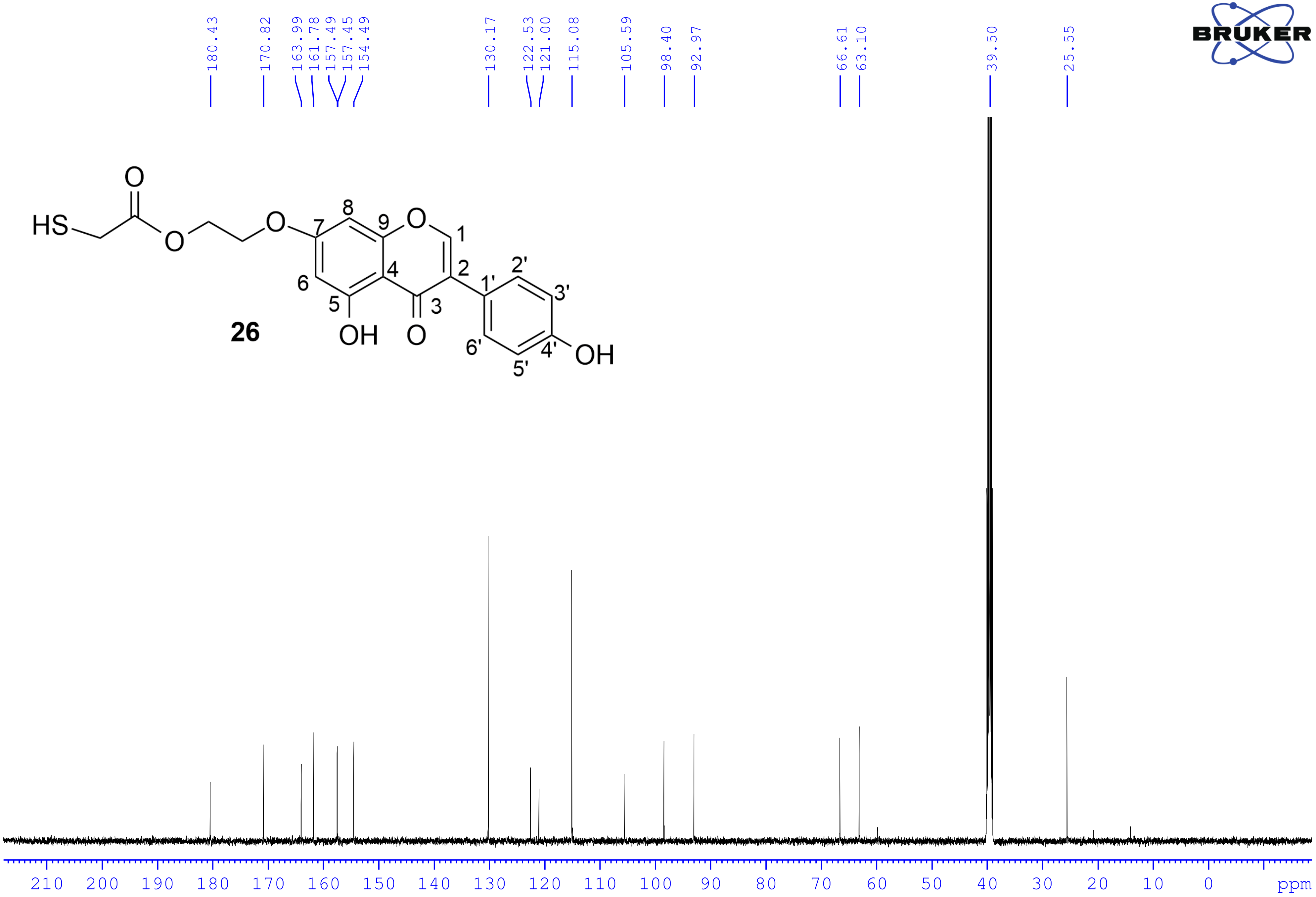
180.43
170.82
163.99
161.78
157.49
157.45
154.49
130.17
122.53
121.00
115.08
105.59
98.40
92.97
66.61
63.10
39.50
25.55
HS
O
O
O
O
OH
O
OH
26
1
2
3
4
5
6
7
8
9
1'
2'
3'
4'
5'
6'
210
200
190
180
170
160
150
140
130
120
110
100
90
80
70
60
50
40
30
20
10
0
ppm

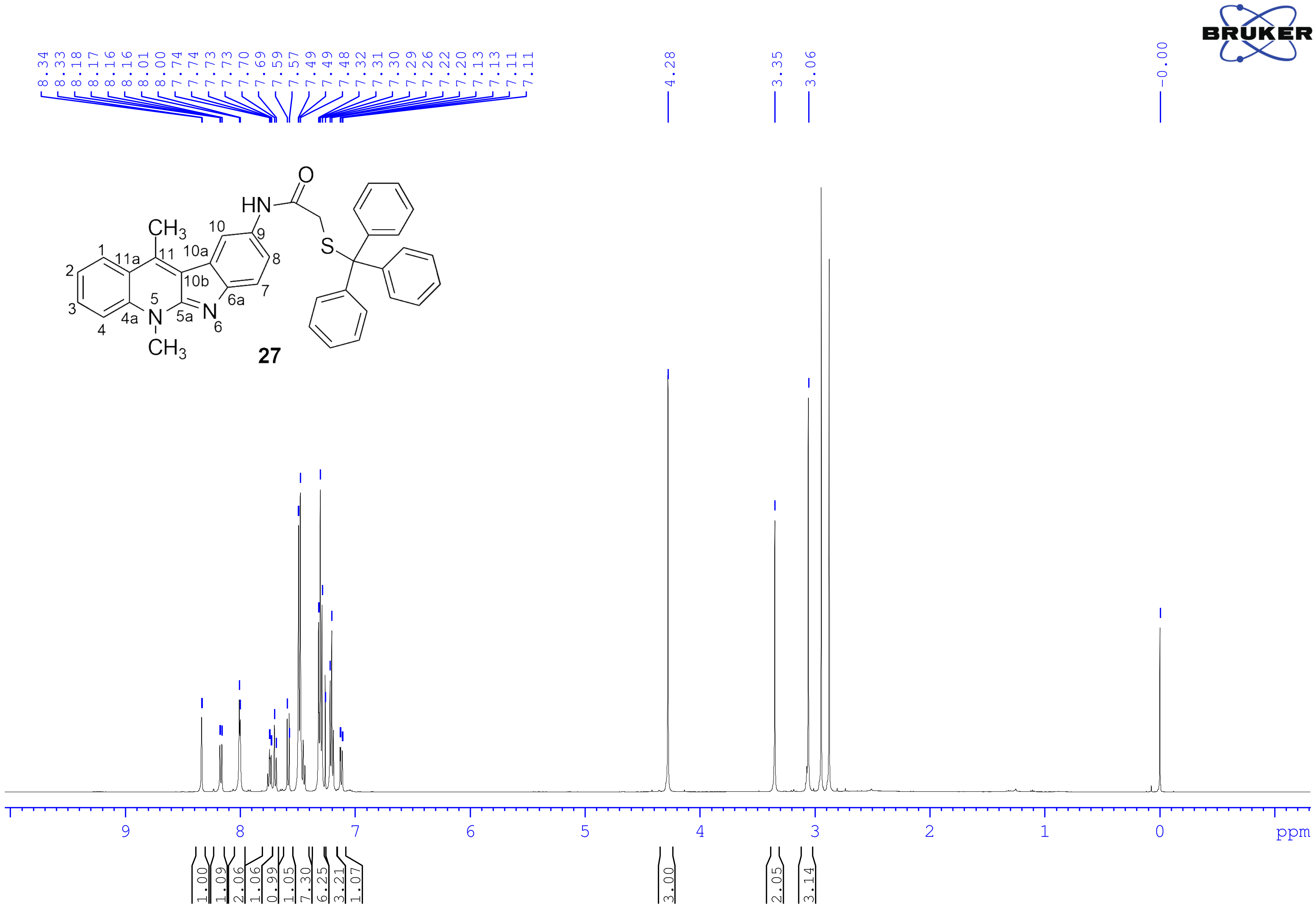
8.34 8.33 8.18 8.17 8.16 8.16 8.01 8.00 7.74 7.74 7.73 7.73 7.70 7.69 7.59 7.57 7.49 7.49 7.48 7.32 7.31 7.30 7.29 7.26 7.22 7.20 7.13 7.13 7.11 7.11
4.28
3.35
3.06
-0.00
BRUKER
O
HN
CH3
10
9
8
7
S
1
2
3
4
11a
11
10a
10b
6a
5
4a
N
5a
N
6
CH3
27
9 8 7 6 5 4 3 2 1 0 ppm
1.00 1.09 2.06 1.06 0.99 1.05 7.30 6.25 3.21 1.07
3.00
2.05
3.14

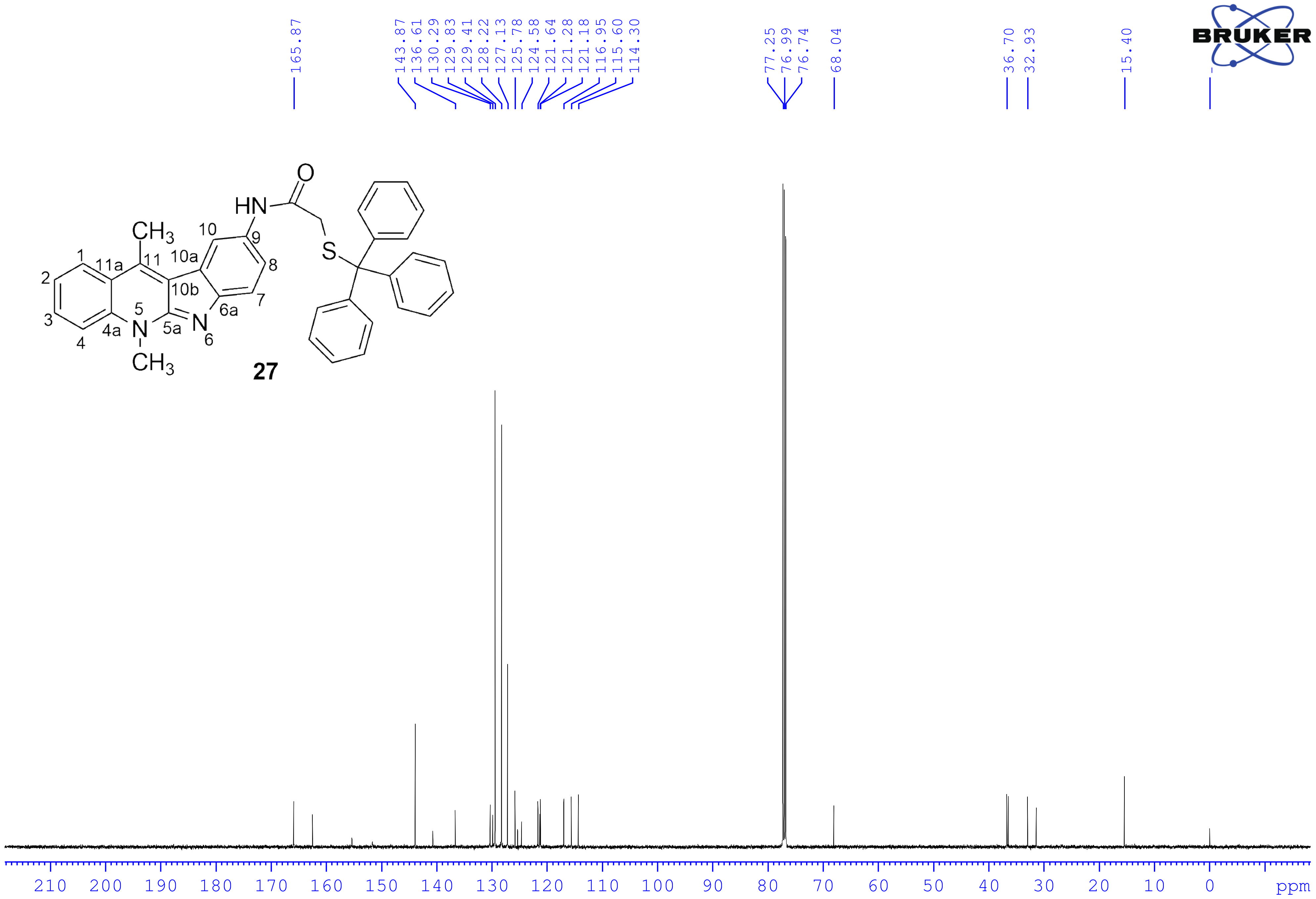

165.87
143.87
136.61
130.29
129.83
129.41
128.22
127.13
125.78
124.58
121.64
121.28
121.18
116.95
115.60
114.30
77.25
76.99
76.74
68.04
36.70
32.93
15.40
O
HN
CH3
10
9
8
7
6a
6
N
S
1
2
3
4
4a
5
5a
11a
11
10a
10b
CH3
27
210 200 190 180 170 160 150 140 130 120 110 100 90 80 70 60 50 40 30 20 10 0 ppm

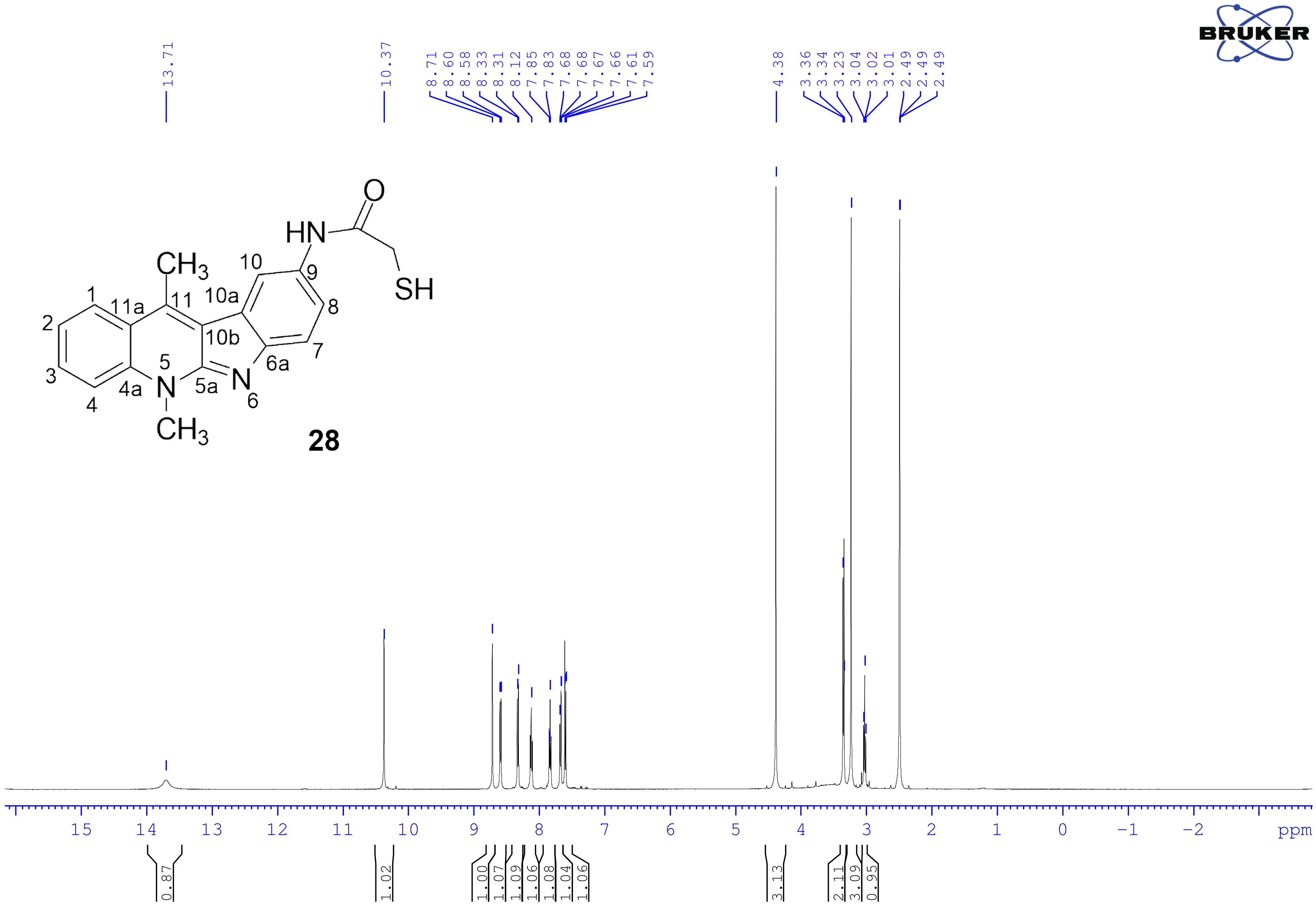
BRUKER
13.71
10.37
8.71 8.60 8.58 8.33 8.31 8.12 7.85 7.83 7.68 7.68 7.67 7.66 7.61 7.59
4.38
3.36 3.34 3.23 3.04 3.02 3.01 2.49 2.49 2.49
O
HN
SH
CH3
CH3
N
N
1 2 3 4 4a 5 5a 6 6a 7 8 9 10 10a 10b 11 11a
28
15 14 13 12 11 10 9 8 7 6 5 4 3 2 1 0 -1 -2 ppm
0.87
1.02
1.00 1.07 1.09 1.06 1.08 1.04 1.06
3.13
2.11 3.09 0.95

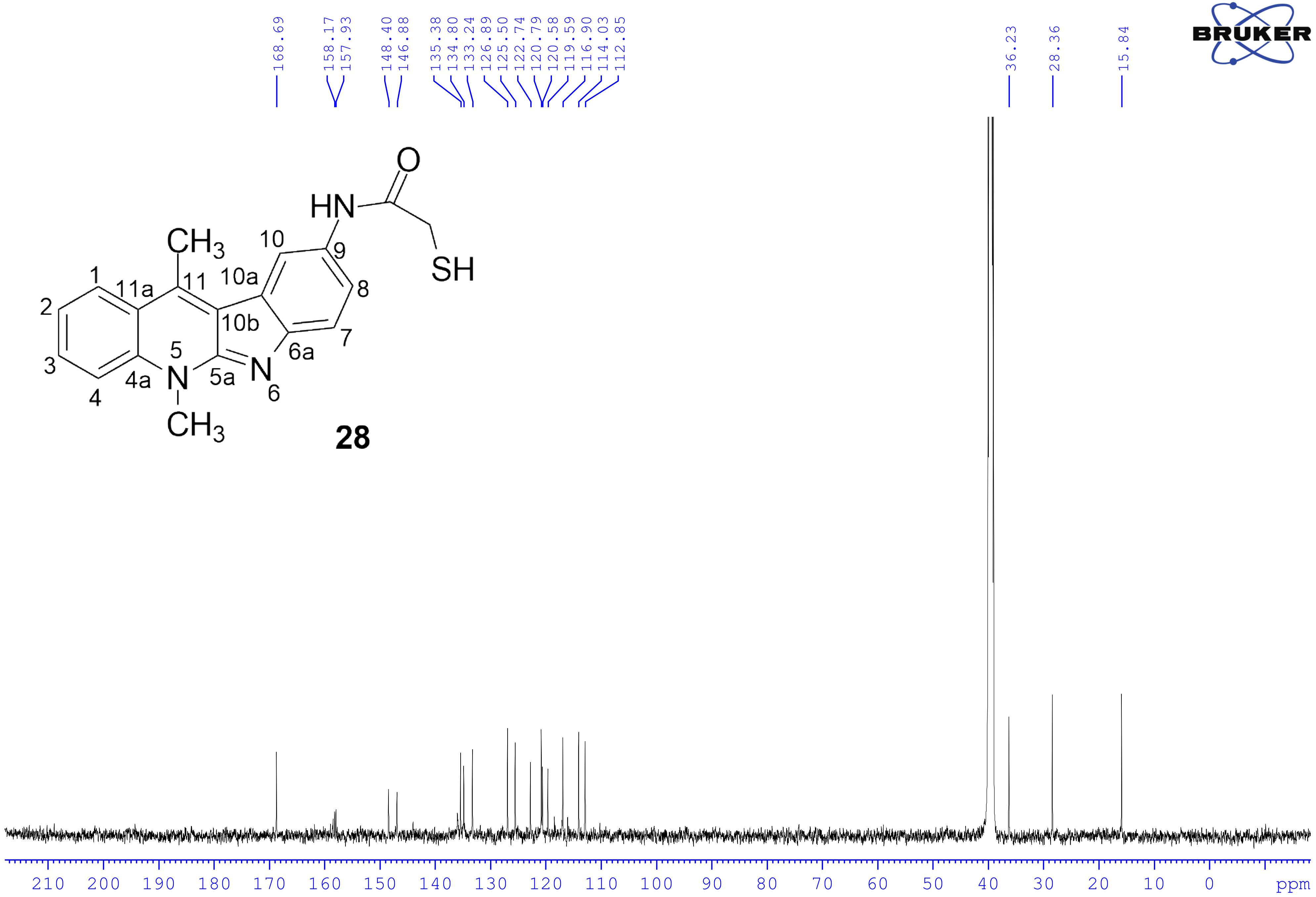
168.69
158.17
157.93
148.40
146.88
135.38
134.80
133.24
126.89
125.50
122.74
120.79
120.58
119.59
116.90
114.03
112.85
36.23
28.36
15.84
O
HN
SH
CH3
10
9
8
7
6a
N
6
5a
5
N
CH3
4a
4
3
2
1
11a
11
10a
10b
28
210
200
190
180
170
160
150
140
130
120
110
100
90
80
70
60
50
40
30
20
10
0
ppm

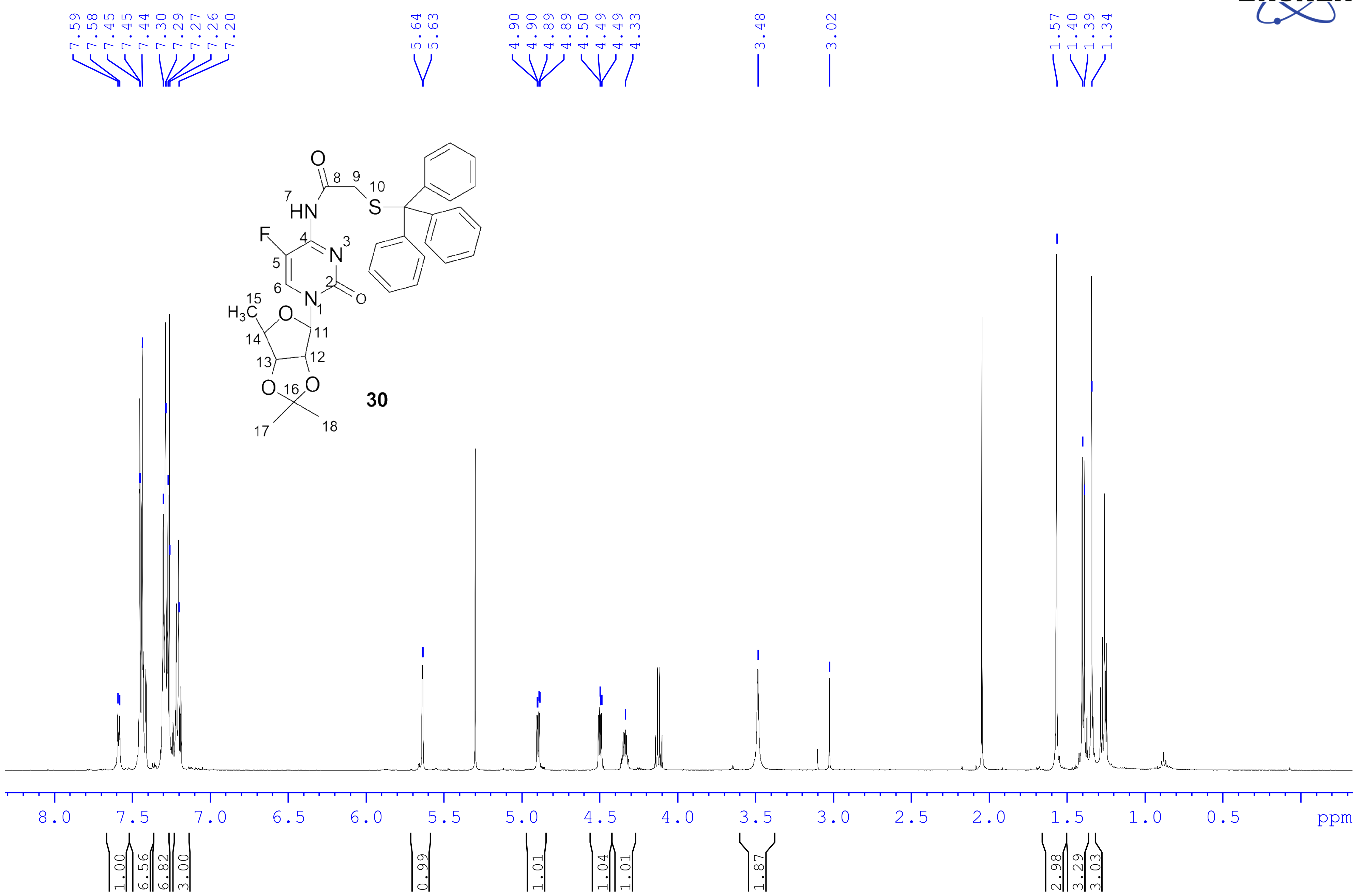
7.59 7.58 7.45 7.45 7.44 7.30 7.29 7.27 7.26 7.20
5.64 5.63
4.90 4.90 4.89 4.89 4.50 4.49 4.49 4.33
3.48
3.02
1.57 1.40 1.39 1.34
30
8.0 7.5 7.0 6.5 6.0 5.5 5.0 4.5 4.0 3.5 3.0 2.5 2.0 1.5 1.0 0.5 ppm
1.00 6.56 6.82 3.00 0.99 1.01 1.04 1.01 1.87 2.98 3.29 3.03

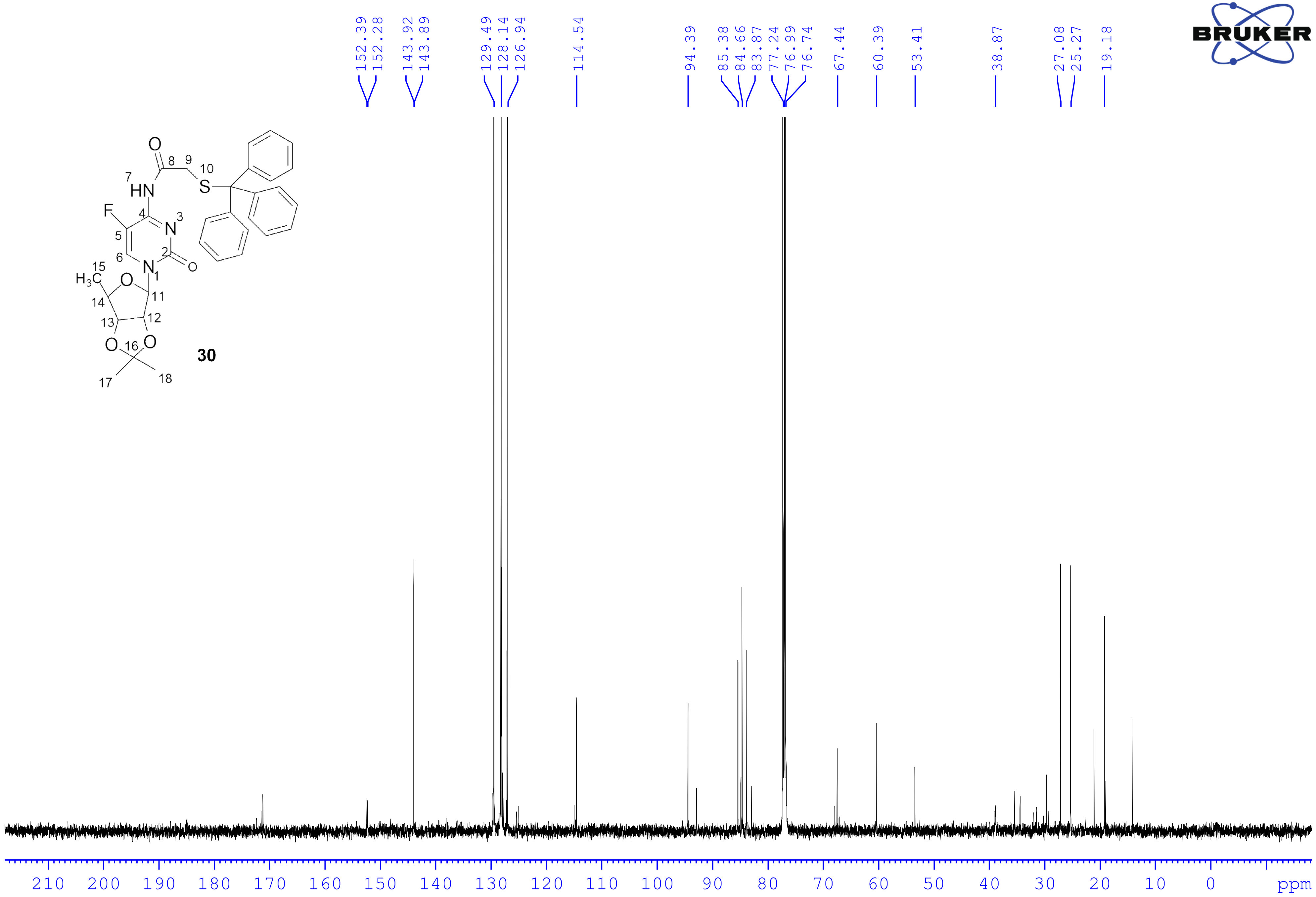

152.39
152.28
143.92
143.89
129.49
128.14
126.94
114.54
94.39
85.38
84.66
83.87
77.24
76.99
76.74
67.44
60.39
53.41
38.87
27.08
25.27
19.18
O
8
9
10
7
HN
S
F
4
3
N
5
6
2
O
N
1
15
H_3C
O
11
14
13
12
O
16
O
17
18
210 200 190 180 170 160 150 140 130 120 110 100 90 80 70 60 50 40 30 20 10 0 ppm

30

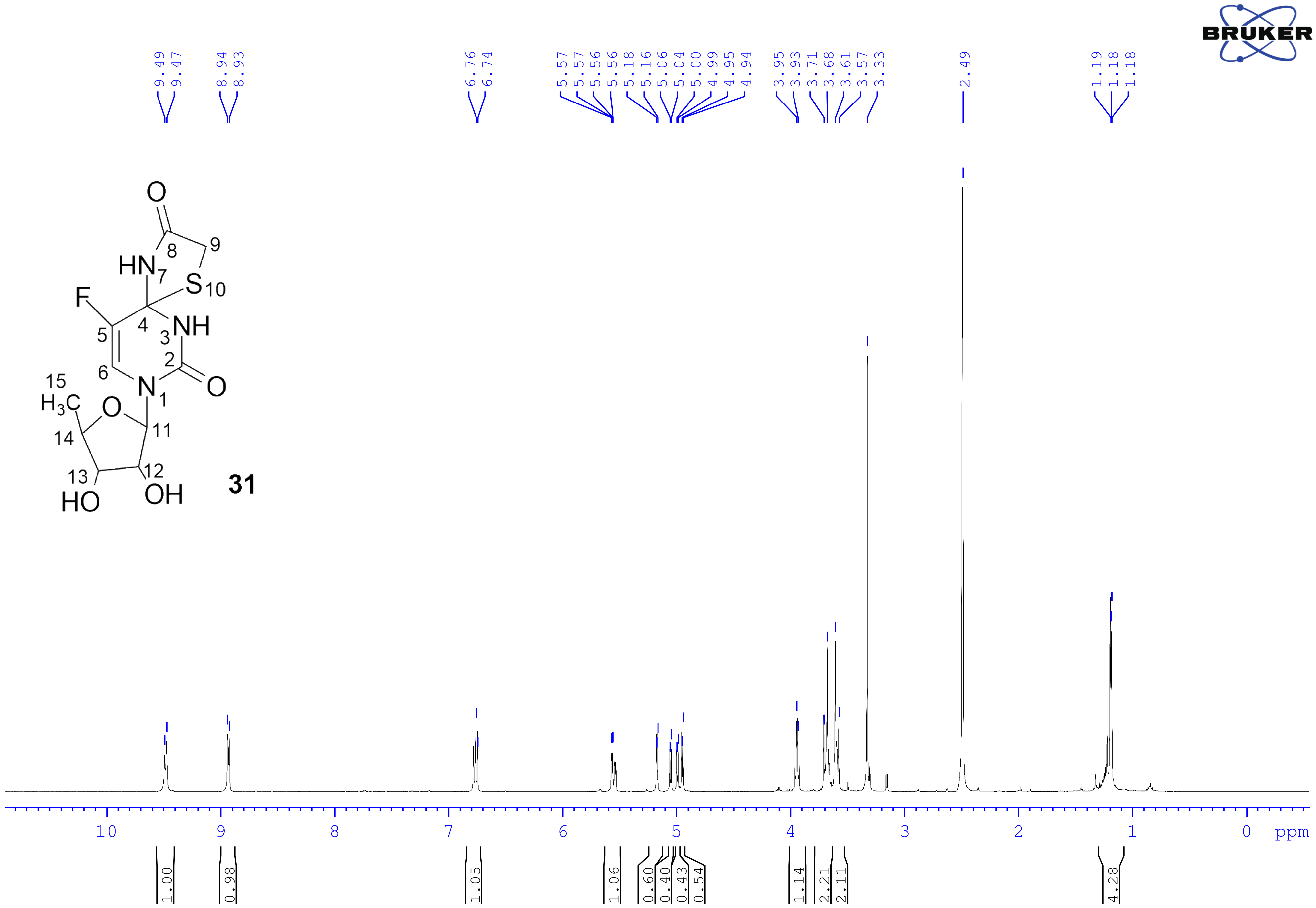
BRUKER
9.49
9.47
8.94
8.93
6.76
6.74
5.57
5.57
5.56
5.56
5.18
5.16
5.06
5.04
5.00
4.99
4.95
4.94
3.95
3.93
3.71
3.68
3.61
3.57
3.33
2.49
1.19
1.18
1.18
O
8
9
HN
7
S
10
F
5
4
3
NH
2
6
15
H3C
O
N
1
O
11
14
13
12
HO
OH
31
10
9
8
7
6
5
4
3
2
1
0
ppm
1.00
0.98
1.05
1.06
0.60
0.40
0.43
0.54
1.14
2.21
2.11
4.28

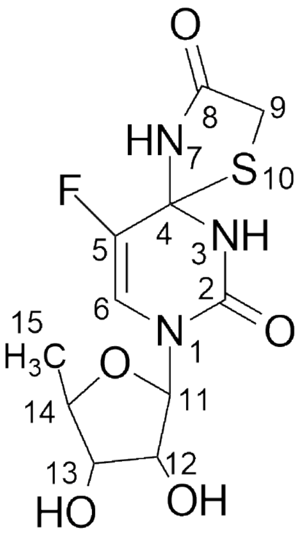

O
HN
7
8
9
S
10
F
4
5
3
NH
2
6
O
15
H_3C
N
1
O
11
14
13
12
HO
OH

31

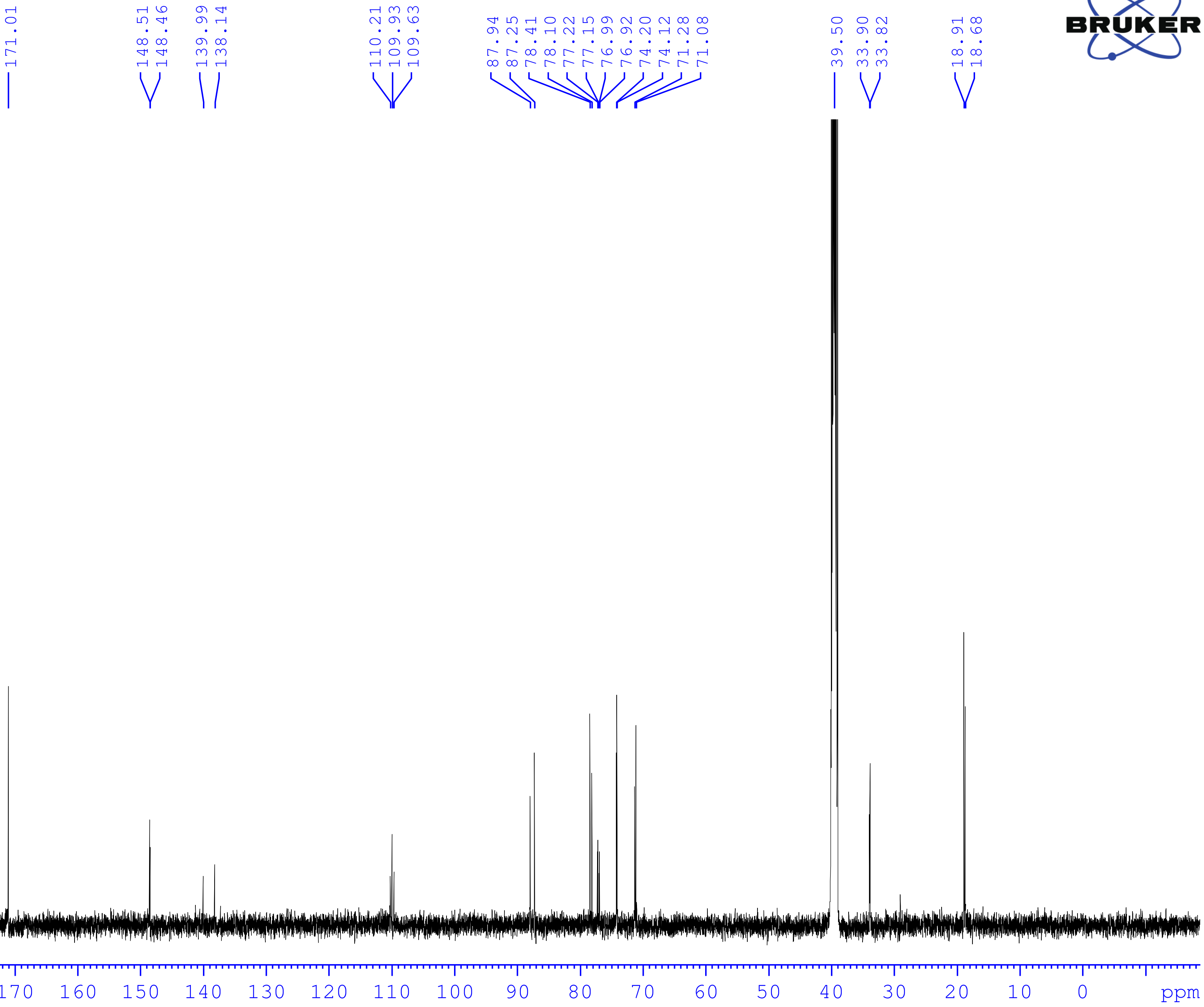

171.01
148.51
148.46
139.99
138.14
110.21
109.93
109.63
87.94
87.25
78.41
78.10
77.22
77.15
76.99
76.92
74.20
74.12
71.28
71.08
39.50
33.90
33.82
18.91
18.68
210 200 190 180 170 160 150 140 130 120 110 100 90 80 70 60 50 40 30 20 10 0 ppm
